# Silver-Mediated Electrosynthesis of Substituted Isoquinolines via Cyclization of 2‑Ethynylbenzaldehydes

**DOI:** 10.1021/acsomega.5c08865

**Published:** 2025-12-02

**Authors:** Eliakin S. de Borba, Daniel C. A. Amélio, Kíssylla P. Gomes, Pedro P. de Castro, Fernando R. Xavier, Guilherme M. Martins, Kleber T. de Oliveira, Samuel R. Mendes

**Affiliations:** † Department of Chemistry, State University of Santa Catarina (UDESC), Joinville, Santa Catarina 89219-719, Brazil; ‡ Department of Chemistry, Federal University of São Carlos (UFSCar), São Carlos, São Paulo 13565-905, Brazil; § Department of Pharmacy, 28113 Federal University of Juiz de Fora, Campus Governador Valadares, Governador Valadares, Minas Gerais 35010-177, Brazil

## Abstract

The electrosynthesis of substituted isoquinolines via silver-mediated intramolecular cyclization of 2-ethynylbenzaldehydes with ammonium acetate is presented. The reaction employs Ag­(+)|C(−) electrodes in a DMF/isopropanol mixture at 60 °C using LiClO_4_ as the supporting electrolyte, affording isoquinoline derivatives in yields up to 76%. Control experiments and DFT calculations were conducted to support a plausible reaction mechanism.

## Graphical abstract

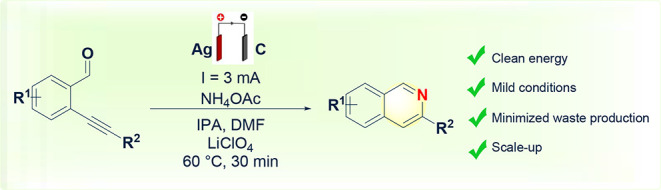

## Introduction

Isoquinoline derivatives always attract considerable attention due to their diverse biological activities, including antitumor, anti-inflammatory, and analgesic properties.^1–3^ Their frequent occurrence in natural products further underscores their relevance in drug discovery and the development of bioactive compounds.^4,5^ Structurally, isoquinolines are core components of various natural alkaloids, especially isoquinoline and tetrahydroisoquinoline alkaloids, which are classified into more than 20 subclasses. Among these, isoquinoline, benzylisoquinoline, protoberberine, and aporphine stand out for their wide natural distribution and notable pharmacological activities.^6,7^

The synthesis of isoquinoline derivatives has traditionally relied on intramolecular cyclization reactions catalyzed by transition metals such as Rh­(III), Pd­(II), Ag­(I), Ru­(II), Au­(I)^8–16^ and others.^17–19^ While these methods are generally effective, they present significant drawbacks, including the use of expensive and environmentally hazardous metal catalysts, harsh reaction conditions, and limited sustainability (Scheme 1A,B).^20,21^ These challenges have driven the pursuit of alternative synthetic strategies that are more economical, environmentally benign, and compatible with green chemistry principles.^22^

**Scheme 1:**
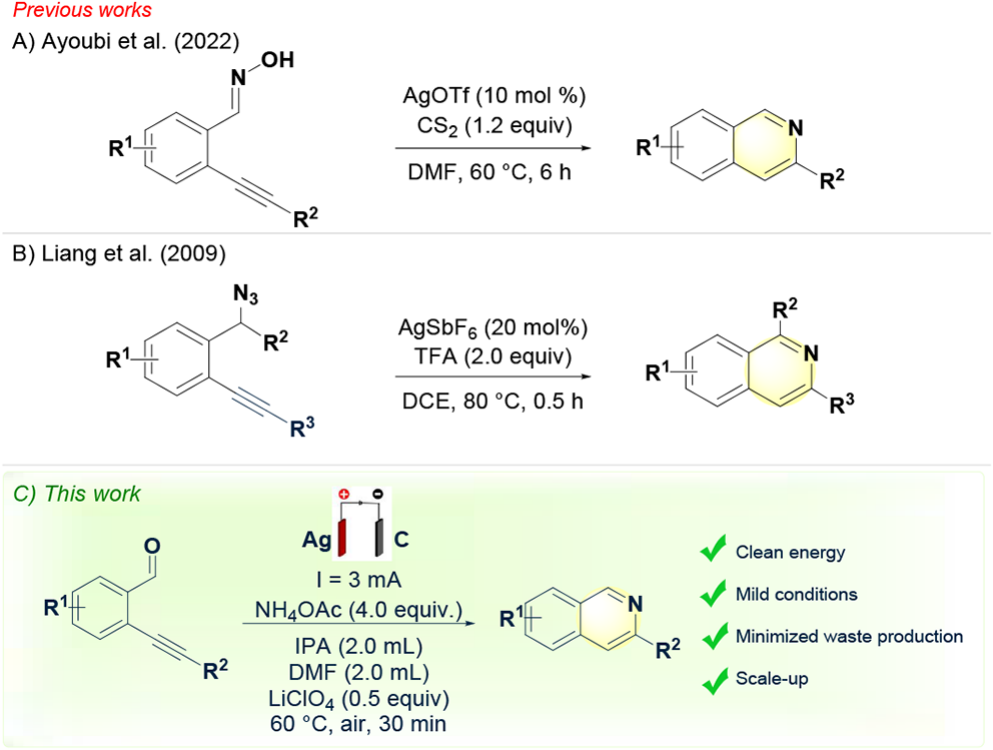
(A) Silver-Mediated Cyclization–Deoxygenation of 2-Alkynylbenzaldoximes. (B) Silver-Catalyzed Cyclization of 2-Alkynylbenzyl Azides. (C) This Work

In this context, electrosynthesis generally offers advantages over conventional methods, including a reduced use of highly aggressive reagents and shorter reaction times. Although the sustainability aspect is not absolute, the electrochemistry demonstrates improved efficiency and better atom economy, representing a valuable strategy for the construction of heterocycles,^23–27^ including isoquinolines.^28–31^ In the same way, it generally allows the use of cleaner solvents and reduces the need for aggressive reagents, offering a more efficient alternative.^32,33^ Thus, it not only overcomes the challenges associated with traditional methods but also achieves a superior atom economy.^34–36^

Among the strategies applicable to electrosynthesis, the use of metal anodes as sacrificial electrodes is particularly noteworthy.^37,38^ These electrodes release metal ions into the reaction medium, which can assist in substrate activation and promote selective intramolecular cyclization pathways, often relevant for the construction of heterocycles such as isoquinolines. Metals such as magnesium, copper, zinc, and silver are frequently used due to their low oxidation potential, which minimizes the formation of undesirable byproducts,^39,40^ enabling reactions to be carried out under milder conditions, with a reduced need for harsh reagents.^41–43^

Considering this, we report herein an efficient electrosynthetic methodology for the preparation of substituted isoquinolines, employing a silver electrode, without the need for complex ligands or external catalysts (Scheme 1C).

## Results and Discussion

In the initial optimization, several parameters were systematically evaluated, including solvent, electrode material, electric current, temperature, and reaction time. 2-(Phenylethynyl)­benzaldehyde (**1a**) was employed as the model substrate for the intramolecular electrochemical cyclization. Reactions were conducted in an undivided electrochemical cell equipped with a sacrificial silver anode and a graphite cathode. Under constant current electrolysis (3 mA) in acetonitrile (MeCN) at 60 °C for 60 min, the target isoquinoline **2a** was obtained in 58% isolated yield (Table 1, entry 2). Substituting MeCN with *N,N*-dimethylformamide (DMF) or isopropanol (IPA) (entries 3 and 4, respectively) afforded 64% and 68% yields, respectively. The role of the sacrificial silver anode was further investigated by replacing it with a graphite electrode. Under these conditions (entry 5), a drastic decrease in yield was observed, affording only 3% of **2a**. Considering these results, different sacrificial electrodes were evaluated (entry 6), specifically copper (Cu) and zinc (Zn), however, the formation of the desired product **2a** was not detected in either case, with only the corresponding imine observed. In the absence of electrical current (entry 7), the desired product was obtained in a low yield of 13% after 60 min. Applying a constant current of 6 mA (entry 8) increased the yield to a level comparable to that of entry 1. Variation of the reaction temperature to 25 and 80 °C (entries 9 and 10, respectively) led to decreased yields of 58% and 53%. Decreasing the concentration of ammonium acetate by 50% (entry 11) resulted in a further reduction in yield to 50%. Finally, substituting the nitrogen source with *tert*-butylamine (entry 12) also afforded the product **2a** in 56% yield after 6 h of reaction.

**Table 1:**
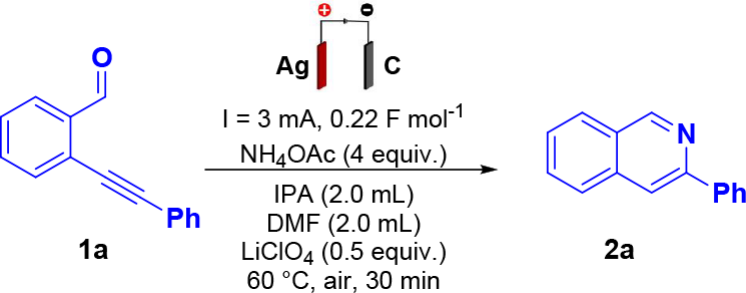
Optimization of the Reaction Conditions^a^

Entry	Deviation from standard conditions^a^	Isolated yield (%)
1	None	76
2	MeCN instead of DMF/IPA, 60 min	58
3	DMF instead of DMF/IPA	64
4	IPA instead of DMF/IPA	68
5	C(+)|C(−) instead of Ag(+)|C(−)	3
6	Cu, Zn instead of Ag(+)	0
7	Without current, 60 min	13
8	6 mA, 60 min	76
9	25 °C instead of 60 °C	58
10	80 °C instead of 60 °C	53
11	2 equiv. NH_4_OAc instead of 4 equiv.	50
12	*tert*-butylamine instead of NH_4_OAc in 6 h	56

a Conditions: **1a** (0.25 mmol), NH_4_OAc (1.0 mmol), LiClO_4_ (0.125 mmol), solvent (4.0 mL), using Ag­(+)|C(−)­electrodes, undivided cell, constant current = 3 mA (0.22 F mol^–1^), at 60 °C under air for 30 min.

After establishing the optimal electrochemical conditions, the substrate scope was examined for the intramolecular cyclization of a series of 2-(phenylethynyl)­benzaldehyde derivatives (Scheme 2). Substrates bearing electron-donating, electron-withdrawing, or alkyl groups underwent the transformation smoothly, affording the corresponding products in moderate to good isolated yields (58–76%). Halogenated derivatives furnished **2b** and **2c** in 62% and 72% yield, respectively, with the 4-chloro analog requiring an extended reaction time (2 h). The presence of a 4-methyl group afforded **2d** in 67% yield, while the 2-methoxy derivative **2e** was obtained in 66% yield.

**Scheme 2:**
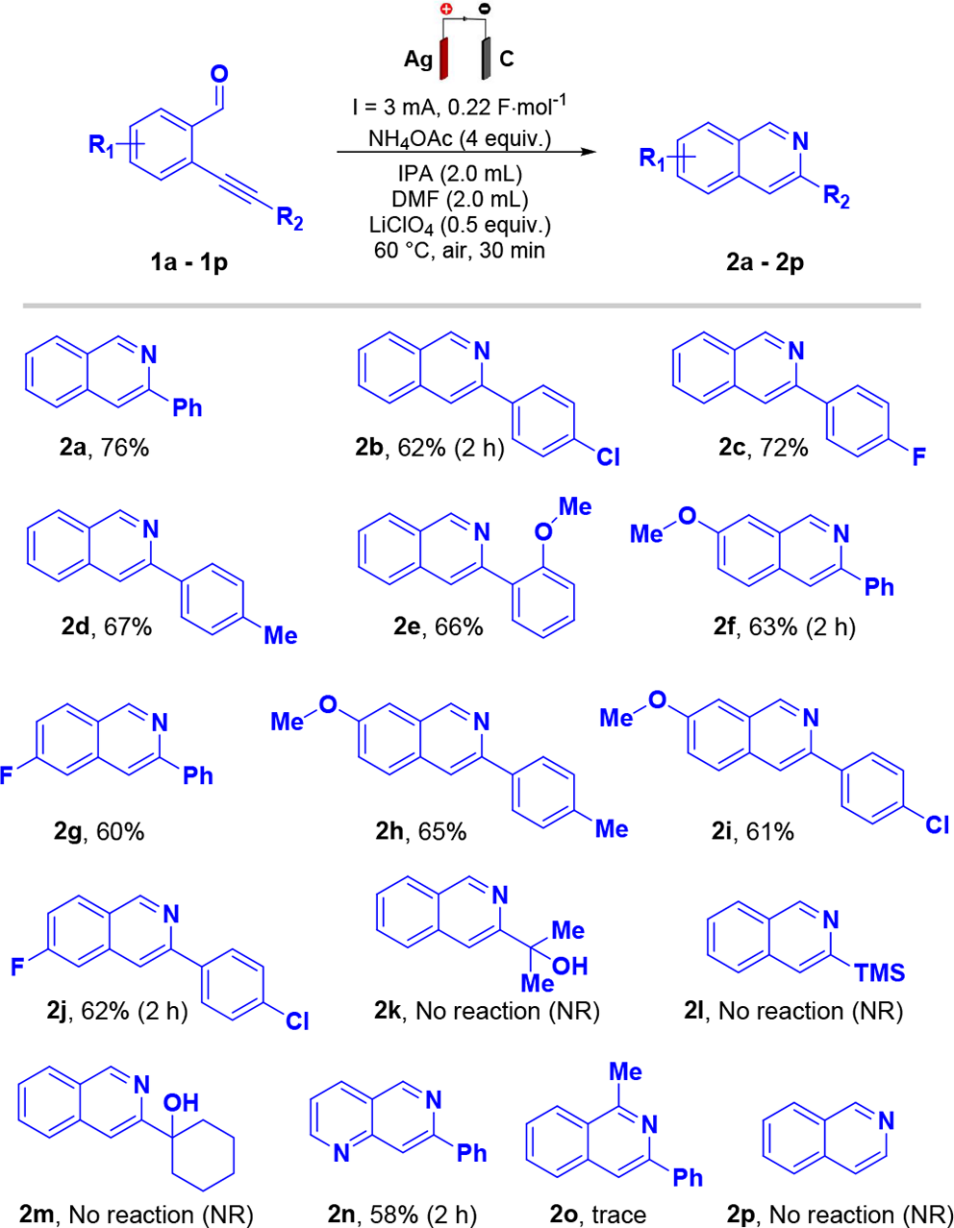
Scope of Synthesized Isoquinoline Derivatives

Substitution on the isoquinoline core was also well tolerated, as demonstrated by the formation of **2f** (OMe, 63% yield, 2 h) and **2g** (F, 60% yield). Additional substitution patterns delivered **2h** (OMe) in 65% yield, **2i** (Cl) in 61% yield, and **2j** (F) in 62% yield (2 h), confirming that electron-withdrawing substituents are compatible under the standard conditions. In contrast, no product formation was observed for hydroxylated (**2k**, **2m**) or silylated (**2l**) derivatives, suggesting that these functionalities may either inhibit silver-mediated oxidation or disrupt the key carbocationic intermediate required for ring closure. Similarly, the unsubstituted alkyne (**2p**) did not react, highlighting the importance of substituent electronic effects for cyclization. The methodology was also extended to heteroaromatic systems. The pyridine-containing precursor delivered isoquinoline **2n** in 58% yield after 2 h, indicating that the lower electron density of the heteroaryl ring slightly reduces reactivity under the standard electrochemical conditions. Finally, sterically hindered substrate **1o** afforded only trace amounts of **2o**.

The scalability of the proposed methodology was investigated through the intramolecular electrochemical cyclization of 2-(phenylethynyl)­benzaldehyde (**1a**), thus obtaining **2a** in up to 0.56 g (2.76 mmol). Under the same optimized reaction conditions previously applied on a smaller scale, the process afforded product **2a** with an isolated yield of 57% after 10 h (Scheme 3).

**Scheme 3:**
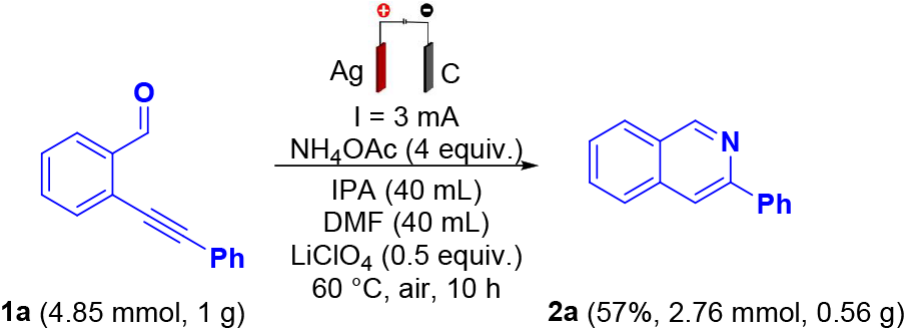
Scale-Up Experiment^a^

a Reaction conditions: Ag­(+)|C(−) electrodes, 2-(Phenylethynyl)­benzaldehyde 1a (4.85 mmol), LiClO_4_ (2.42 mmol, 0.5 equiv), DMF (40 mL), IPA (40 mL), constant current of 3 mA cm^–2^, under air conditions.

Although the yield was lower than that observed on the millimolar scale, the significant substrate conversion and preservation of reaction selectivity demonstrate the robustness and feasibility of the method under scaled-up conditions. In addition, this lower yield may be attributed to limitations inherent to the larger-scale reaction system, such as the distance between the electrodes, the reduced electrode surface-to-volume ratio, and less efficient mass transfer, which can limit electron transfer and slow reaction rates.

To gain deeper insights into the underlying reaction mechanism, a comprehensive series of control experiments was carried out (Scheme 4). Remarkably, the reaction proceeded efficiently even in the presence of TEMPO, a well-known radical scavenger, thereby providing strong evidence that the transformation does not proceed through a radical pathway (Scheme 4a). The catalytic role of silver was further investigated using different silver salts. To establish the optimal amount of salt, the extent of silver leaching during the electrochemical process was quantified and found to correspond to 10 mol %. This value was therefore selected as the standard ratio for subsequent experiments. Although these salts were capable of promoting the desired transformation, efficient performance required substantially higher silver loadings, with stoichiometric or near-stoichiometric amounts needed to achieve comparable conversions (Scheme 4b), even when inert graphite electrodes were employed as both anode and cathode (Scheme 4c). Finally, experiments using a divided electrochemical cell were conducted to identify the specific site of reactivity (Scheme 4d). The results confirmed that the critical transformation occurs at the anode and demonstrated that the presence of dissolved silver species is indispensable for efficient reaction progress. Collectively, these data underscore the pivotal role of anodic oxidation coupled with silver-mediated activation in the reaction mechanism, excluding radical intermediates and direct electrode-driven pathways.

**Scheme 4:**
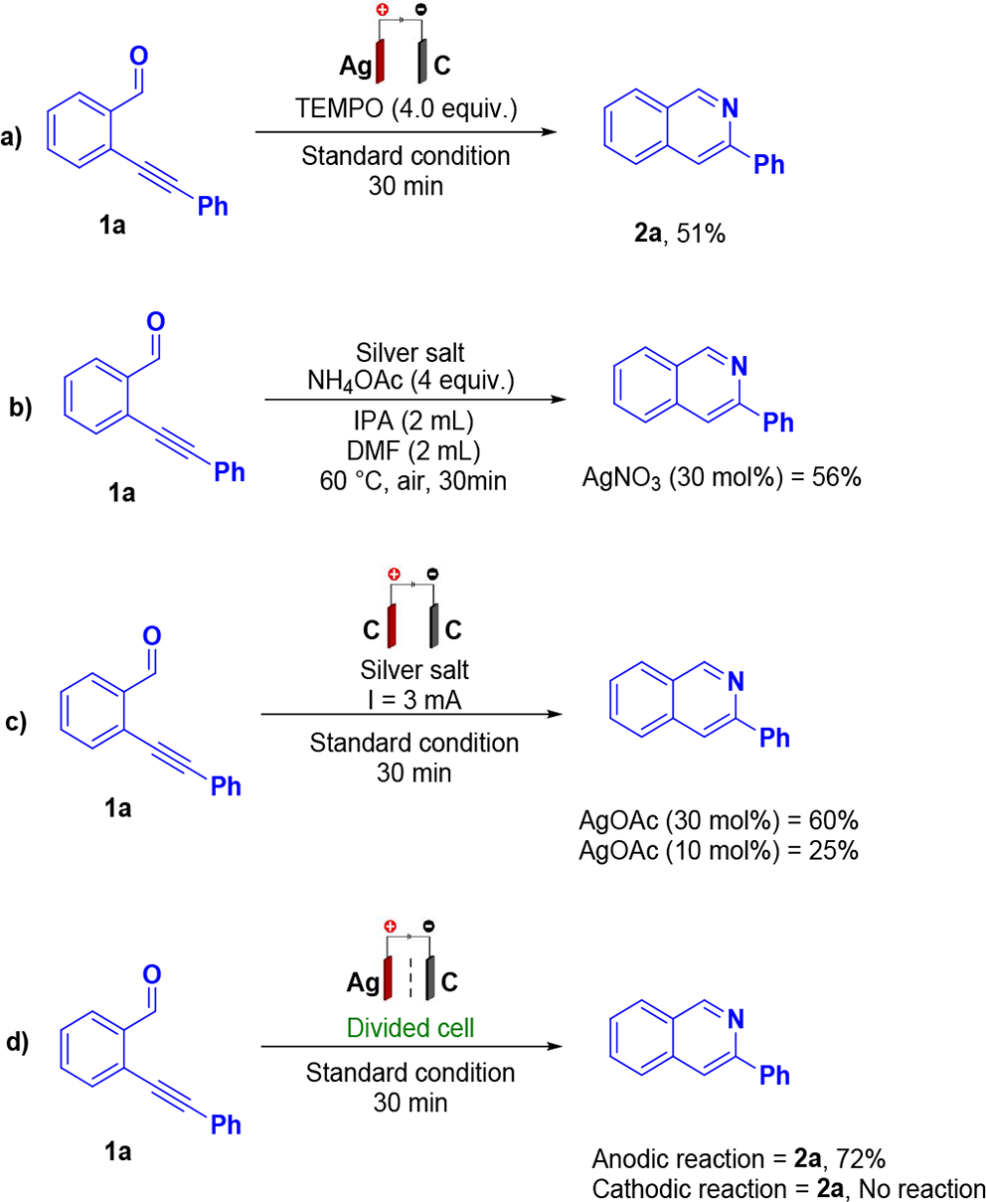
Control Experiments^a^ (A) Reaction with TEMPO (B) Reaction No-Current with Silver-Salt (C) Reaction with Silver-Salt and Current (D) Reaction Divided-Cell. Control Experiments^a^ (A) Reaction with TEMPO (B) Reaction No-Current with Silver-Salt (C) Reaction with Silver-Salt and Current (D) Reaction Divided-Cell[Fn sch4-fn2]

a Standard conditions: Ag­(+)|C(−) electrodes, 2-(Phenylethynyl)­benzaldehyde 1a (0.25 mmol), LiClO_4_ (0.125 mmol), DMF (2.0 mL), IPA (2.0 mL) constant current of 3 mA cm^–2^, under air.

To investigate the influence of the applied current on the reaction outcome, ON/OFF electrolysis experiments were performed under the optimized electrochemical conditions. The reaction proceeded efficiently while the current was applied (ON). In contrast, upon interruption of the current (OFF) resulted in the immediate interruption of product **2a** formation. Upon resuming the current (ON), the reaction restarted, affording more product **2a**. These observations indicate that maintaining a continuous electric current is necessary to drive the transformation, highlighting the pivotal role of electrochemical activation in the reaction mechanism (see Supporting Information for further details).

Additionally, the electrochemical response of compounds **1a** and **2a** was investigated by cyclic voltammetry (Figure 1) using a scan rate of 100 mV s^–1^. For both compounds, characteristic oxidation and reduction waves were observed, although no evidence of electrochemical reversibility was detected in the recorded processes. In the case of compound **1a**, an irreversible cathodic process with a reduction peak (*E*_pc_) at −1.63 V *vs* NHE was identified, suggesting a reduction step associated with degradation or irreversible transformation of the system. For the product **2a**, an equally irreversible anodic process with an oxidation peak (*E*_pa_) at 1.66 V *vs* NHE was observed, consistent with the formation of unstable oxidized species under the experimental conditions. However, considering that the anode leaches Ag° during the redox process, and the cathode promotes acetic acid reduction, both the starting materials (**1**) and the obtained products (**2**) are protected from direct redox processes at the electrodes.

**Figure 1:**
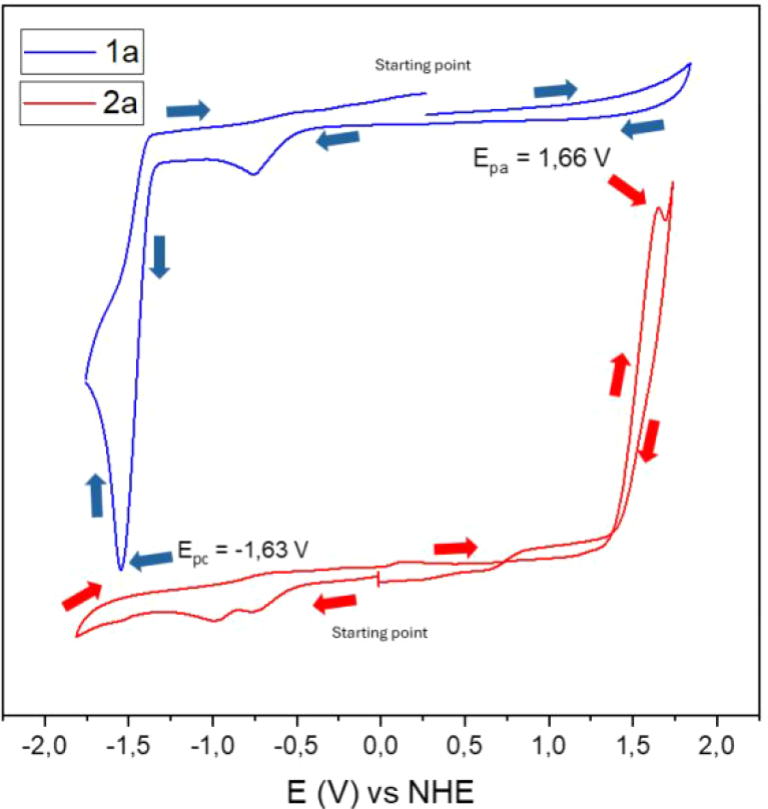
Normalized cyclic voltammograms of the compounds **1a** and **2a.** Conditions: Working Electrode—vitreous carbon; Reference Electrode Ag/Ag^+^; Auxiliary Electrode—Platinum Wire; Solvent: DMF:IPA (50% v/v). Supporting Electrolyte: LiClO_4_ (0.1 mol·L^–1^). Scan Rate: 100 mV s^–1^; Concentration: 2 × 10^–3^ mol L^–1^. Charting with IUPAC.

In an attempt to assess possible reversibility or stabilization of the redox processes, additional experiments were conducted by varying the scan rate between 25 and 500 mV s^–1^ (data not shown). However, these changes did not result in any significant improvement in electrochemical reversibility, indicating that the observed processes are inherently irreversible, possibly due to the rapid decomposition of intermediate species or the occurrence of coupled chemical reactions (EC processes). These results reinforce the complex electrochemical behavior of the studied compounds and their sensitivity to experimental conditions.

Theoretical calculations using Density Functional Theory (DFT) were performed to elucidate the mechanism. Considering the literature data, control experiments, and cyclic voltammetry, a plausible reaction pathway was proposed (Scheme 5).^44–47^ The mechanism begins with ammonium acetate (NH_4_OAc) undergoing an acid–base equilibrium, releasing ammonia, which subsequently reacts with the 2-ethynyl-substituted aldehyde to afford an imine intermediate **B**. Both steps are thermodynamically favorable (Δ*G* = −3.56 and −1.47 kcal mol^–1^, respectively) and proceed with notably low activation barriers (Δ*G* ^‡^ = 4.23 and 12.39 kcal mol^–1^, respectively). At the silver anode, anodic oxidation is accompanied by Ag^0^ dissolution, generating Ag^+^ species *in situ*. Next, the imine **B** coordinates to Ag^+^ via π-system of the alkyne, promoting electrophilic activation and facilitating intramolecular cyclization through a *6-endo-dig* pathway, yielding a cationic σ-Ag metallacyclic intermediate **C** (Δ*G* ^‡^ = 3.10 kcal mol^–1^ and Δ*G* = −40.88 kcal mol^–1^) (see the Supporting Information).

**Scheme 5:**
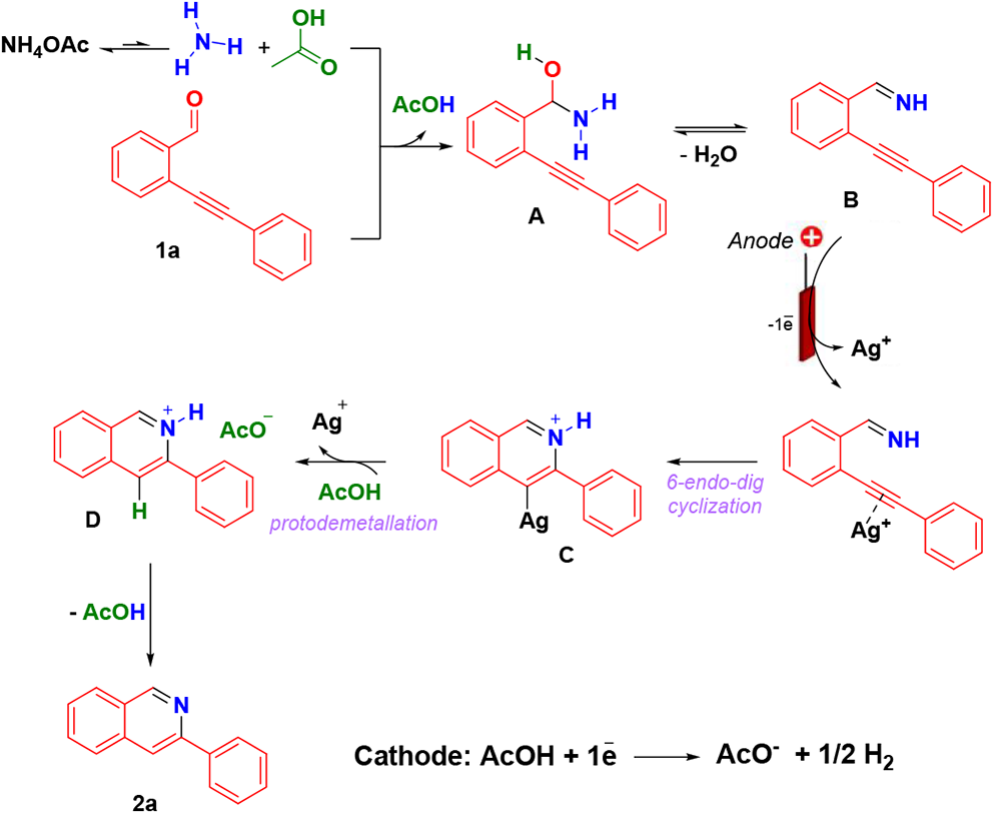
Plausible Reaction Mechanism

According to Baldwin’s rules, the *5-exo-dig* cyclization would also be possible and could, in principle, compete with the *6-endo-dig* process. However, additional calculations carried out for this alternative pathway revealed a slightly higher activation barrier (Δ*G* ^‡^ = 5.40 kcal mol^– 1^). Although the energy difference between the two transition states is modest (ΔΔ*G* ^‡^ = 2.3 kcal mol^– 1^), it is sufficient to result in the essentially exclusive formation of the *6-endo-dig* product.

The metallacyclic intermediate undergoes an AcOH-mediated protodemetalation, releasing the protonated isoquinoline product **D** (Δ*G* ^‡^ = 17.61 kcal mol^–1^; Δ*G* = −12.96 kcal mol^–1^). A subsequent thermodynamically favorable proton-transfer step (Δ*G* = −19.74 kcal mol^–1^) furnishes acetic acid, regenerates the Ag^+^ species, and delivers the neutral isoquinoline product **2a**. Notably, AcOH plays multiple critical roles in the process, including mediating imine formation and reduction at the cathode, protecting the cell to prevent parallel reactions.

During the reaction, the catalytic cycle is maintained through the continuous generation of Ag^+^ at the anode, controlled formation and consumption of the imine intermediate, and efficient proton and electron transfer between electrodes.

## Conclusions

An efficient electrochemical protocol was developed for the synthesis of substituted isoquinolines via silver-mediated intramolecular cyclization of 2-ethynylbenzaldehydes with ammonium acetate. Using Ag­(+)|C(−) electrodes in a DMF/IPA solvent mixture at 60 °C, this method notably provided isoquinoline derivatives in yields of up to 76%, demonstrating good functional group tolerance. The robustness and scalability of the process were confirmed through gram-scale reactions. ON/OFF electrolysis experiments confirmed the essential role of continuous current, as product formation stopped when the current was paused and resumed upon reapplication. Mechanistic and electrochemical studies, supported by density functional theory calculations (DFT), revealed a nonradical anodic pathway involving silver activation of the alkyne.

## Experimental Section

### General Information

All purchased chemicals were used as received without further purification. Analytical TLC was performed on TLC plates (silica gel 60 F254) and visualized employing a UV lamp and/or acidic ethanolic vanillin solution (5% in 10% H_2_SO_4_) as a revelator. Yields refer to purified compounds that are spectroscopically pure. Both ^1^H and ^13^C­{^1^H} NMR spectra were recorded at 400 and 100 MHz, respectively. Chemical shifts are reported in ppm downfield from the signal of TMS, used as an internal standard, and the coupling constants (*J*) are expressed in Hertz (Hz). Chemical shifts are reported employing the following abbreviation pattern: s (singlet), d (doublet), dd (doublet of doublet), dt (doublet of triplet), t (triplet), q (quartet), and m (multiplet). Low-resolution mass spectra were obtained from a Shimadzu GC-MS-QP2020 NX mass spectrometer.

The calculations were carried out using the Gaussian 09 package (revision D01).^48^ The density functional theory (DFT) was employed in the optimization of the structures of all molecular complexes and transition states (TSs). The M06–2X functional (grid = ultrafine), the LanL2DZ basis set and the solvation model based on density (SMD) for *N,N*-dimethylformamide were used in the calculations.^49^ The TSs were optimized using the Berny algorithm, presented a single imaginary frequency and were fully characterized through the analysis of the intrinsic reaction coordinate (IRC).^50^ The thermodynamic properties were calculated using a temperature of 333.15 K (60 °C) (proposals 1–4, ESI file) or 263.15 K (−10 °C) (proposal 5, ESI file) and a pressure of 1.0 atm, aiming to reproduce the experimental conditions. The vibrational analysis of each species was carried out to confirm the identity of all stationary points and in the determination of the thermal corrections to enthalpy and Gibbs free energy.

Substituted 2-ethynylbenzaldehydes were prepared following literature protocols.^51–53^ The electrochemical reactions were carried out using a power supply (AFR—model FA3005P).

### General Procedure for the Synthesis of 2a–2o

The electrochemical reactions were carried out in an undivided cell of 10 mL (glass bottle with plastic screw cap) equipped with a silver anode (99.9% – 55 mm × 5 mm) and a carbon cathode (51 mm × 8 mm). The 2-ethynylbenzaldehyde (**1a–o**), dimethylformamide (DMF) (2.0 mL), isopropyl alcohol (IPA) (2.0 mL), ammonium acetate (NH_4_OAc) (1.0 mmol, 77 mg), and lithium perchlorate (LiClO_4_) (0.125 mmol, 13 mg) were added to the electrochemical cell. A 3.0 mA constant current was applied at 60 °C for 30 min. After this period, the current was turned off, the electrodes were removed, and 5.0 mL of methanol, along with 1 mL of a saturated aqueous solution of sodium bisulfite were added. The mixture was stirred for an additional 5 min. It was then extracted with a 10% ethyl acetate solution in hexane (4 × 20 mL) and dried over MgSO_4_. Finally, the solvent was removed under reduced pressure using a high-vacuum pump. Purification was carried out by column chromatography using a 10% ethyl acetate in hexane solution as the eluent.

#### 3-Phenylisoquinoline (2a)^54^

Yellow solid, (38.9 mg, 0.19 mmol, 76% yield), m.p.: 95.1 – 96.2 °C. The reaction was purified through column chromatography on silica gel flash (elution: hexane and ethyl acetate = 90:10). ^1^H NMR (400 MHz; CDCl_3_): δ = 9.32 (s, 1H), 8.13–8.12 (m, 2H), 8.04 (s, 1H), 7.97–7.95 (d, *J* = 8.1 Hz, 1H), 7.85–7.83 (d, *J* = 8.1 Hz, 1H), 7.69–7.65 (m, 1H), 7.58 (m, 1H), 7.52–7.48 (m, 2H), 7.42–7.39 (m, 1H). ^13^C­{^1^ H} NMR (100 MHz; CDCl_3_): δ = 152.4, 151.3, 139.6, 136.6, 130.5, 128.8, 128.5, 127.7, 127.5, 127.0, 127.0, 126.9, 116.5; CG-EM (*m*/*z*; rel. int. %): 206 (M^+^; 16), 205 (100), 204 (56), 203 (10), 177 (8), 176 (13), 102 (23).

#### 3-(4-Chlorophenyl)­isoquinoline (2b)^55^

Yellow solid, (36.6 mg, 0.15 mmol, 51% yield), m.p.: 141.7 – 142.4 °C. The reaction was purified through column chromatography on silica gel flash (elution: hexane and ethyl acetate = 90:10). ^1^H NMR (400 MHz; CDCl_3_): δ = 9.32 (s, 1H), 8.09–8.06 (m, 2H), 8.04 (s, 1H), 8.00 – 7.98 (d, *J* = 8.1 Hz, 1H), 7.88–7.86 (d, *J* = 8.3 Hz, 1H), 7.73–7.68 (m, 1H), 7.62–7.58 (m, 1H), 7.48–7.46 (m, 2H). ^13^C­{^1^ H} NMR (100 MHz; CDCl_3_): δ = 152.5, 150.0, 138.0, 136.6, 134.6, 130.7, 128.9, 128.2, 127.8, 127.6, 127.3, 126.9, 116.4; CG-EM (*m*/*z*; rel. int. %): 241 (M^+2^; 34), 240 (M^+^; 24), 239 (100), 238 (23), 204 (48), 203 (22), 176 (17), 102 (31), 88(18).

#### 3-(4-Fluorophenyl)­isoquinoline (2c)^55^

Brown solid, (40.1 mg, 0.18 mmol, 72% yield), m.p.: 120.2 – 121.4 °C. The reaction was purified through column chromatography on silica gel flash (elution: hexane and ethyl acetate = 90:10). ^1^H NMR (400 MHz; CDCl_3_): δ = 9.32 (s, 1H), 8.13–8.09 (m, 2H), 8.02 (s, 1H), 8.01 – 7.99 (d, *J* = 8.28 Hz, 1H), 7.88 – 7.86 (d, *J* = 8.09 Hz, 1H), 7.73–7.69 (m, 1H), 7.61–7.58 (m, 1H), 7.26–7.17 ppm (m, 2H). ^13^C­{^1^ H} NMR (100 MHz; CDCl_3_): δ = 163.4 (d, *J* ^1^_C(Ar)‑F_ = 248.1 Hz), 151.4, 149.3, 135.6, 134.7 (d, *J* ^4^_C(Ar)‑F_ = 3.1 Hz), 129.6, 127.7 (d, *J* ^3^_C(Ar)‑F_ = 8.1 Hz), 127.6, 126.6, 126.5, 126.1, 115.1, 114.7 (d, *J* ^2^_C(Ar)‑F_ = 21.4 Hz) ppm. CG-EM (*m*/*z*; rel. int. %): 224 (m^+^; 17), 223 (100), 222 (60), 221 (7), 194 (7), 175 (5), 111 (14), 101 (8), 97 (5).

#### 3-(*p*-Tolyl)­isoquinoline (2d)^15^

Pale yellow solid, (36.6 mg, 0.16 mmol, 67% yield), m.p.: 68.9 – 69.8 °C. The reaction was purified through column chromatography on silica gel flash (elution: hexane and ethyl acetate = 90:10). ^1^H NMR (400 MHz, CDCl_3_): δ = 9.32 (s, 1H), 8.03–8.01 (m, 3H), 7.97 (dd, *J* = 9.22 Hz, 1.01 Hz, 1H), 7.83 (dd, *J* = 9.11 Hz, 0.86 Hz 1H), 7.68–7.64 (m, 1H), 7.57–7.55 (m, 1H), 7.32 – 7.30 (d, *J* = 8.0 Hz, 2H), 2.42 (s, 3H). ^13^C­{^1^ H} NMR (100 MHz; CDCl_3_): δ = 152.3, 151.3, 138.4, 136.8, 136.7, 130.4, 129.5, 127.6, 127.5, 126.8, 116.0, 21.2. CG-EM (*m*/*z*; rel. int. %): 219 (100), 220 (17) 218 (50), 217 (20), 108 (18).

#### 3-(2-Methoxyphenyl)­isoquinoline (2e)^56^

Orange solid, (38.7 mg, 0.16 mmol, 66% yield), m.p.: 67.7 – 68.7 °C. The reaction was purified through column chromatography on silica gel flash (elution: hexane and ethyl acetate = 90:10). ^1^H NMR (400 MHz; CDCl_3_): δ = 9.33 (s, 1H) 8.20 (s, 1H), 7.97–7.96 (d, 1H), 7.92–7.90 (d, 1H), 7.84–7.82 (d, 1H), 7.67–7.63 (m, 1H), 7.57–7.53 (m, 1H), 7.37–7.35 (m, 1H), 7.13–7.09 (m, 1H), 7.04–7.02 (d, 1H), 3.88 (s, 3H). ^13^C­{^1^ H} NMR (100 MHz; CDCl_3_): δ = 157.1, 151.9, 149.3, 136.2, 131.4, 130.2, 129.5, 129.2, 127.4, 127.0, 126.9, 126.9, 121.1, 121.0, 111.5, 55.7; CG-EM (*m*/*z*; rel. int. %): 236 (m^+^; 11), 235 (71), 234 (100), 218 (13), 207 (18), 206 (47), 205 (44), 204 (67), 165 (11), 130 (47), 102 (44).

#### 7-Methoxy-3-phenylisoquinoline (2f)^55^

Yellow solid, (29.9 mg, 0.12 mmol, 63% yield, 2 h), m.p.: 144.8 – 145.4 °C. The reaction was purified through column chromatography on silica gel flash (elution: hexane and ethyl acetate = 90:10). ^1^H NMR (400 MHz; CDCl_3_): δ = 9.24 (s, 1H), 8.10–8.08 (m, 2H), 8.00 (s, 1H), 7.79–7.76 (d, *J* = 9.0 Hz, 1H), 7.51–7.47 (m, 2H), 7.41–7.37 (m, 1H), 7.36–7.33 (m, 1H), 7.24 (d, *J* = 2.43 Hz, 1H), 3.96 ppm (s, 3H). ^13^C­{^1^ H} NMR (100 MHz; CDCl_3_): δ = 158.4, 150.9, 149.7, 139.7, 132.3, 128.9, 128.7, 128.4, 128.1, 126.7, 123.7, 116.4, 104.7, 55.5 ppm; CG-EM (*m*/*z*; rel. int. %): 236 (M^+^; 19), 235 (100), 220 (35), 192 (43), 191 (14), 190 (8), 165 (28), 164 (8), 117 (9), 95 (8).

#### 6-Fluoro-3-phenylisoquinoline (2g)^55^

White solid, (33.4 mg, 0.15 mmol, 60% yield), m.p.: 123.6 – 124.8 °C. The reaction was purified through column chromatography on silica gel flash (elution: hexane and ethyl acetate = 90:10). ^1^H NMR (400 MHz, CDCl_3_): δ = 9.30 (s, 1H), 8.12 – 8.10 (d, *J* = 7.7 Hz, 2H), 8.02 – 7.99 (m, 2H), 7.54 – 7.43 (m, 4H), 7.37 – 7.34 (m, 1H). ^13^C­{^1^ H} NMR (100 MHz; CDCl_3_): δ = 164.7 (d, *J* ^1^_C(Ar)‑F_ = 252.8 Hz), 152.1 152.0, 139.2, 138.2 (d, *J* ^3^_C(Ar)‑F_ = 10.1 Hz), 130.7 (d, *J* ^3^_C(Ar)‑F_ = 9.7 Hz), 128.8, 127.0, 125.0, 117.7 (d, *J* ^2^_C(Ar)‑F_ = 25.8 Hz), 116.0 (d, *J* ^4^_C(Ar)‑F_ = 5.4 Hz), 110.3 (d, *J* ^4^_C(Ar)‑F_ = 21.0 Hz). CG-EM (*m*/*z*; rel. int. %): 224 (M^+^; 15), 223 (100), 222 (70), 221 (8), 195 (4), 194 (6), 175 (4), 111 (15), 101 (5), 97 (5).

#### 7-Methoxy-3-(*p*-tolyl)­isoquinoline (2h)^16^

White solid, (40.4 mg, 0.16 mmol, 65% yield), m.p.: 140.8 – 141.3 °C. The reaction was purified through column chromatography on silica gel flash (elution: hexane and ethyl acetate = 90:10). ^1^H NMR (400 MHz; CDCl_3_): δ = 9.21 (s, 1H), 8.00–7.96 (m, 3H), 7.75–7.73 (d, *J* = 8.98 Hz, 1H), 7.34–7.29 (m, 3H), 7.21 (d, *J* = 2.45 Hz, 1H), 3.95 (s, 3H), 2.41­(s, 3H). ^13^C­{^1^ H} NMR (100 MHz; CDCl_3_): δ = 158.2, 150.8, 149.7, 138.0, 136.9, 132.3, 129.5, 128.7, 128.4, 126.6, 123.7, 115.9, 104.7, 55.5, 21.2. CG-EM *m*/*z*: 250 (M^+^; 19), 249 (100), 235 (7), 234 (37), 206 (29), 205 (10), 204 (9), 191 (7), 124 (8), 102 (8).

#### 3-(4-Chlorophenyl)-7-methoxyisoquinoline (2i)^57^

White solid, (41.0 mg, 0.15 mmol, 61% yield), m.p.: 148.7 – 149.2 °C. The reaction was purified through column chromatography on silica gel flash (elution: hexane and ethyl acetate = 90:10). ^1^H NMR (400 MHz, CDCl_3_): δ = 9.21 (s, 1H), 8.05 – 8.03 (m, 2H), 7.97 (s, 1H), 7.77 (d, *J* = 8.96 Hz, 1H), 7.47–7.44 (m, 2H), 7.37 (dd, *J* = 8.96, 6.44 Hz, 1H), 7.24 (d, J = 2.37 Hz, 1H), 3.97 (s, 3H). ^13^C­{^1^ H} NMR (100 MHz, CDCl_3_) δ = 158.6, 151.0, 148.4, 138.2, 134.2, 132.2, 129.0, 128.9, 128.4, 127.9, 123.9, 116.3, 104.7, 100.0, 55.5; CG-EM (*m*/*z*; rel. int. %): 271 (M^+2^; 32), 270 (M^+^; 18), 269 (100), 256 (11), 254 (35), 228 (10), 226 (31), 191 (37), 190 (13), 164 (15).

#### 3-(4-Chlorophenyl)-6-fluoroisoquinoline (2j)

Brown solid, (39.9 mg, 0.15 mmol, 62% yield, 2 h), m.p.: 108.7 – 110.3 °C. The reaction was purified through column chromatography on silica gel flash (elution: hexane and ethyl acetate = 90:10). ^1^H NMR (400 MHz, CDCl_3_): δ = 9.28 (s, 1H), 8.07 – 8.05 (m, 2H), 8.02 – 7.99 (m, 2H), 7.49 – 7.45 (m, 3H), 7.38 – 7.34 (td, *J* = 8.7, 2.5 Hz, 1H). ^13^C­{^1^ H} NMR (100 MHz; CDCl_3_): δ = 164.8 (d, *J* ^1^_C(Ar)‑F_ = 253.1 Hz), 152.1 150.9, 138.1 (d, *J* ^3^_C(Ar)‑F_ = 10.9 Hz), 137.6, 134.9, 130.6 (d, *J* ^3^_C(Ar)‑F_ = 10.0 Hz), 129.0, 128.3, 125.0, 118.0 (d, *J* ^2^_C(Ar)‑F_ = 25.7 Hz), 115.9 (d, *J* ^4^_C(Ar)‑F_ = 5.3 Hz), 110.3 (d, *J* ^4^_C(Ar)‑F_ = 21.1 Hz). CG-EM (*m*/*z*; rel. int. %): 259 (M^+2^; 32), 258 (M^+^, 23), 257 (100), 256 (22), 223 (6), 222 (43), 221 (18), 194 (7), 111 (24), 97 (11). HRMS (ESI­(+)-TOF) *m*/*z*: [M + H]^+^ Calcd for C_15_H_9_ClFN^+^ 258.0480; Found 258.0483.

#### 7-Phenyl-1,6-naphthyridine (2n)^58^

Orange solid, (29.8 mg, 0.14 mmol, 58% yield), m.p.: 133.5 °C – 134.3 °C. The reaction was purified through column chromatography on silica gel flash (elution: hexane and ethyl acetate = 90:10). ^1^H NMR (400 MHz, CDCl_3_): δ 9.35 (s, 1H), 9.10 (dd, *J* = 4.5, 1.7 Hz, 1H), 8.35 (s, 1H), 8.31 – 8.29 (dd, *J* = 8.2, 1.7 Hz, 1H), 8.19 – 8.17 (m, 2H), 7.55 – 7.44 (m, 4H). ^13^C­{^1^ H} NMR (100 MHz, CDCl_3_) δ = 155.2, 155.0, 152.7, 151.4, 138.8, 135.6, 132.0, 129.2, 128.9, 127.2, 122.7, 122.2, 117.8. CG-EM (*m*/*z*; rel. int. %): 207 (M^+^; 15), 206 (100), 205 (65), 179 (6), 178 (8), 151 (9), 103 (13), 102 (9), 89 (8), 76 (8).

## Supplementary Material



## Data Availability

The data underlying this study are available in the published article and its Supporting Information.

## Notes

General procedure; scaling up synthesis; general procedure for cyclic voltammetry; control experiments; characterization data of products; 1H and 13C NMR spectra of all synthesized compounds; HRMS and theoretical calculations (PDF)
