# Supplementary material for: Silver-Mediated Electrosynthesis of Substituted Isoquinolines via Cyclization of 2‑Ethynylbenzaldehydes
Source: ACS Omega. 2025 Dec 2;10(49):60744–51. doi: 10.1021/acsomega.5c08865 (PMC12713483; doi:10.1021/acsomega.5c08865)
Supplement: Supplementary file 1 [file ao5c08865_si_001.pdf]

Silver-Mediated Electrosynthesis of Substituted Isoquinolines via Cyclization of 2-Ethynylbenzaldehydes

Eliakin S. de Borba[‡a,b], Daniel C. A. Amélio[a], Kíssylla P. Gomes[c], Pedro P. de Castro[c], Fernando R. Xavier[a], Guilherme M. Martins*[b], Kleber T. de Oliveira*[b], Samuel R. Mendes*[a]

Table of Contents

1. GENERAL CONSIDERATIONS........2

2. GENERAL PROCEDURE........3

2.1 Compounds 2a-2p........3

3. SCALING UP SYNTHESIS PROCEDURE FOR 2a........4

4. GENERAL PROCEDURE FOR CYCLIC VOLTAMMETRY........5

5. CONTROL EXPERIMENTS........6

6. ELECTRIC CHARGE........8

7. CHARACTERIZATION DATA OF PRODUCTS........9

7.1 Compounds 2a-2p........9

8. NMR SPECTRA........13

8.1 Compounds 2a-2o........13

9. THEORETICAL CALCULATIONS........24

9.1 General overview of the evaluated mechanisms........24

9.2 Energy profile (ΔG) of the evaluated mechanisms........26

9.3 Imaginary frequencies for all transition states and intrinsic reaction coordinates........27

9.4 Electronic energies (E), enthalpies (H) and Gibbs free energies (G) of all optimized structures........30

9.5 Electronic energies (ΔE), enthalpies (ΔH) and Gibbs free energies (ΔG) variation along the reaction pathway........32

9.6 Coordinates of optimized stationary points........33

10. REFERENCES........41

1. GENERAL CONSIDERATIONS

All purchased chemicals were used as received without further purification. Analytical TLC was performed on TLC plates (silica gel 60 F254) and visualized employing a UV lamp and/or acidic ethanolic vanillin solution (5% in 10% H_2SO_4) as a revelator. Yields refer to purified compounds that are spectroscopically pure. Both ^{1}H and ^{13}C {^{1}H} NMR spectra were recorded at 400 and 100 MHz, respectively. Chemical shifts are informed in ppm downfield from the signal of TMS, used as an internal standard, and the coupling constants (*J*) are expressed in Hertz (Hz). Chemical shifts are reported employing the following abbreviation pattern: s (singlet), d (doublet), dd (doublet of doublet), dt (doublet of triplet), t (triplet), q (quartet), and m (multiplet). Low-resolution mass spectra were obtained from a Shimadzu GC-MS-QP2020 NX mass spectrometer.

Substituted 2-ethynylbenzaldehydes were prepared following literature protocols.[1–3] The electrochemical reactions were carried out using a power supply (AFR – model FA3005P).

Figure S1. The power source used in the electrochemical reactions.

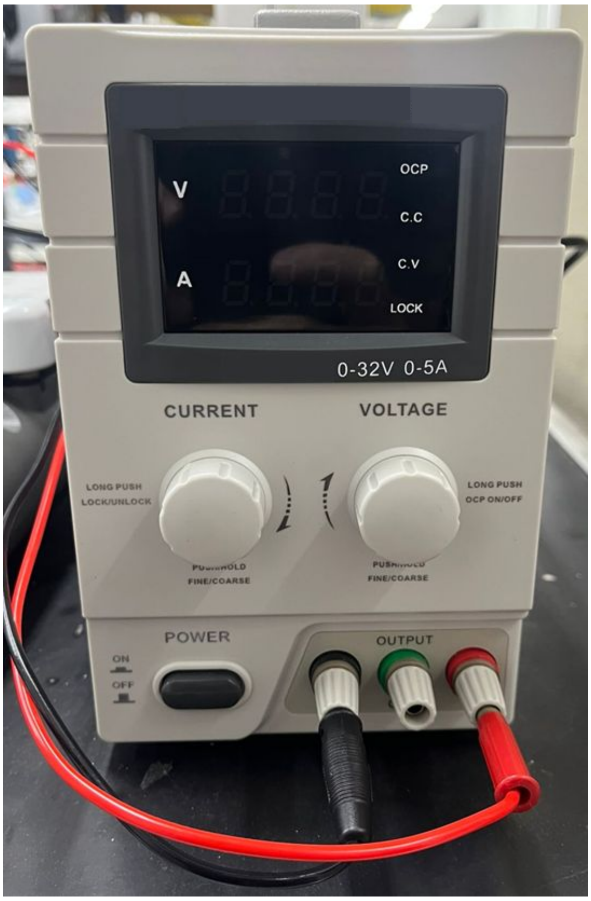

2. GENERAL PROCEDURE

2.1 Compounds 2a-2p

The electrochemical reactions were carried out in an undivided cell of 10 mL (glass bottle with plastic screw cap) equipped with a silver anode (99.9% - 55 mm x 5 mm) and a carbon cathode (51 mm x 8 mm), as shown in **Figure S2**. The 2-ethynylbenzaldehyde (**1**), dimethylformamide (DMF) (2.0 mL), isopropyl alcohol (IPA) (2.0 mL), ammonium acetate (NH_4OAc) (1.0 mmol, 77 mg), and lithium perchlorate ($LiClO_4$) (0.125 mmol, 13 mg) were added to the electrochemical cell. A constant current of 3.0 mA was applied at 60 °C for 30 minutes. After this period, the current was turned off, the electrodes were removed, and 5 mL of methanol along with 1.0 mL of a saturated aqueous solution of sodium bisulfite were added. The mixture was stirred for an additional 5 minutes. It was then extracted with a 10% ethyl acetate solution in hexane (4 × 20 mL) and dried over $MgSO_4$. Finally, the solvent was removed under reduced pressure using a high-vacuum pump, yielding a reddish-brown crystalline solid. Purification was carried out by column chromatography using a 10% ethyl acetate in hexane solution as the eluent, to afford the product as a white crystalline solid.

Figure S2. Electrochemical cell and reaction setup for **2a-2p** compounds.

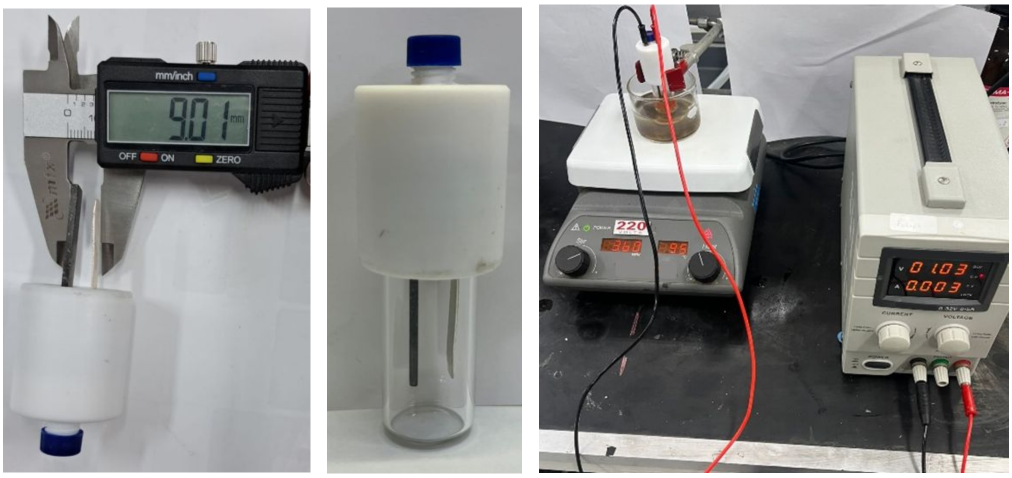

Figure S3. General scheme for the synthesis of **2a-2o** compounds.

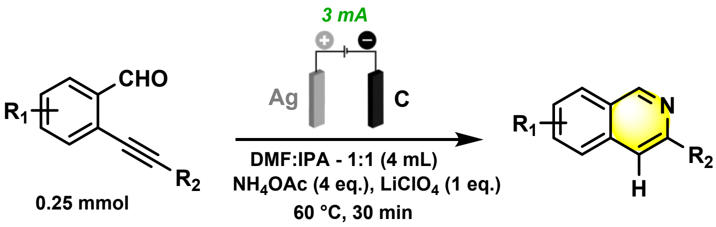

3. SCALING UP SYNTHESIS PROCEDURE FOR 2a

The scaling up of the electrochemical reaction was carried out in an undivided cell of 100 mL (glass bottle with plastic screw cap) equipped with three silver anodes (99.9% - 70 mm x 5 mm, totaling 70 mm x 15 mm) and one carbon cathode (53 mm x 25 mm), as shown in **Figure S4**. Inside the electrochemical cell, 2-(phenylethynyl)benzaldehyde (**1a**) (4.85 mmol – 1.00 g), dimethylformamide (DMF) (40 mL), isopropyl alcohol (IPA) (40 mL), lithium perchlorate ($LiClO_4$) (2.5 mmol - 265 mg), and ammonium acetate (NH_4OAc) (20 mmol – 1.54 g) were added. A constant current of 3.0 mA at 60 °C was used for 10 hours. After the reaction is completed, the current was turned off, the electrodes were removed, and 100 mL of methanol along with 20 mL of a saturated aqueous solution of sodium bisulfite were added. The mixture was stirred for an additional 100 minutes. It was then extracted with a 10% ethyl acetate solution in hexane (4 × 100 mL) and dried over $MgSO_4$. Finally, the solvent was removed under reduced pressure using a high-vacuum pump, yielding a reddish-brown crystalline solid. Purification was carried out by column chromatography using a 10% ethyl acetate in hexane solution as the eluent, to afford product **2a** as a white solid (560 mg, 2.76 mmol, 57% yield).

Figure S4. Reaction setup of the scaling up of **2a**.

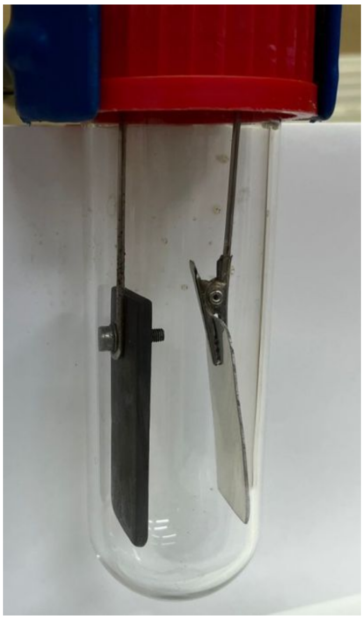

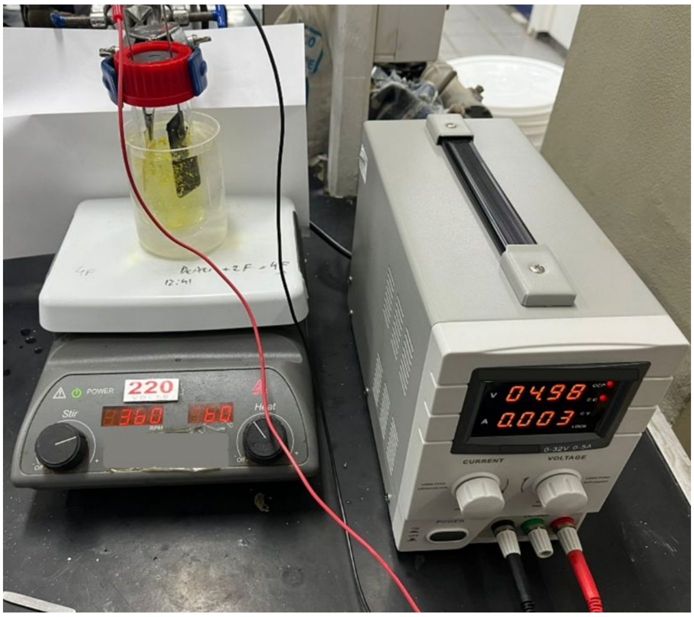

4. GENERAL PROCEDURE FOR CYCLIC VOLTAMMETRY

Cyclic voltammetry experiments were performed in a conventional, undivided three-electrode electrochemical cell using a GAMRY® potentiostat/galvanostat, model 1010E, at the Núcleo Integrado de Polímeros (NIPOL), Department of Chemistry – UDESC. The cell was equipped with a coiled platinum wire as the counter electrode, a glassy carbon disk electrode (3.0 mm diameter) as the working electrode, and a Ag/Ag^+ reference electrode with a silver wire.

Electrochemical solutions were prepared in an anhydrous mixture of N,N-dimethylformamide (DMF) and isopropanol (IPA), containing lithium perchlorate ($LiClO_4$) as the supporting electrolyte at a concentration of 0.1 $mol \cdot L^{-1}$. All measurements were conducted at 25 °C.

To investigate the reaction mechanism, the compounds 2-(phenylethynyl)benzaldehyde (**1a**) and 3-phenylisoquinoline (**2a**) were analyzed separately at a concentration of 2.0×10^{-3} $mol \cdot L^{-1}$. Potential scans were performed in the range of –1.8 to +1.4 V, with a scan rate of 100 $mV \cdot s^{-1}$. Potential values were corrected against the normal hydrogen electrode (NHE) using the ferrocene/ferrocenium (Fc/Fc^+) redox couple as an internal standard.

Figure S5. Cyclic voltammogram of **1a** and **2a**.

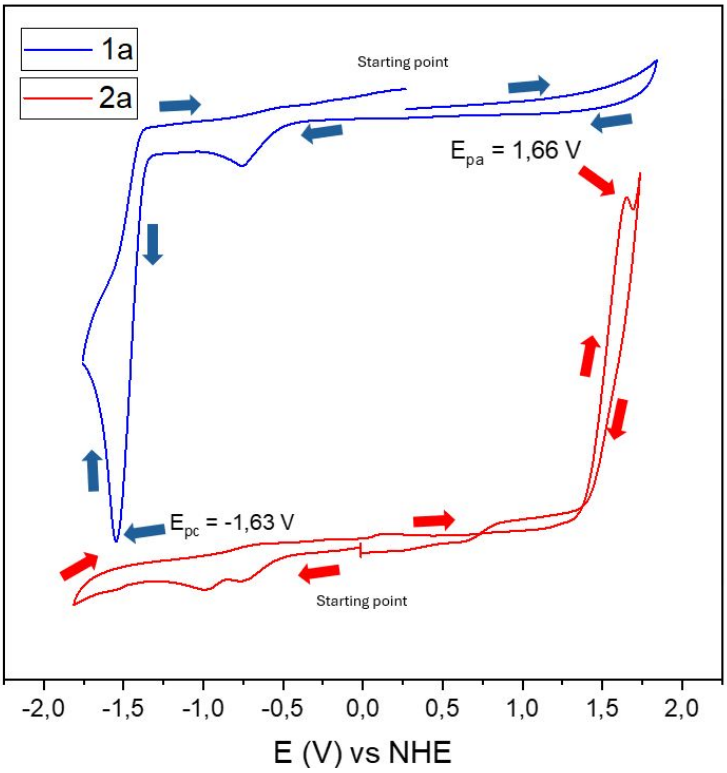

5. CONTROL EXPERIMENTS

Figure S6. Control experiments.a (A) Reaction with TEMPO (B) Reaction no-current with silver-salt (C) Reaction with silver-salt and current (D) Reaction divided-cell. *Standard conditions: **1a** (0.25 mmol), $LiClO_4$ (0.25 mmol, 1 eq.), NH_4OAc (1 mmol, 4 eq) in IPA:DMF (1:1, 0.06M), constant current = 3 mA, at 60 °C under air for 30 min. Isolated yield.

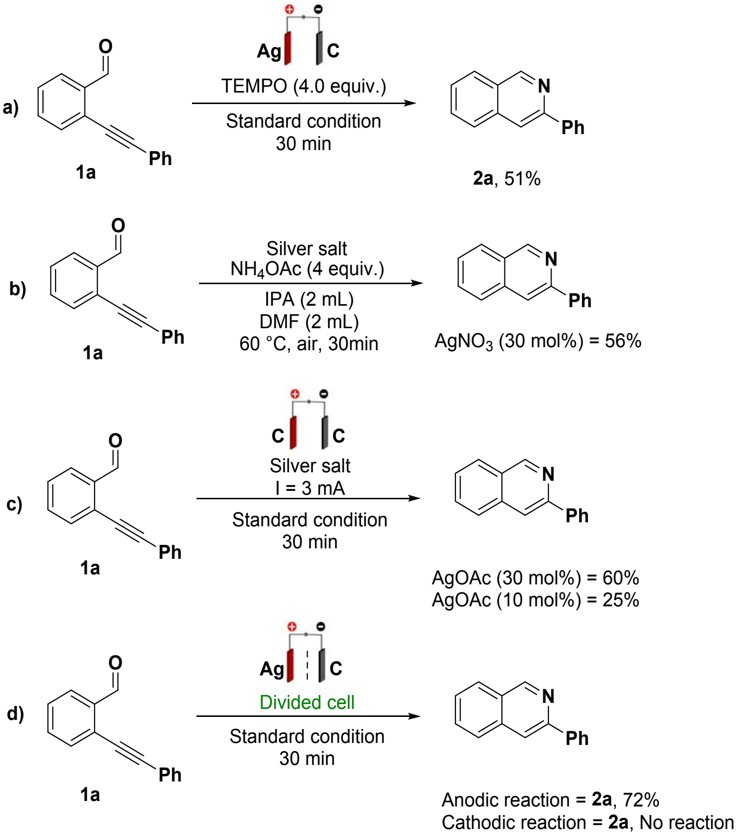

Figure S7. Divided-cell setup.

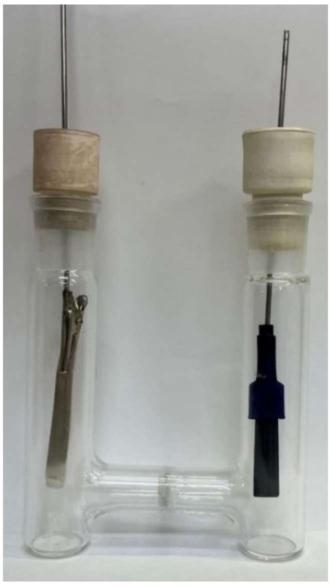

Table S1. ON/OFF experiments.

Current	Reaction time (min)	Product 2a (%)	Starting material 1a (%)
ON	0	0	100
OFF	0	0	100
ON	3	36	63
OFF	6	36	63
ON	9	41	59
OFF	12	42	58
ON	15	47	53
OFF	18	47	53
ON	21	52	48
OFF	24	53	47
ON	27	57	43
OFF	30	57	43
ON	33	61	39
OFF	36	62	38
ON	39	65	35
OFF	42	67	33
ON	45	75	25
OFF	48	76	24
ON	51	79	21
OFF	54	79	21
ON	57	82	18
OFF	60	82	18

Figure S8. ON/OFF diagram.

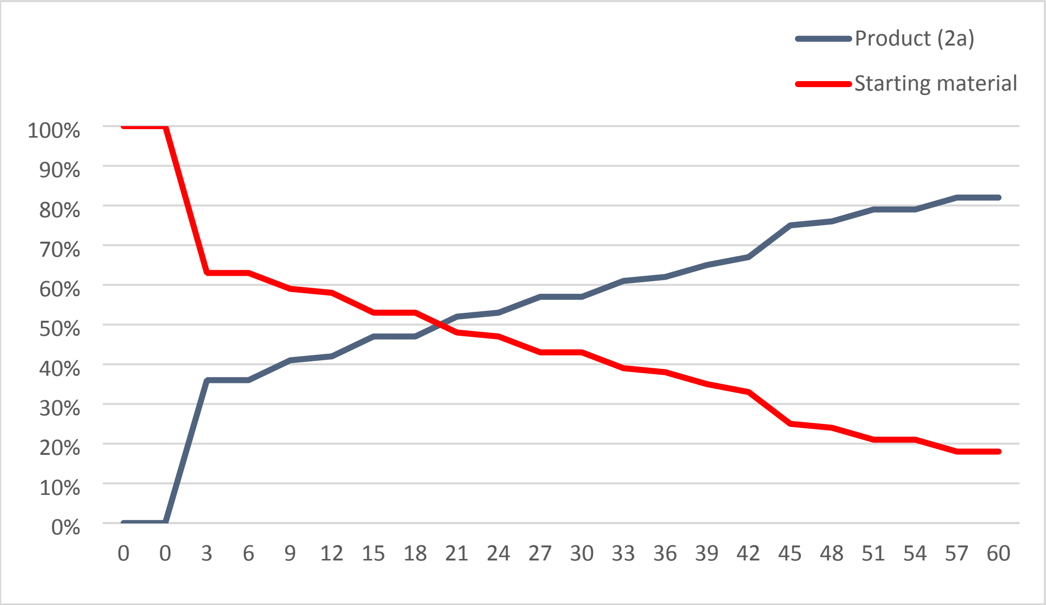

6. ELECTRIC CHARGE

The Faradays per mole (F/mol) represents the amount of electric charge required to drive an electrochemical reaction per mole of substance. This is based on Faraday's constant, which defines the charge of one mole of electrons as approximately 96.485 Coulombs. The value of F/mol provides insight into how many moles of electrons are transferred in each reaction, helping to quantify the relationship between the applied charge and the amount of material undergoing electrochemical conversion. The calculation of F/mol is crucial for understanding the efficiency and stoichiometry of electrochemical processes.[4]

To calculate, the total charge (Q) is required, which is expressed in Coulombs (C) or Ampere-seconds (A·s). This is determined by multiplying the current (I) in amperes by the time (t) in seconds. Additionally, the amount of reagent used (n), in mols, is needed. For illustration, our reactions were carried out over 30 minutes (or 1,800 seconds) with a current of 3 mA (or $3 \cdot 10^{-3}$ A), and the total charge is:

$$Q = I \times t = 3 \times 10^{-3}\, A \times 1800\, s = 5.4\, C$$

A total of 0.25 mmol or 2.5×10^{-4} mol of reagent was used. The charge per mole of reagent is:

$$\frac{Q}{n} = \frac{5{,}4\, C}{2{,}5 \times 10^{-4}} = 21600\ {}^{C}\!/_{mol}$$

Normalizing by the Faraday's constant:

$$ {}^{F}\!/_{mol} = \frac{21600\ C/mol}{F} = \frac{21600\ C/mol}{96485\ C/mol} \approx 0.22$$

7. CHARACTERIZATION DATA OF PRODUCTS

7.1 Compounds 2a-2p

3-phenylisoquinoline[5] ***2a***

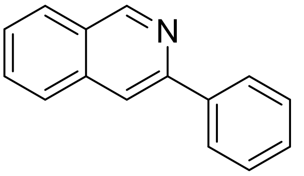

White solid, (38.9 mg, 0.19 mmol, 76% yield), m.p.: 95.1 – 96.2 °C. The reaction was purified through column chromatography on silica gel flash (elution: hexane and ethyl acetate = 90:10). ^{1}H NMR (400 MHz; $CDCl_3$): δ = 9.32 (s, 1H), 8.13 - 8.12 (m, 2H), 8.04 (s, 1H), 7.97 - 7.95 (d, *J* = 8.1 Hz, 1H), 7.85 - 7.83 (d, *J* = 8.1 Hz, 1H), 7.69 - 7.65 (m, 1H), 7.58 (m, 1H), 7.52 - 7.48 (m, 2H), 7.42 - 7.39 (m, 1H). ^{13}C{H} NMR (100 MHz; $CDCl_3$): δ = 152.4, 151.3, 139.6, 136.6, 130.5, 128.8, 128.5, 127.7, 127.5, 127.0, 127.0, 126.9, 116.5. CG-EM (*m/z*; rel. int. %): 206 (M^+; 16), 205 (100), 204 (56), 203 (10), 177 (8), 176 (13), 102 (23).

3-(4-chlorophenyl)isoquinoline[6] ***2b***

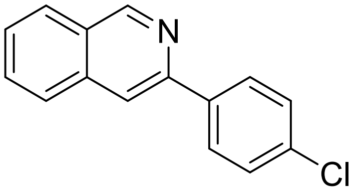

Yellow solid, (36.6 mg, 0.15 mmol, 51% yield), m.p.: 141.7 – 142.4 °C. The reaction was purified through column chromatography on silica gel flash (elution: hexane and ethyl acetate = 90:10). ^{1}H NMR (400 MHz; $CDCl_3$): δ = 9.32 (s, 1H), 8.09 - 8.06 (m, 2H), 8.04 (s, 1H), 8.00 – 7.98 (d, *J* = 8.1 Hz, 1H), 7.88 - 7.86 (d, *J* = 8.3 Hz, 1H), 7.73- 7.68 (m, 1H), 7.62 - 7.58 (m, 1H), 7.48 - 7.46 (m, 2H). ^{13}C{H} NMR (100 MHz; $CDCl_3$): δ = 152.5, 150.0, 138.0, 136.6, 134.6, 130.7, 128.9, 128.2, 127.8, 127.6, 127.3, 126.9, 116.4. CG-EM (*m/z*; rel. int. %): 241 (M^{++}; 34), 240 (M^+; 24), 239 (100), 238 (23), 204 (48), 203 (22), 176 (17), 102 (31), 88(18).

3-(4-fluorophenyl)isoquinoline[6] ***2c***

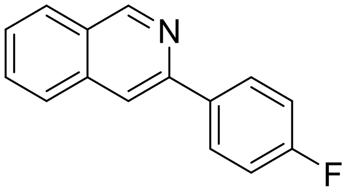

Brown solid, (40.1 mg, 0.18 mmol, 72% yield), m.p.: 120.2 – 121.4 °C. The reaction was purified through column chromatography on silica gel flash (elution: hexane and ethyl acetate = 90:10). ^{1}H NMR (400 MHz; $CDCl_3$): δ = 9.32 (s, 1H), 8.13 - 8.09 (m, 2H), 8.02 (s, 1H), 8.01 – 7.99 (d, *J* = 8.28 Hz, 1H), 7.88 – 7.86 (d, *J* = 8.09 Hz, 1H), 7.73 - 7.69 (m, 1H), 7.61 - 7.58 (m, 1H), 7.26 - 7.17 (m, 2H). ^{13}C{H} NMR (100 MHz; $CDCl_3$): δ = 163.4 (d, $J^1_{C(Ar)\text{-}F}$ = 248.1 Hz), 151.4, 149.3, 135.6, 134.7 (d, $J^4_{C(Ar)\text{-}F}$ = 3.1 Hz), 129.6, 127.7 (d, $J^3_{C(Ar)\text{-}F}$ = 8.1 Hz), 127.6, 126.6, 126.5,

126.1, 115.1, 114.7 (d, J^2 $_{C(Ar)-F}$ = 21.4 Hz). CG-EM (*m/z*; rel. int. %): 224 (m^+; 17), 223 (100), 222 (60), 221 (7), 194 (7), 175 (5), 111 (14), 101 (8), 97 (5).

3-(p-tolyl)isoquinoline[7] ***2d***

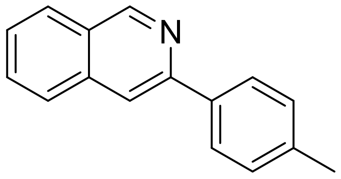

Pale yellow solid, (36.6 mg, 0.16 mmol, 67% yield), m.p.: 68.9 – 69.8 °C. The reaction was purified through column chromatography on silica gel flash (elution: hexane and ethyl acetate = 90:10). ^{1}H NMR (400 MHz, $CDCl_3$): δ = 9.32 (s, 1H), 8.03 - 8.01 (m, 3H), 7.97 (dd, *J* = 9.22 Hz, 1.01 Hz, 1H), 7.83 (dd, *J* = 9.11 Hz, 0.86 Hz 1H), 7.68 - 7.64 (m, 1H), 7.57 - 7.55 (m, 1H), 7.32 – 7.30 (d, *J* = 8.0 Hz, 2H), 2.42 (s, 3H). ^{13}C{H} NMR (100 MHz; $CDCl_3$): δ = 152.3, 151.3, 138.4, 136.8, 136.7, 130.4, 129.5, 127.6, 127.5, 126.8, 116.0, 21.2. CG-EM (*m/z*; rel. int. %): 219 (100), 220 (17) 218 (50), 217 (20), 108 (18).

3-(2-methoxyphenyl)isoquinoline[8] ***2e***

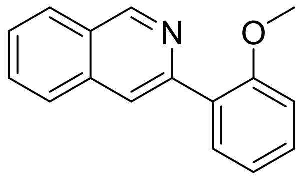

Orange solid, (38.7 mg, 0.16 mmol, 66% yield), m.p.: 67.7 – 68.7 °C. The reaction was purified through column chromatography on silica gel flash (elution: hexane and ethyl acetate = 90:10). ^{1}H NMR (400 MHz; $CDCl_3$): δ = 9.33 (s, 1H) 8.20 (s, 1H), 7.97 - 7.96 (d, 1H), 7.92 - 7.90 (d, 1H), 7.84 - 7.82 (d, 1H), 7.67 - 7.63 (m, 1H), 7.57 - 7.53 (m, 1H), 7.37 - 7.35 (m, 1H), 7.13 - 7.09 (m, 1H), 7.04 - 7.02 (d, 1H), 3.88 (s, 3H). ^{13}C{H} NMR (100 MHz; $CDCl_3$): δ = 157.1, 151.9, 149.3, 136.2, 131.4, 130.2, 129.5, 129.2, 127.4, 127.0, 126.9, 126.9, 121.1, 121.0, 111.5, 55.7. CG-EM (*m/z*; rel. int. %): 236 (m^+; 11), 235 (71), 234 (100), 218 (13), 207 (18), 206 (47), 205 (44), 204 (67), 165 (11), 130 (47), 102 (44).

7-methoxy-3-phenylisoquinoline[6] ***2f***

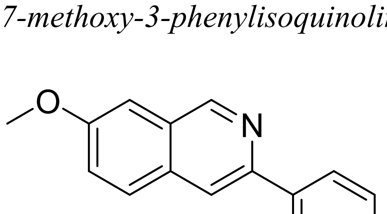

Yellow solid, (29.9 mg, 0.12 mmol, 63% yield, 2 hours), m.p.: 144.8 – 145.4 °C. The reaction was purified through column chromatography on silica gel flash (elution: hexane and ethyl acetate = 90:10). ^{1}H NMR (400 MHz; $CDCl_3$): δ = 9.24 (s, 1H), 8.10 - 8.08 (m, 2H), 8.00 (s, 1H), 7.79 - 7.76 (d, *J* = 9.0 Hz, 1H), 7.51 - 7.47 (m, 2H), 7.41 - 7.37 (m, 1H), 7.36 - 7.33 (m, 1H), 7.24 (d, *J* = 2.43 Hz, 1H), 3.96 (s, 3H). ^{13}C{H} NMR (100 MHz; $CDCl_3$): δ = 158.4, 150.9, 149.7, 139.7, 132.3,

128.9, 128.7, 128.4, 128.1, 126.7, 123.7, 116.4, 104.7, 55.5. CG-EM (*m/z*; rel. int. %): 236 (M^+; 19), 235 (100), 220 (35), 192 (43), 191 (14), 190 (8), 165 (28), 164 (8), 117 (9), 95 (8).

6-fluoro-3-phenylisoquinoline[6] ***2g***

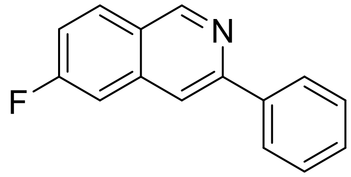

White solid, (33.4 mg, 0.15 mmol, 60% yield), m.p.: 123.6 – 124.8 °C. The reaction was purified through column chromatography on silica gel flash (elution: hexane and ethyl acetate = 90:10). ^{1}H NMR (400 MHz, $CDCl_3$): δ = 9.30 (s, 1H), 8.12 – 8.10 (d, *J* = 7.7 Hz, 2H), 8.02 – 7.99 (m, 2H), 7.54 – 7.43 (m, 4H), 7.37 – 7.34 (m, 1H). ^{13}C{H} NMR (100 MHz; $CDCl_3$): δ = 164.7 (d, $J^1_{C(Ar)\text{-}F}$ = 252.8 Hz), 152.1 152.0, 139.2, 138.2 (d, $J^3_{C(Ar)\text{-}F}$ = 10.1 Hz), 130.7 (d, $J^3_{C(Ar)\text{-}F}$ = 9.7 Hz), 128.8, 127.0, 125.0, 117.7 (d, $J^2_{C(Ar)\text{-}F}$ = 25.8 Hz), 116.0 (d, $J^4_{C(Ar)\text{-}F}$ = 5.4 Hz), 110.3 (d, $J^4_{C(Ar)\text{-}F}$ = 21.0 Hz). CG-EM (*m/z*; rel. int. %): 224 (M^+; 15), 223 (100), 222 (70), 221 (8), 195 (4), 194 (6), 175 (4), 111 (15), 101 (5), 97 (5).

7-methoxy-3-(p-tolyl)isoquinoline[9] ***2h***

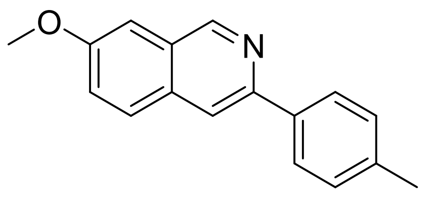

White solid, (40.4 mg, 0.16 mmol, 65% yield), m.p.: 140.8 – 141.3 °C. The reaction was purified through column chromatography on silica gel flash (elution: hexane and ethyl acetate = 90:10). ^{1}H NMR (400 MHz; $CDCl_3$): δ = 9.21 (s, 1H), 8.00 - 7.96 (m, 3H), 7.75 - 7.73 (d, *J* = 8.98 Hz, 1H), 7.34 - 7.29 (m, 3H), 7.21 (d, *J* = 2.45 Hz, 1H), 3.95 (s, 3H), 2.41 (s, 3H). ^{13}C{H} NMR (100 MHz; $CDCl_3$): δ = 158.2, 150.8, 149.7, 138.0, 136.9, 132.3, 129.5, 128.7, 128.4, 126.6, 123.7, 115.9, 104.7, 55.5, 21.2. CG-EM *m/z*: 250 (M^+; 19), 249 (100), 235 (7), 234 (37), 206 (29), 205 (10), 204 (9), 191 (7), 124 (8), 102 (8).

3-(4-chlorophenyl)-7-methoxyisoquinoline[10] ***2i***

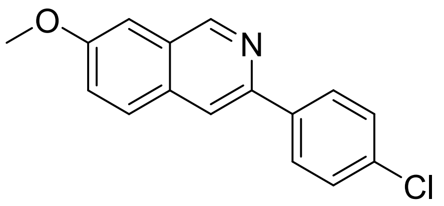

White solid, (41.0 mg, 0.15 mmol, 61% yield), m.p.: 148.7 – 149.2 °C. The reaction was purified through column chromatography on silica gel flash (elution: hexane and ethyl acetate = 90:10). ^{1}H NMR (400 MHz, $CDCl_3$): δ = 9.21 (s, 1H), 8.05 – 8.03 (m, 2H), 7.97 (s, 1H), 7.77 (d, *J* = 8.96 Hz, 1H), 7.47 - 7.44 (m, 2H), 7.37 (dd, *J* = 8.96, 6.44 Hz, 1H), 7.24 (d, *J* = 2.37 Hz, 1H), 3.97

(s, 3H). $^{13}C\{H\}$ NMR (100 MHz; $CDCl_3$): δ = 158.6, 151.0, 148.4, 138.2, 134.2, 132.2, 129.0, 128.9, 128.4, 127.9, 123.9, 116.3, 104.7, 100.0, 55.5. CG-EM (*m/z*; rel. int. %): 271 (M^{++}; 32), 270 (M^{+}; 18), 269 (100), 256 (11), 254 (35), 228 (10), 226 (31), 191 (37), 190 (13), 164 (15).

3-(4-chlorophenyl)-6-fluoroisoquinoline ***2j***

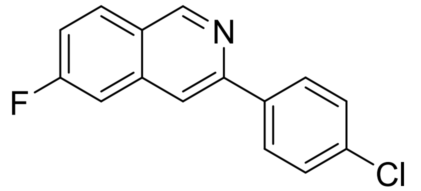

Brown solid, (39.9 mg, 0.15 mmol, 62% yield, 2 hours), m.p.: 108.7 – 110.3 °C. The reaction was purified through column chromatography on silica gel flash (elution: hexane and ethyl acetate = 90:10). ^{1}H NMR (400 MHz, $CDCl_3$): δ = 9.28 (s, 1H), 8.07 – 8.05 (m, 2H), 8.02 – 7.99 (m, 2H), 7.49 – 7.45 (m, 3H), 7.38 – 7.34 (td, *J* = 8.7, 2.5 Hz, 1H). $^{13}C\{H\}$ NMR (100 MHz; $CDCl_3$): δ = 164.8 (d, J^{1} $_{C(Ar)\text{-}F}$ = 253.1 Hz), 152.1 150.9, 138.1 (d, J^{3} $_{C(Ar)\text{-}F}$ = 10.9 Hz), 137.6, 134.9, 130.6 (d, J^{3} $_{C(Ar)\text{-}F}$ = 10.0 Hz), 129.0, 128.3, 125.0, 118.0 (d, J^{2} $_{C(Ar)\text{-}F}$ = 25.7 Hz), 115.9 (d, J^{4} $_{C(Ar)\text{-}F}$ = 5.3 Hz), 110.3 (d, J^{4} $_{C(Ar)\text{-}F}$ = 21.1 Hz). CG-EM (*m/z*; rel. int. %): 259 (M^{++}; 32), 258 (M^{+}, 23), 257 (100), 256 (22), 223 (6), 222 (43), 221 (18), 194 (7), 111 (24), 97 (11). HRMS (ESI(+)-TOF) *m/z*: $[M+H]^{+}$ Calcd for $C_{15}H_9ClFN^{+}$ 258,0480; Found 258,0483.

7-phenyl-1,6-naphthyridine[11] ***2n***

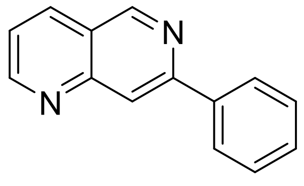

Orange solid, (29.8 mg, 0.14 mmol, 58% yield), m.p.: 133.5 °C – 134.3 °C. The reaction was purified through column chromatography on silica gel flash (elution: hexane and ethyl acetate = 90:10). ^{1}H NMR (400 MHz, $CDCl_3$): δ 9.35 (s, 1H), 9.10 (dd, *J* = 4.5, 1.7 Hz, 1H), 8.35 (s, 1H), 8.31 – 8.29 (dd, *J* = 8.2, 1.7 Hz, 1H), 8.19 – 8.17 (m, 2H), 7.55 – 7.44 (m, 4H). $^{13}C\{H\}$ NMR (100 MHz; $CDCl_3$): δ = 155.2, 155.0, 152.7, 151.4, 138.8, 135.6, 132.0, 129.2, 128.9, 127.2, 122.7, 122.2, 117.8. CG-EM (m/z; rel. int. %): 207 (M^{+}; 15), 206 (100), 205 (65), 179 (6), 178 (8), 151 (9), 103 (13), 102 (9), 89 (8), 76 (8).

8. NMR SPECTRA

8.1 Compounds 2a-2o

Figure S9. ^{1}H NMR (400 MHz, $CDCl_3$) of compound **2a**.

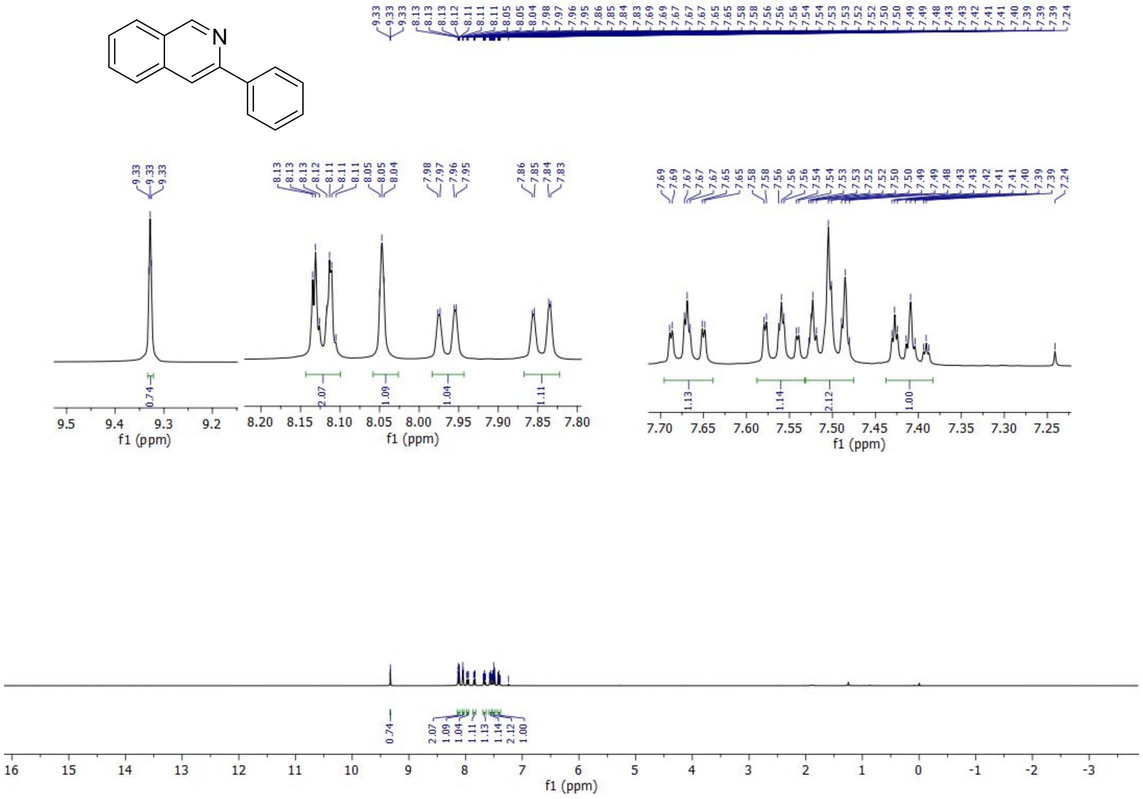

Figure S10. ^{13}C{H} NMR (100 MHz, $CDCl_3$) of compound **2a**.

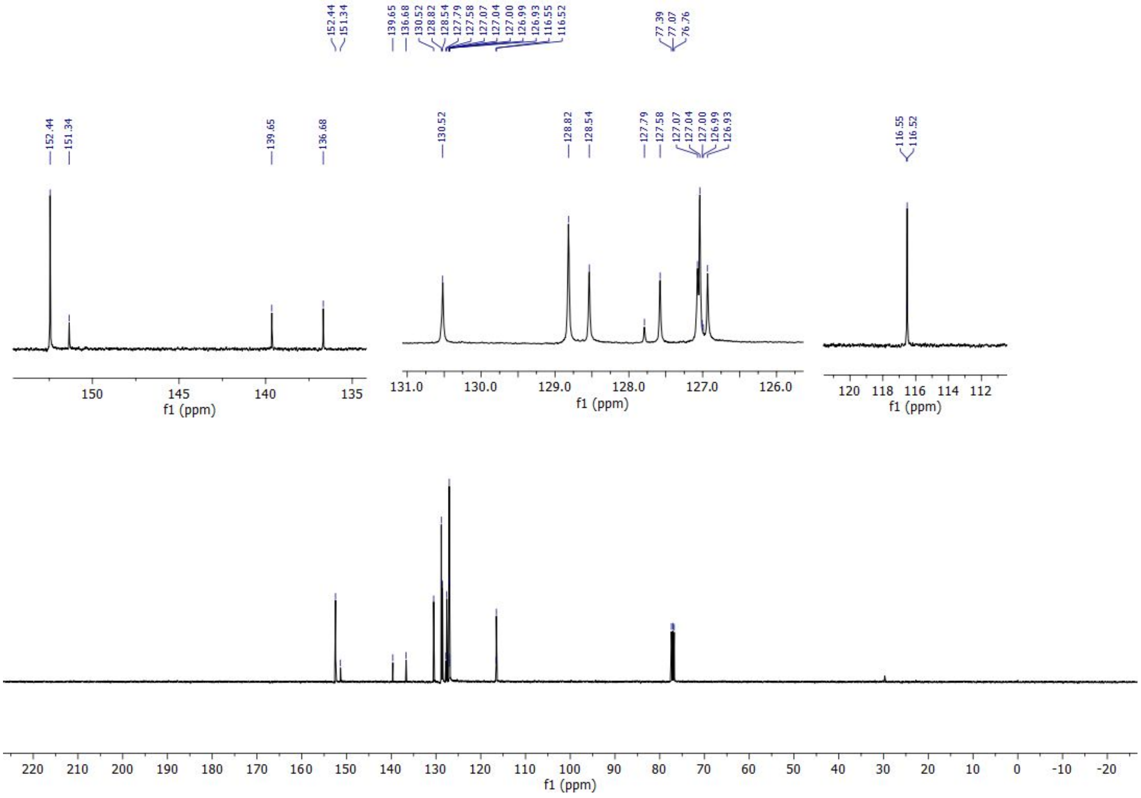

Figure S11. ^{1}H NMR (400 MHz, $CDCl_3$) of compound **2b**.

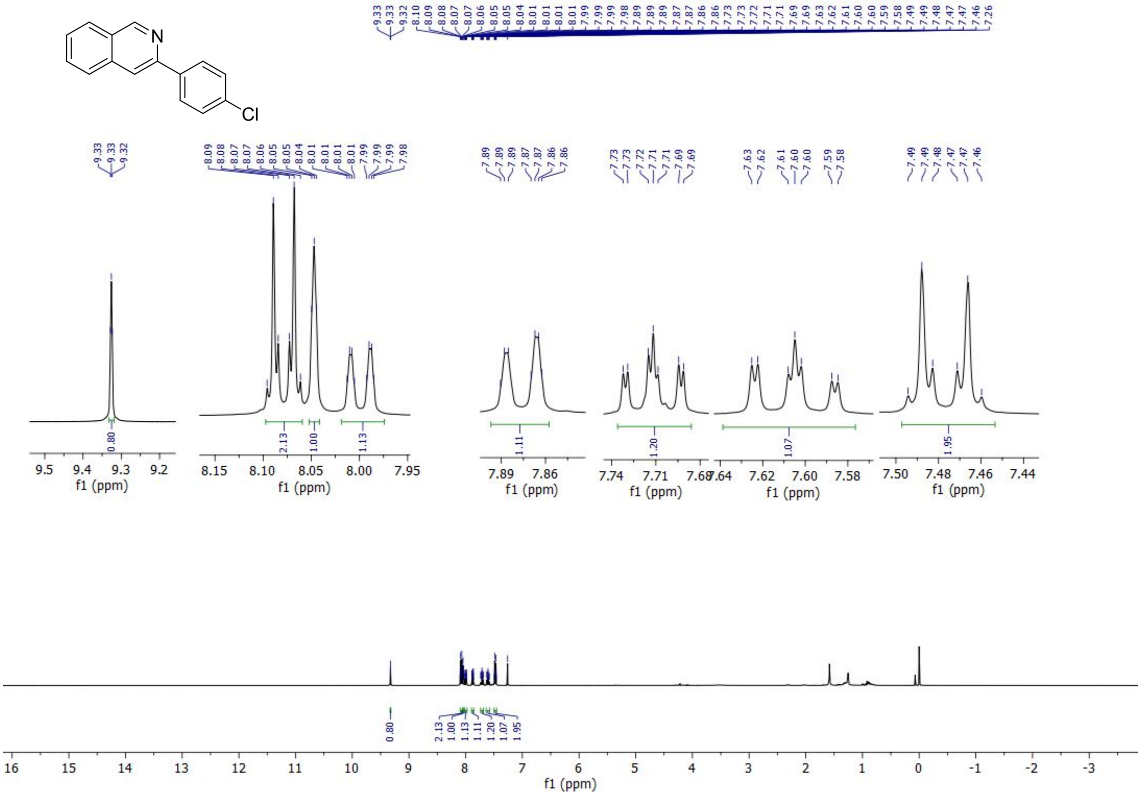

Figure S12. $^{13}C\{H\}$ NMR (100 MHz, $CDCl_3$) of compound **2b**.

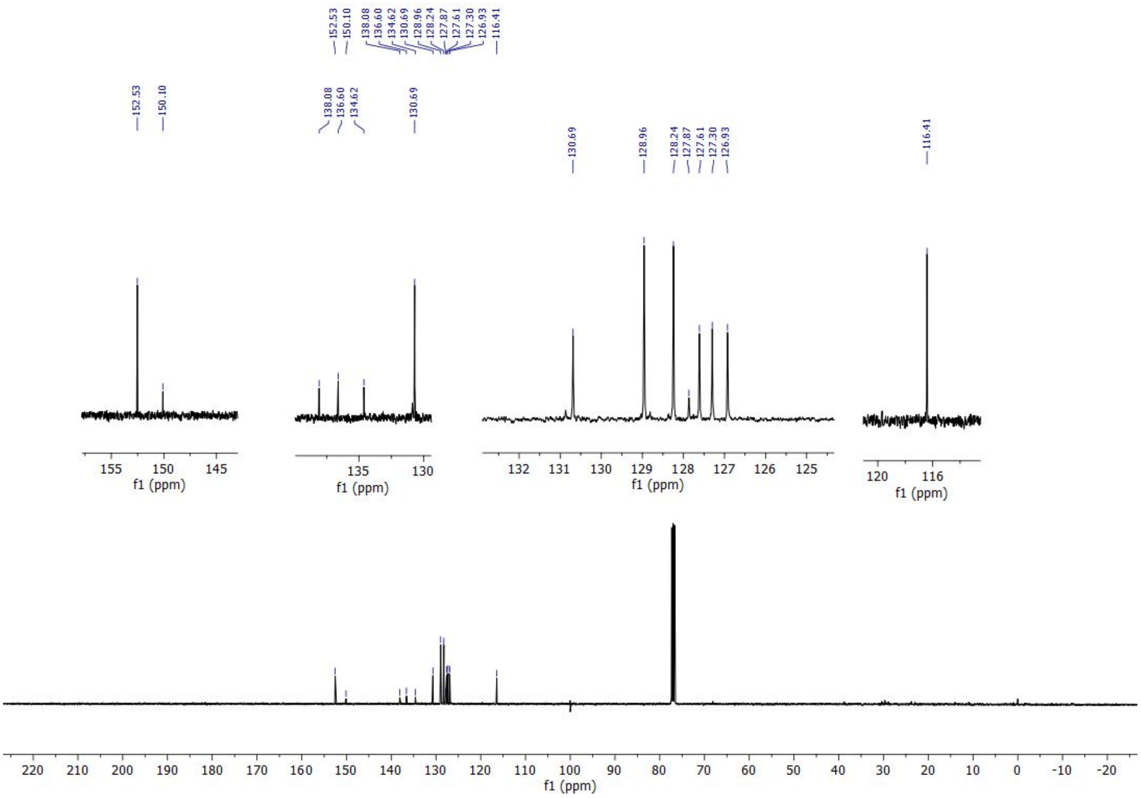

Figure S13. ^{1}H NMR (400 MHz, $CDCl_3$) of compound **2c**.

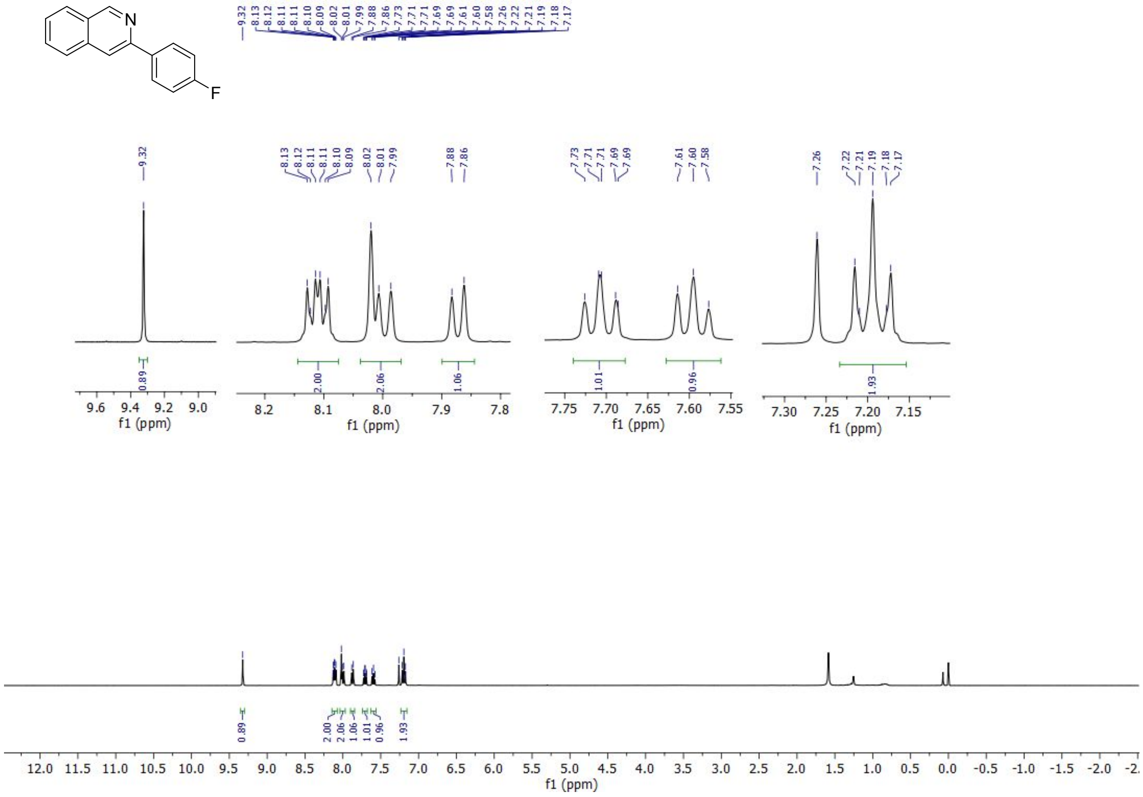

Figure S14. ^{13}C{H} NMR (100 MHz, $CDCl_3$) of compound **2c**.

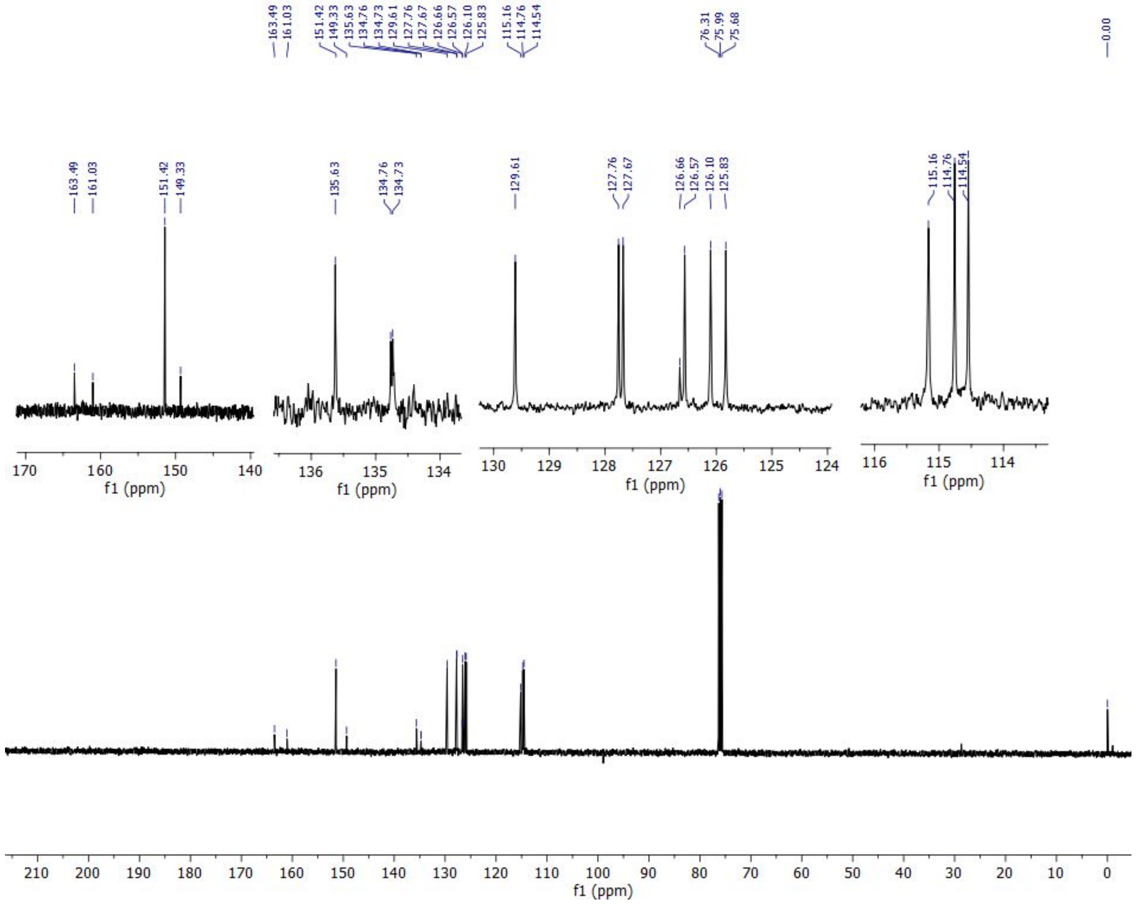

Figure S15. ^{1}H NMR (400 MHz, $CDCl_3$) of compound **2d**.

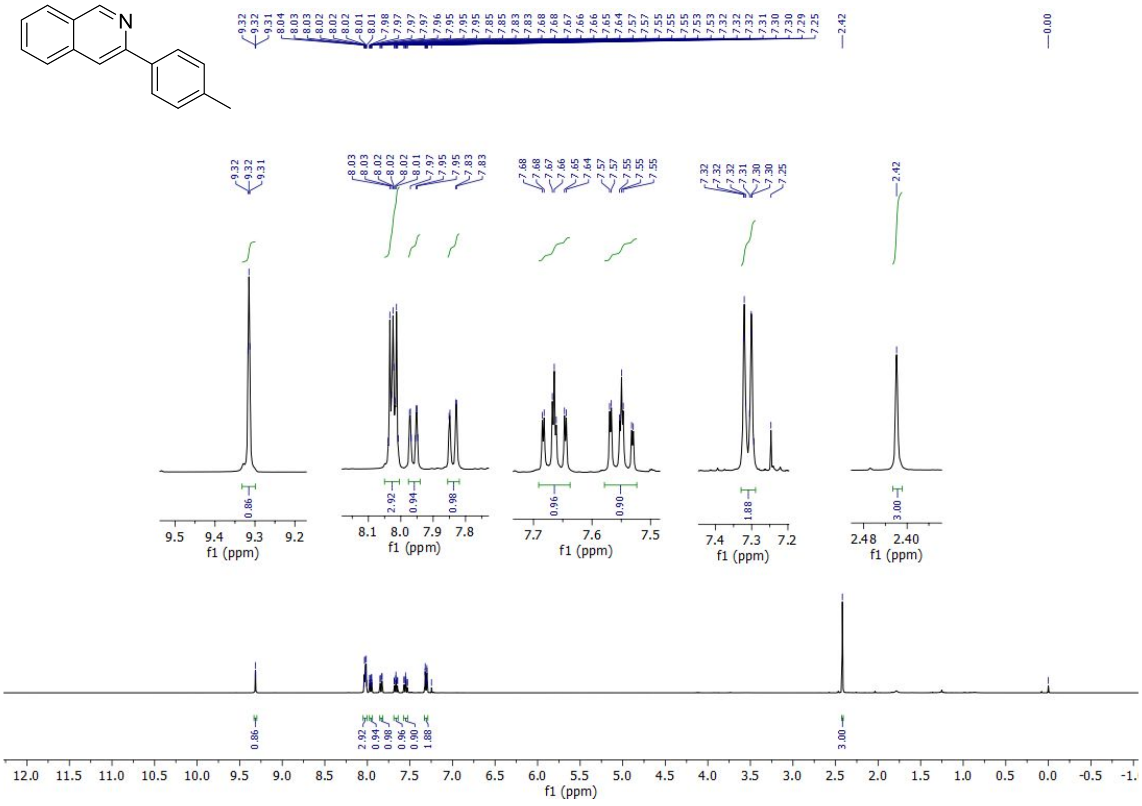

Figure S16. ^{13}C{H} NMR (100 MHz, $CDCl_3$) of compound **2d**.

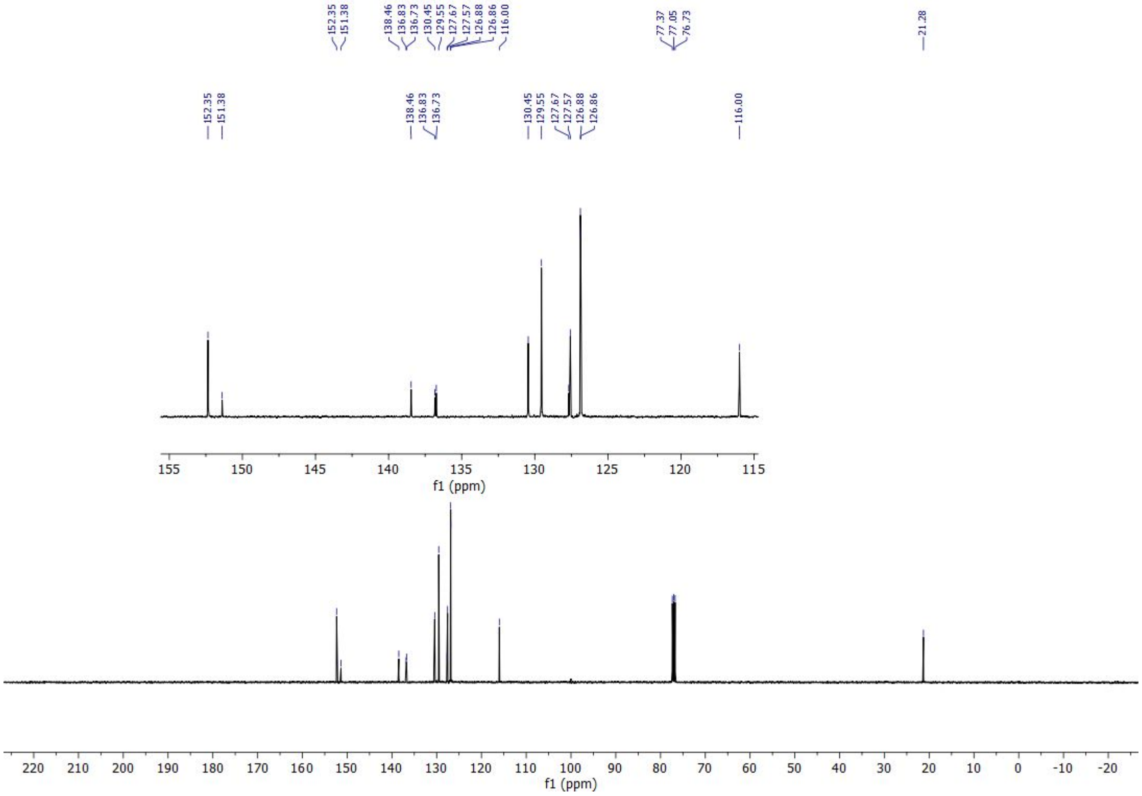

Figure S17. ^{1}H NMR (400 MHz, $CDCl_3$) of compound **2e**.

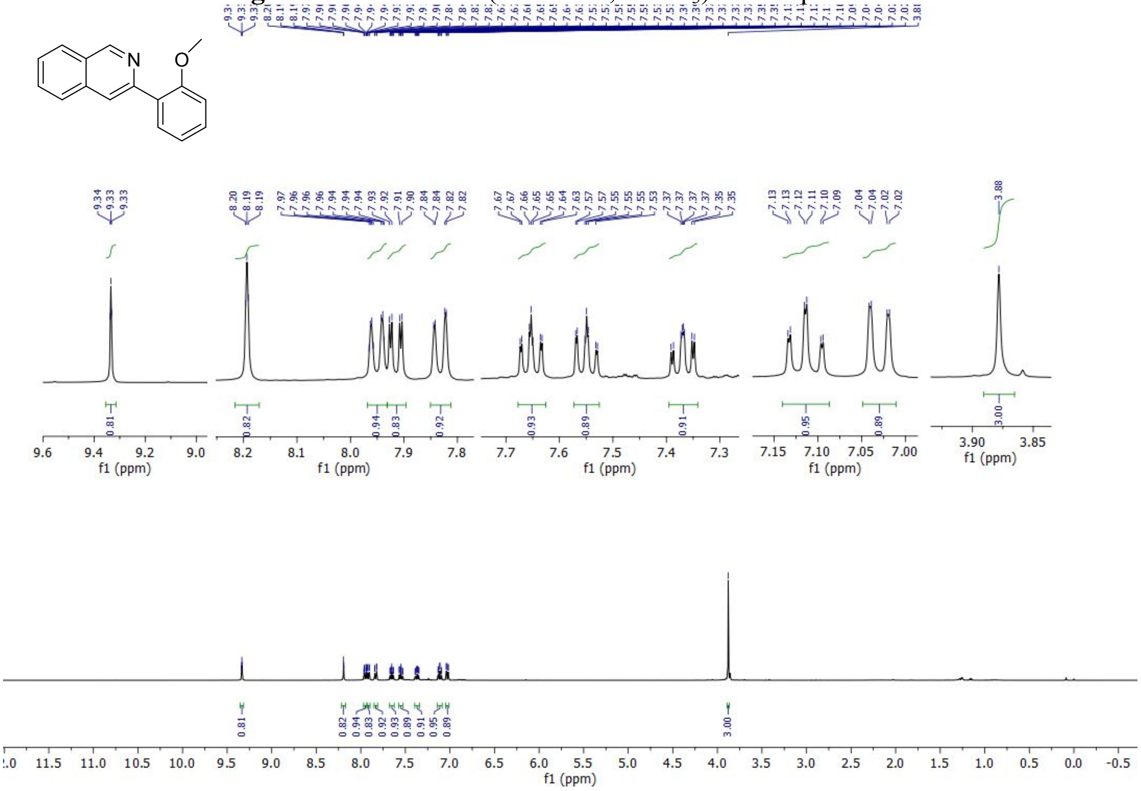

Figure S18. ^{13}C{H} NMR (100 MHz, $CDCl_3$) of compound **2e**.

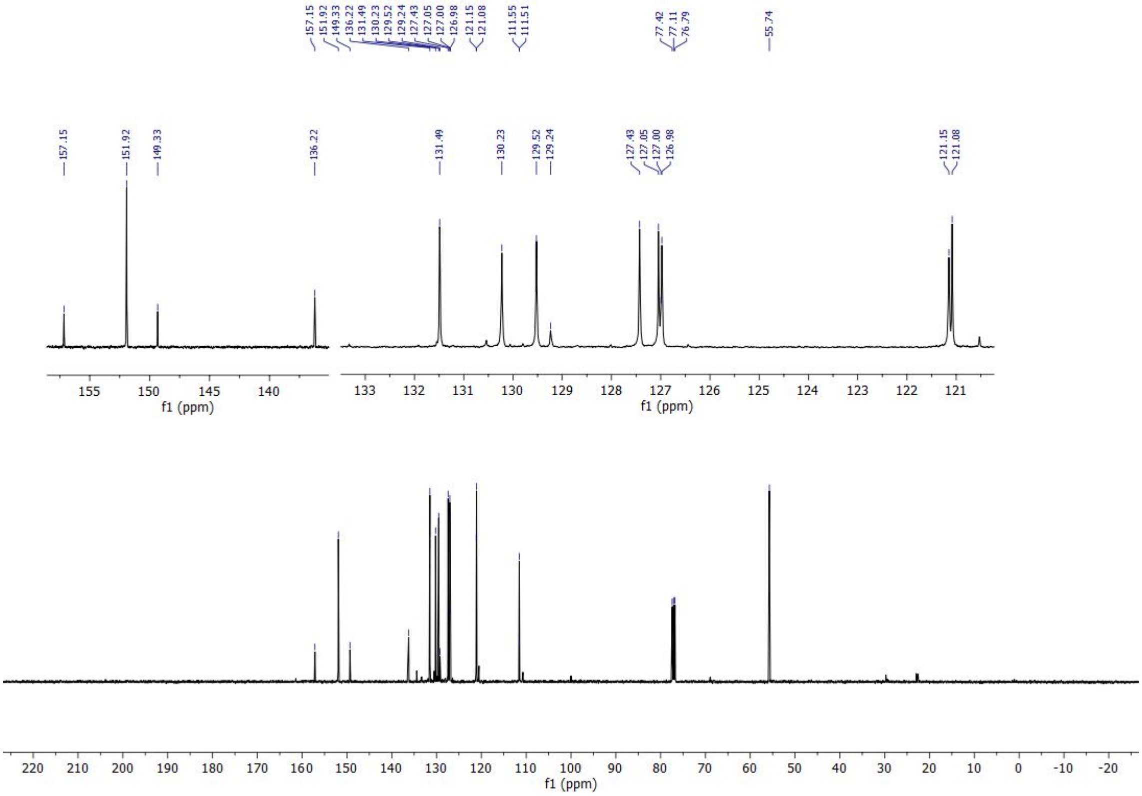

Figure S19. ^{1}H NMR (400 MHz, $CDCl_3$) of compound **2f**.

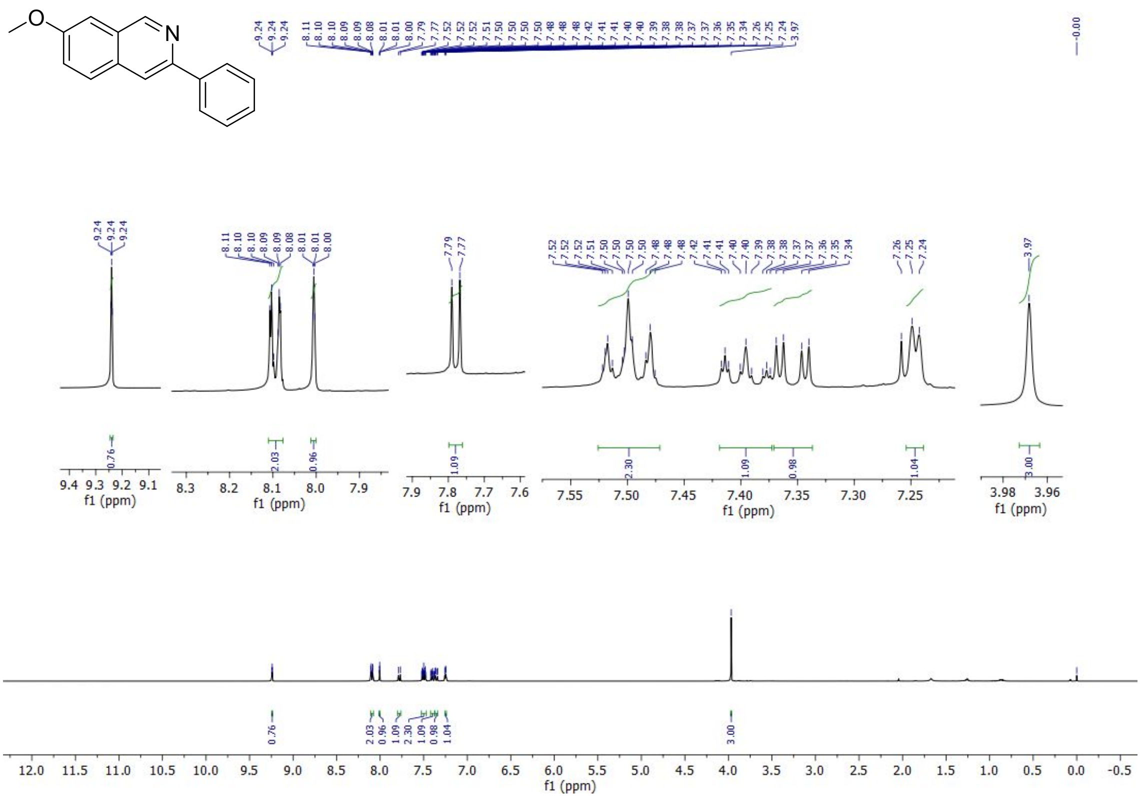

Figure S20. ^{13}C{H} NMR (100 MHz, $CDCl_3$) of compound **2f**.

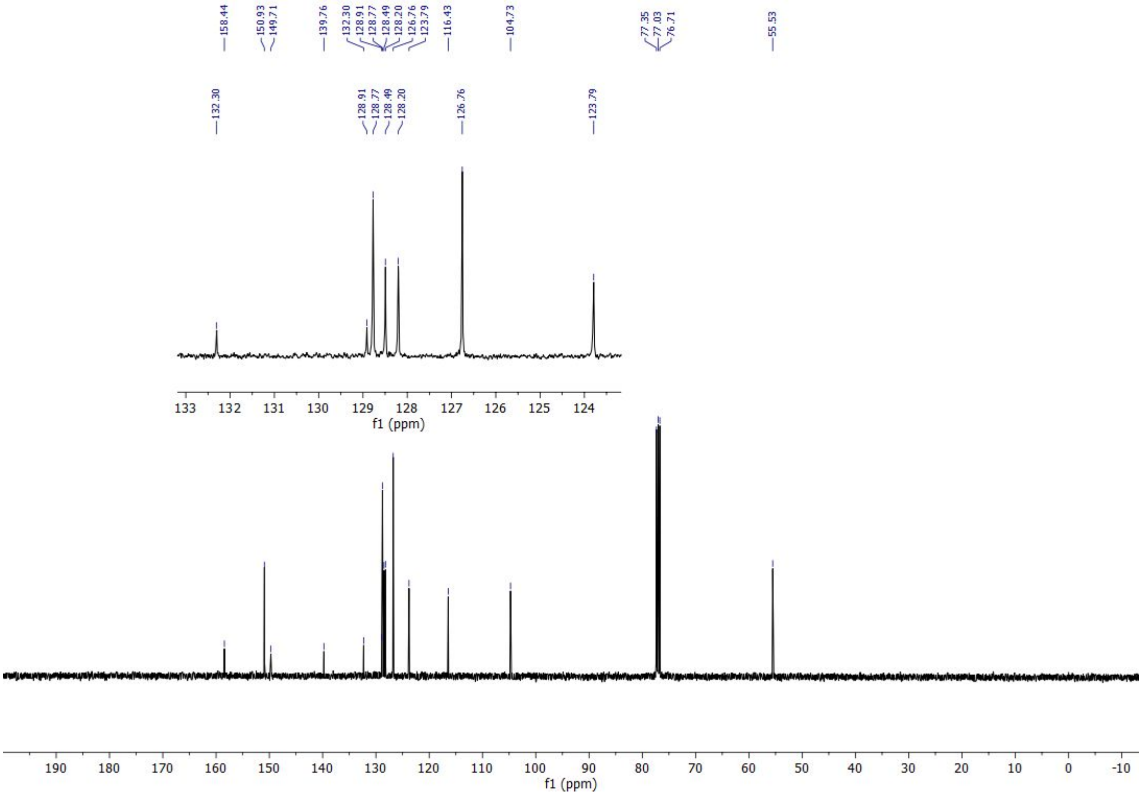

Figure S21. ^{1}H NMR (400 MHz, $CDCl_3$) of compound **2g**.

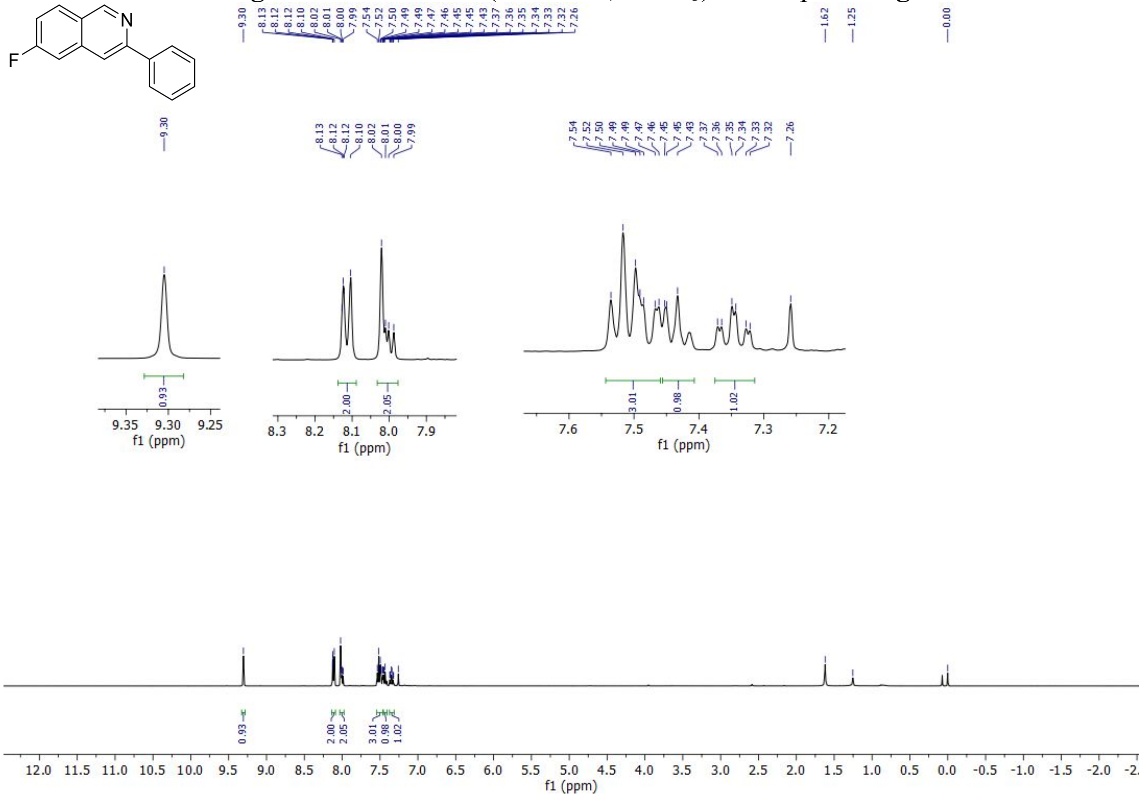

Figure S22. $^{13}C\{H\}$ NMR (100 MHz, $CDCl_3$) of compound **2g**.

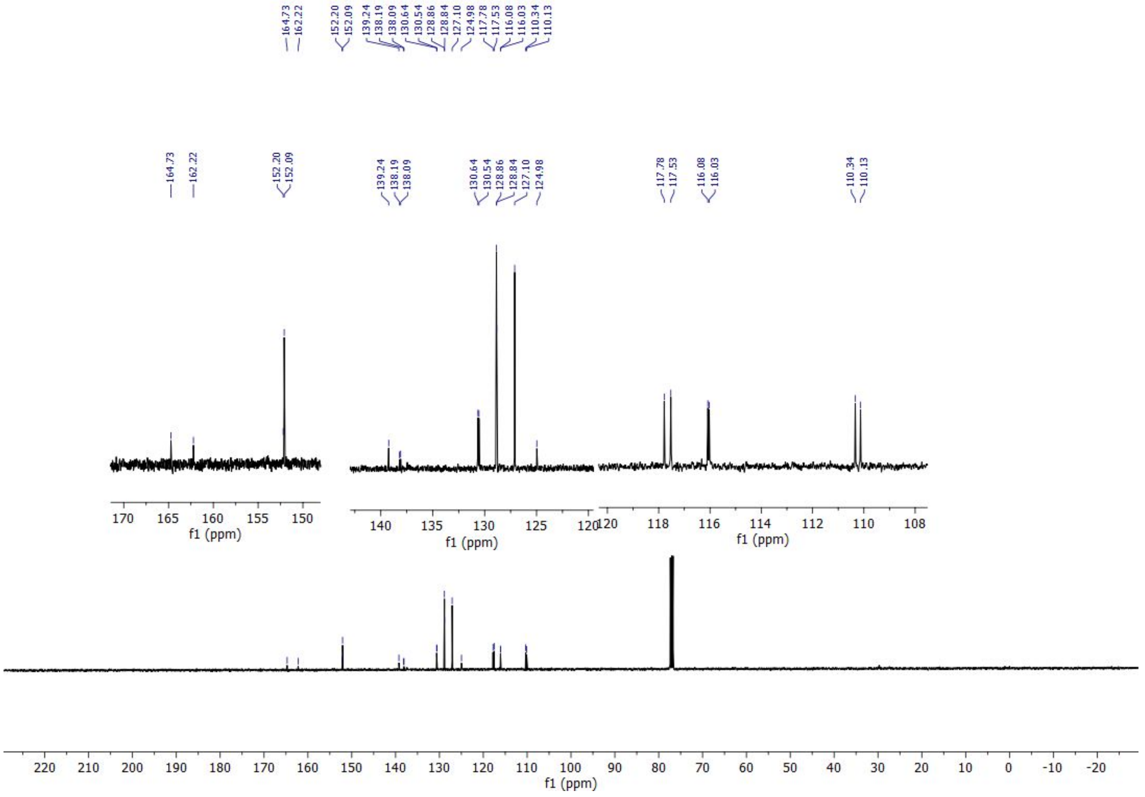

Figure S23. ^{1}H NMR (400 MHz, $CDCl_3$) of compound **2h**.

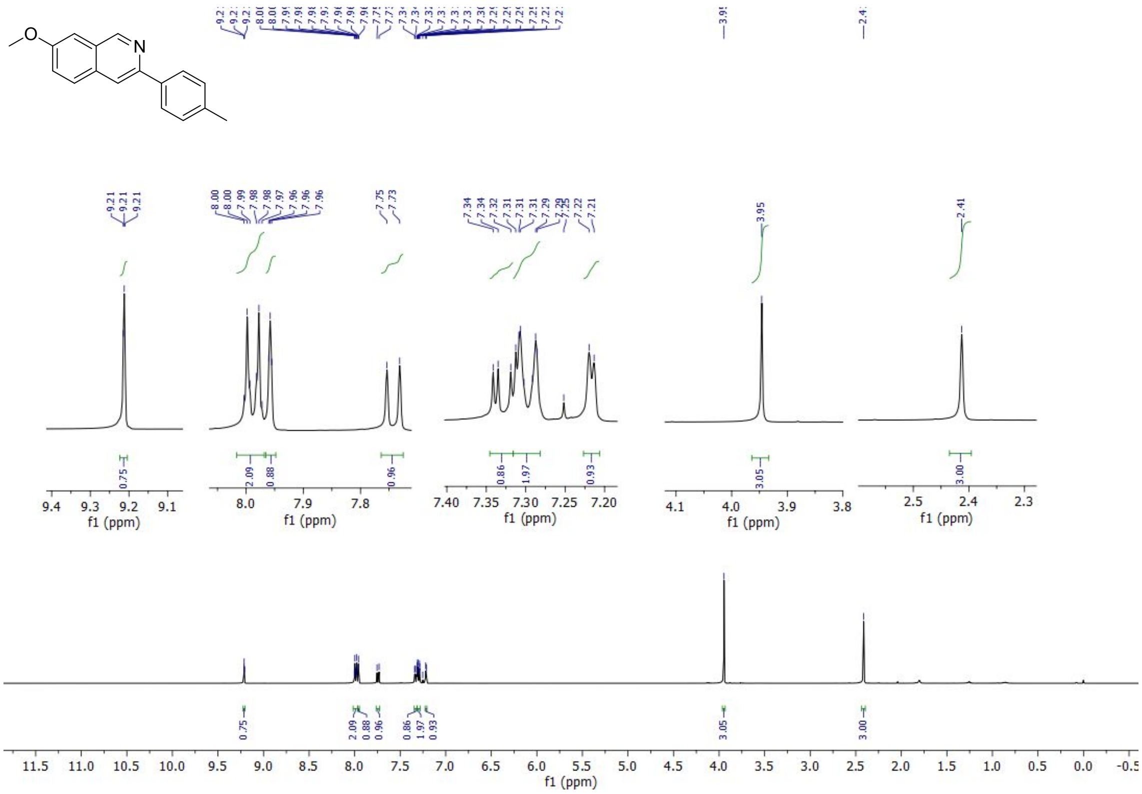

Figure S24. ^{13}C{H} NMR (100 MHz, $CDCl_3$) of compound **2h**.

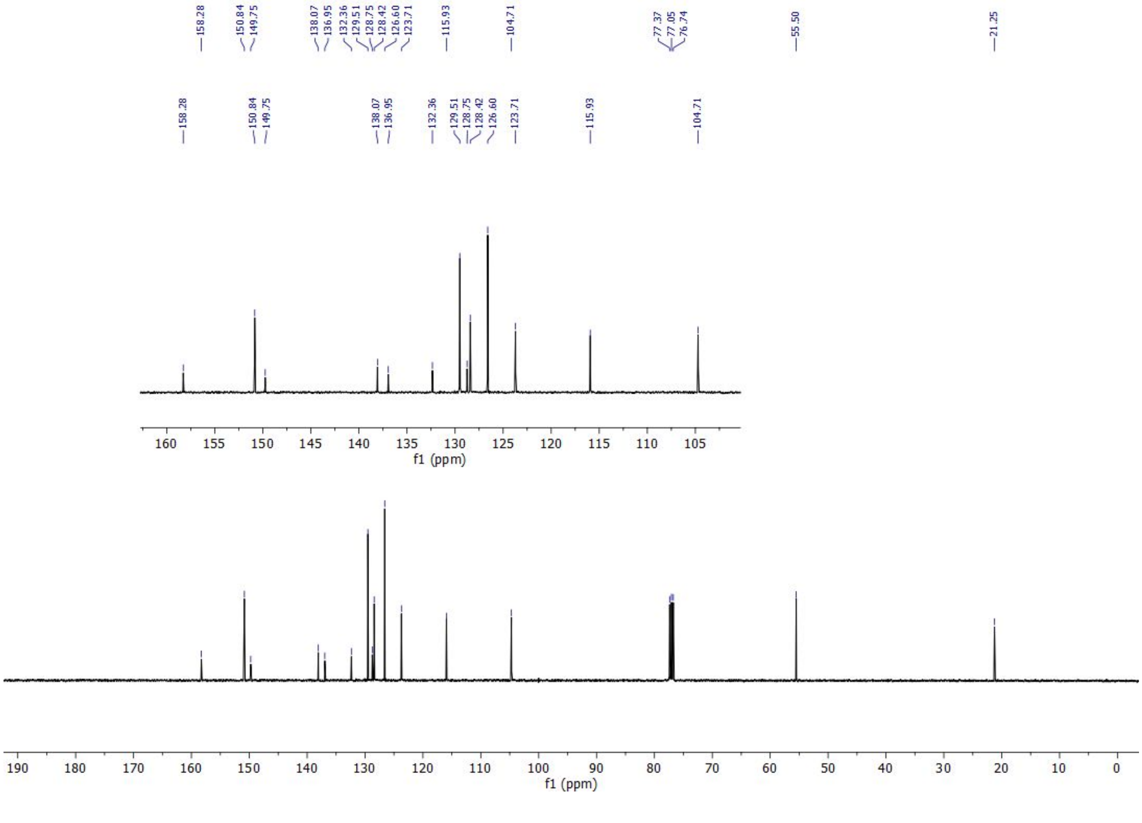

Figure S25. ^{1}H NMR (400 MHz, $CDCl_3$) of compound **2i**.

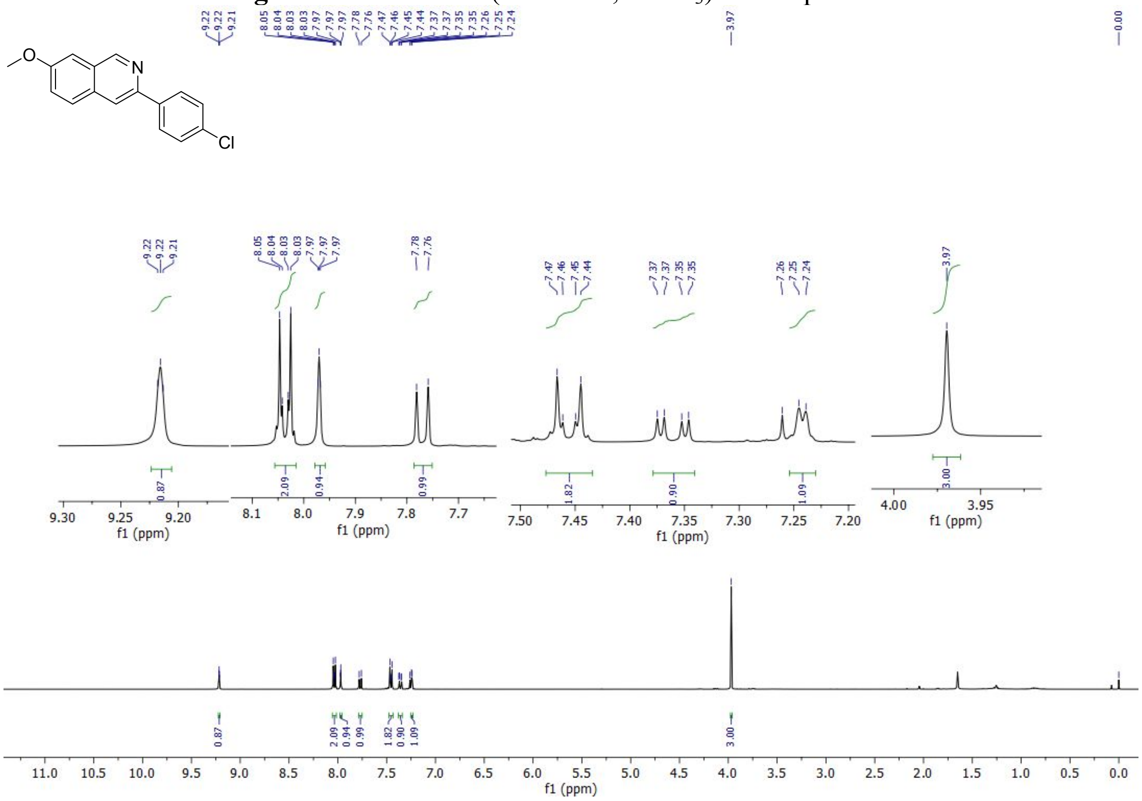

Figure S26. ^{13}C{H} NMR (100 MHz, $CDCl_3$) of compound **2i**.

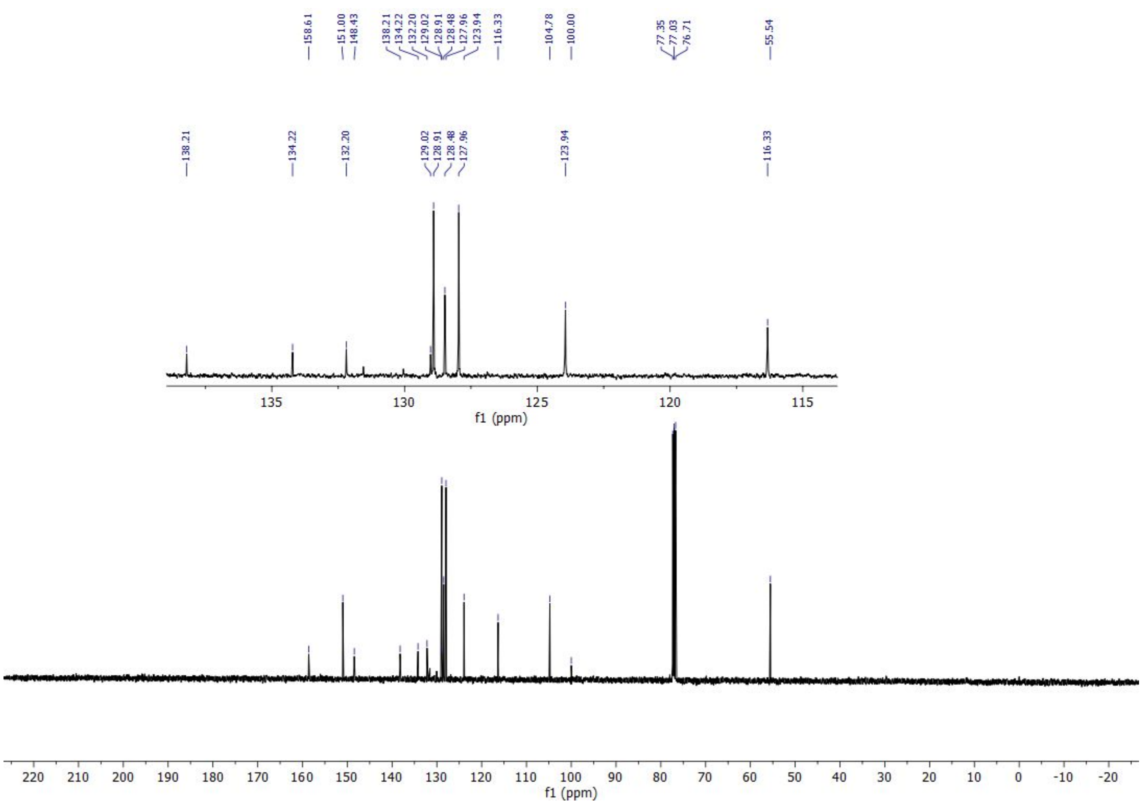

Figure S27. ^{1}H NMR (400 MHz, $CDCl_3$) of compound **2j**.

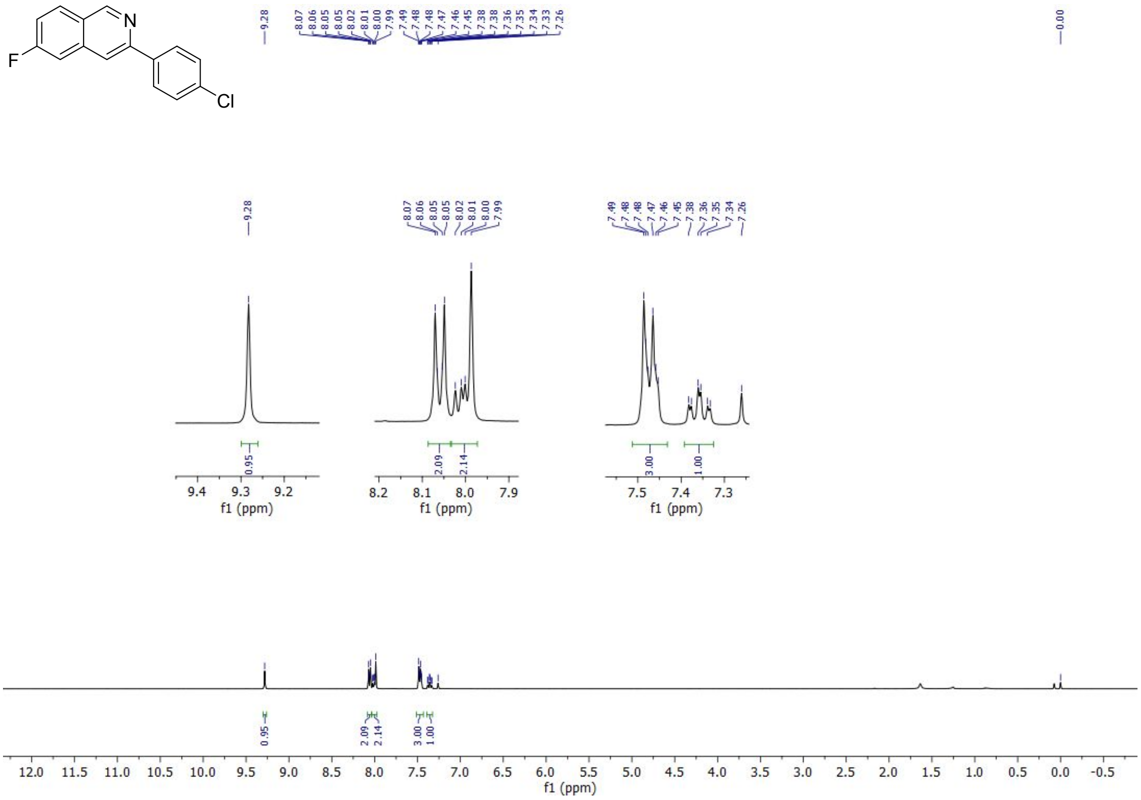

Figure S28. ^{13}C{H} NMR (100 MHz, $CDCl_3$) of compound **2j**.

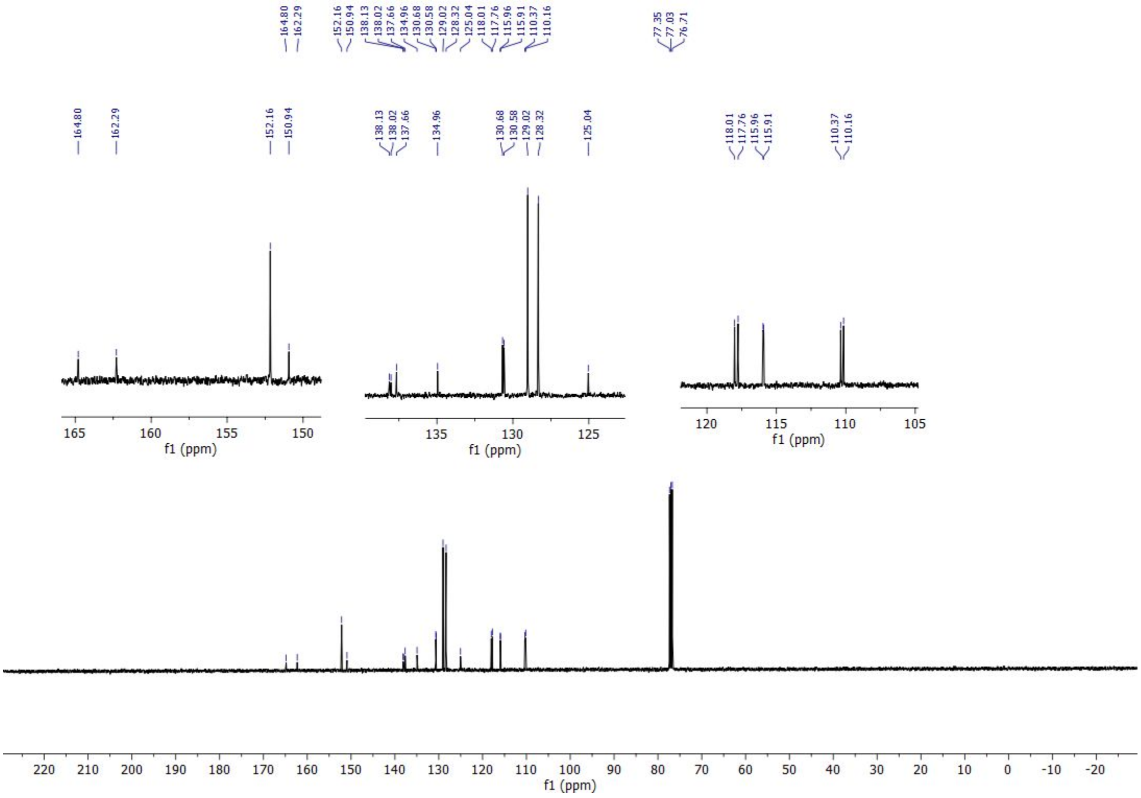

Figure S29. ^{1}H NMR (400 MHz, $CDCl_3$) of compound **2n**.

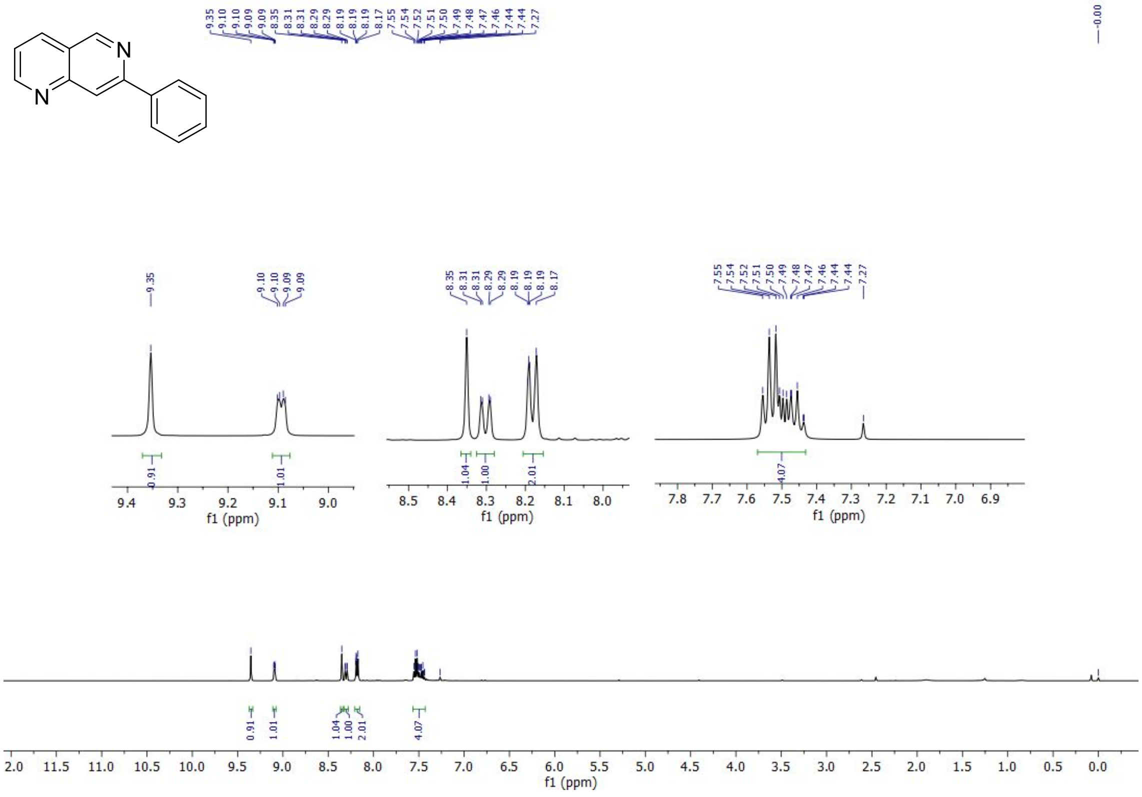

Figure S30. ^{13}C{H} NMR (100 MHz, $CDCl_3$) of compound **2n**.

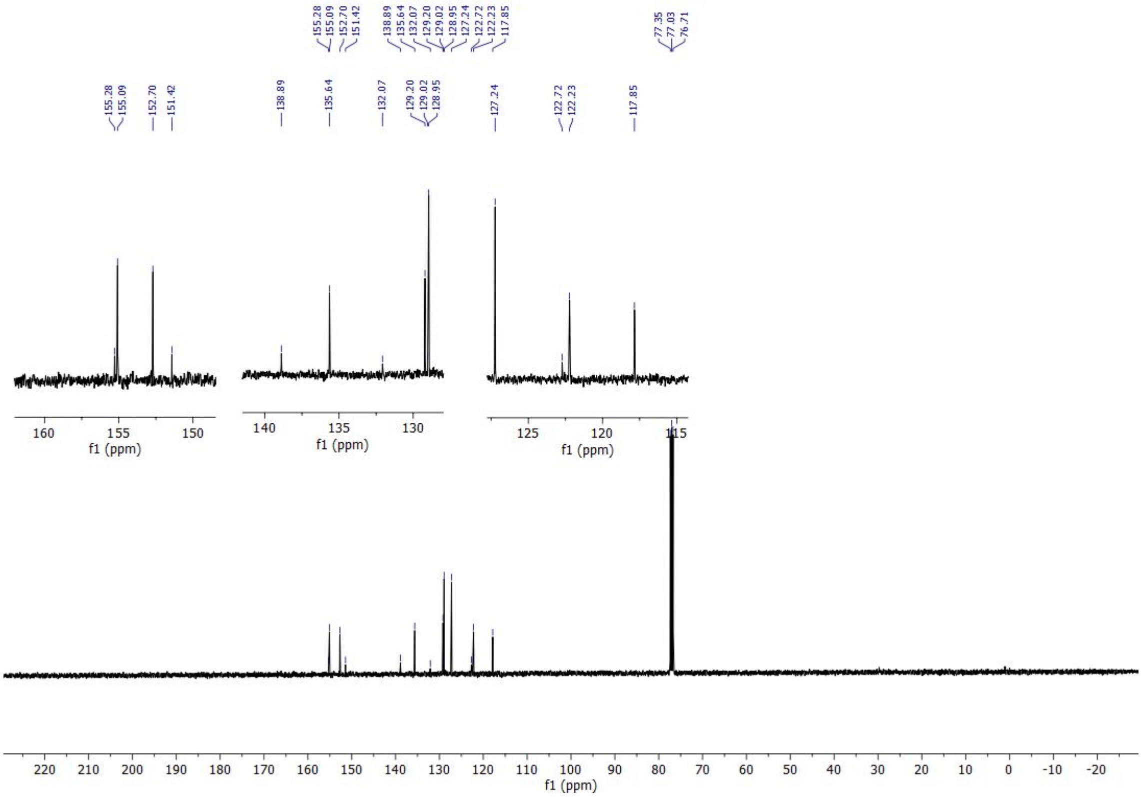

9. THEORETICAL CALCULATIONS

9.1 General overview of the evaluated mechanisms

A) Step 1

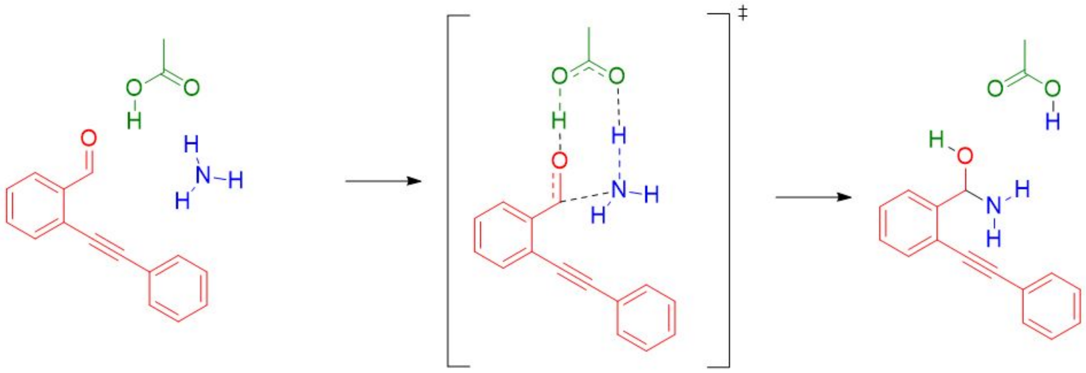

B) Step 2

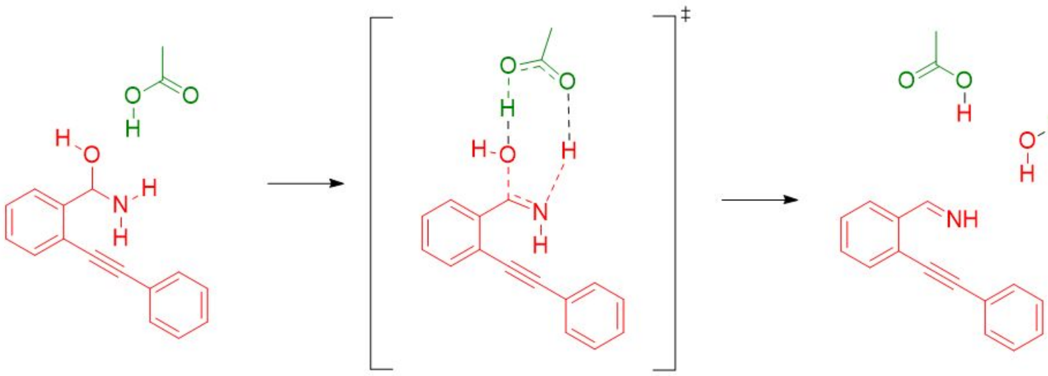

C) Step 3

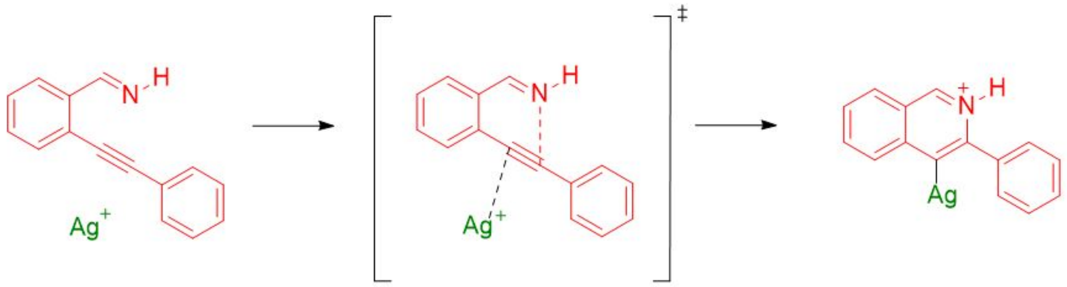

D) Step 4

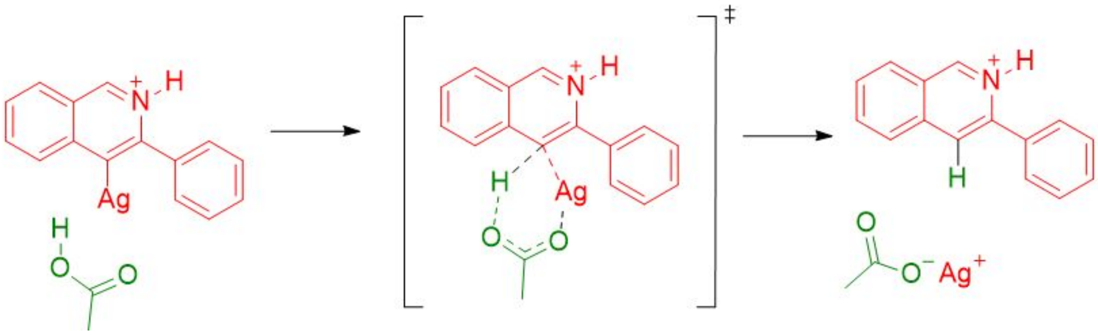

E) Step 5

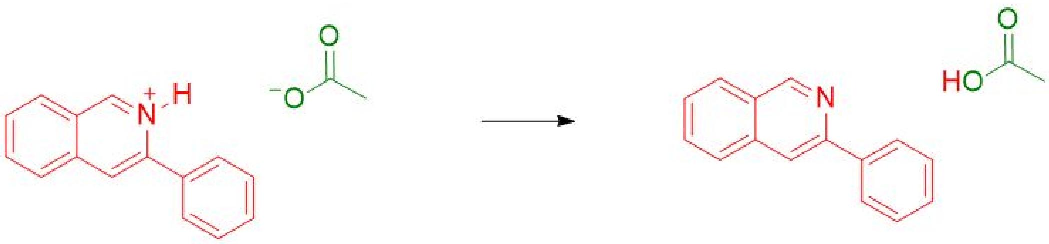

F) **Alternative *5-exo-dig* cyclization (possible according to the Baldwin's rules)**

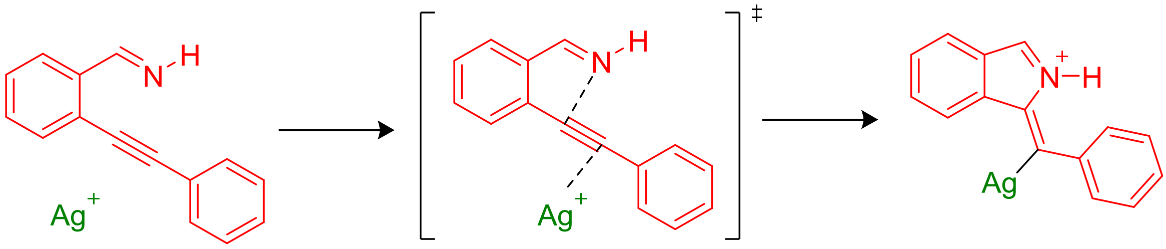

9.2 Energy profile (ΔG) of the evaluated mechanisms

Figure S31. Investigated reaction pathway.

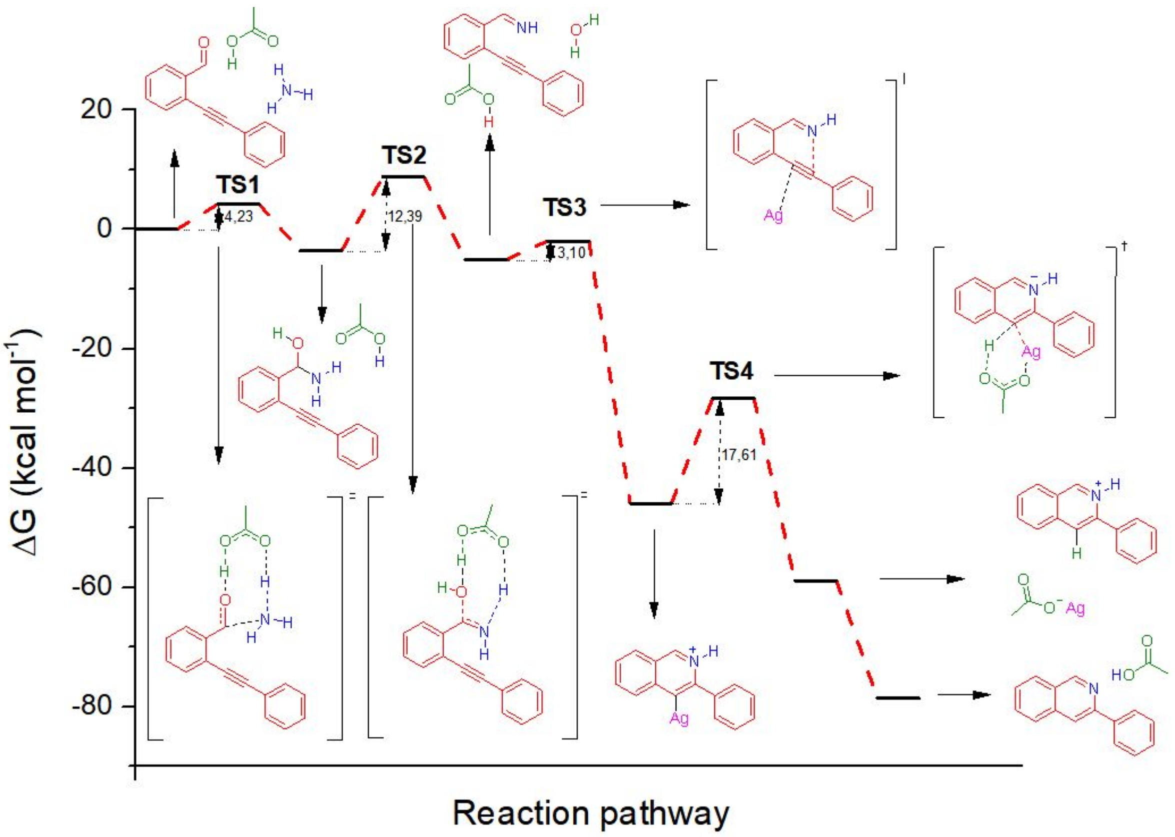

9.3 Imaginary frequencies for all transition states and intrinsic reaction coordinates

Table S2. Imaginary frequencies of all TS structures.

Transition state	Frequency (cm^{-1})
**Step 1**	-137.28
**Step 2**	-313.58
**Step 3**	-263.97
**Step 4**	-1177.51

Figure S32. Intrinsic Reaction Coordinate – Step 1

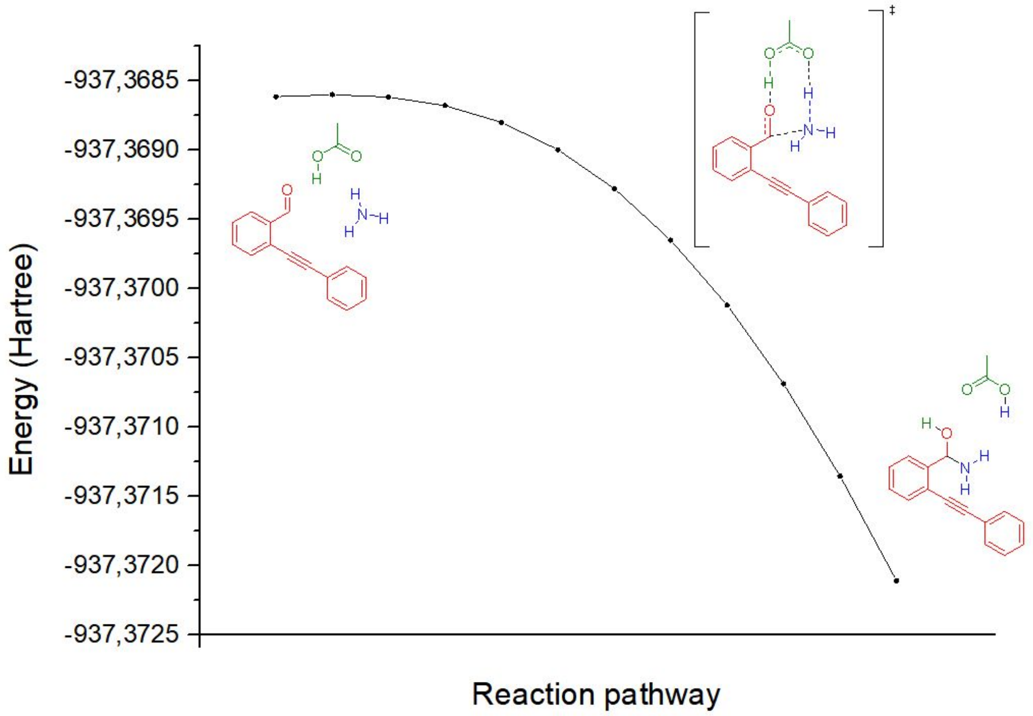

Figure S33. Intrinsic Reaction Coordinate – Step 2

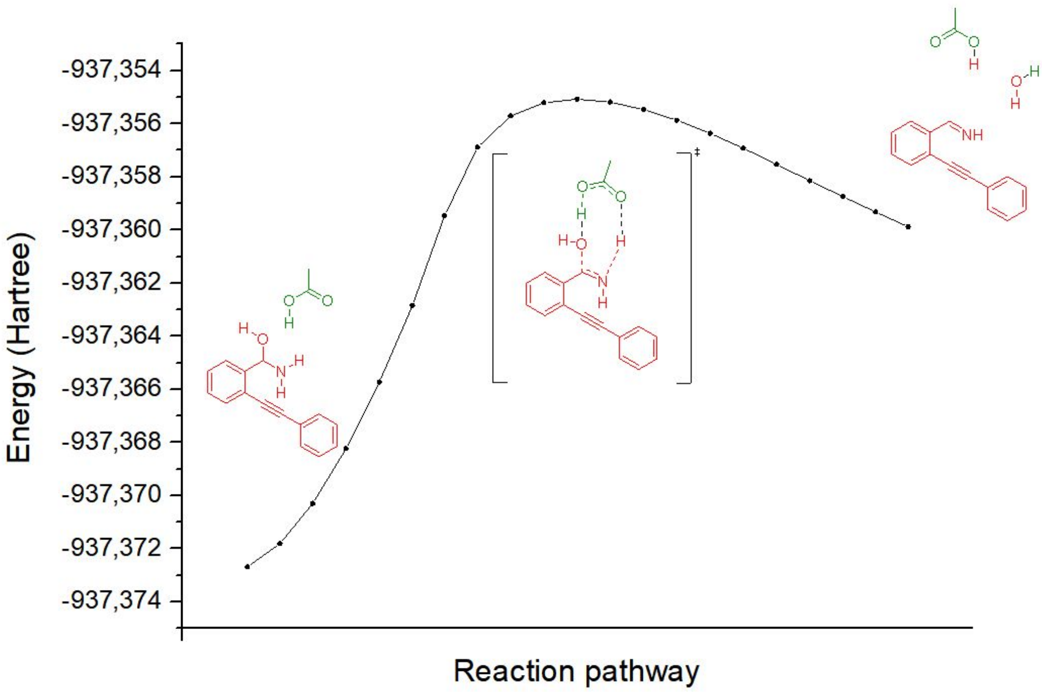

Figure S34. Intrinsic Reaction Coordinate – Step 3

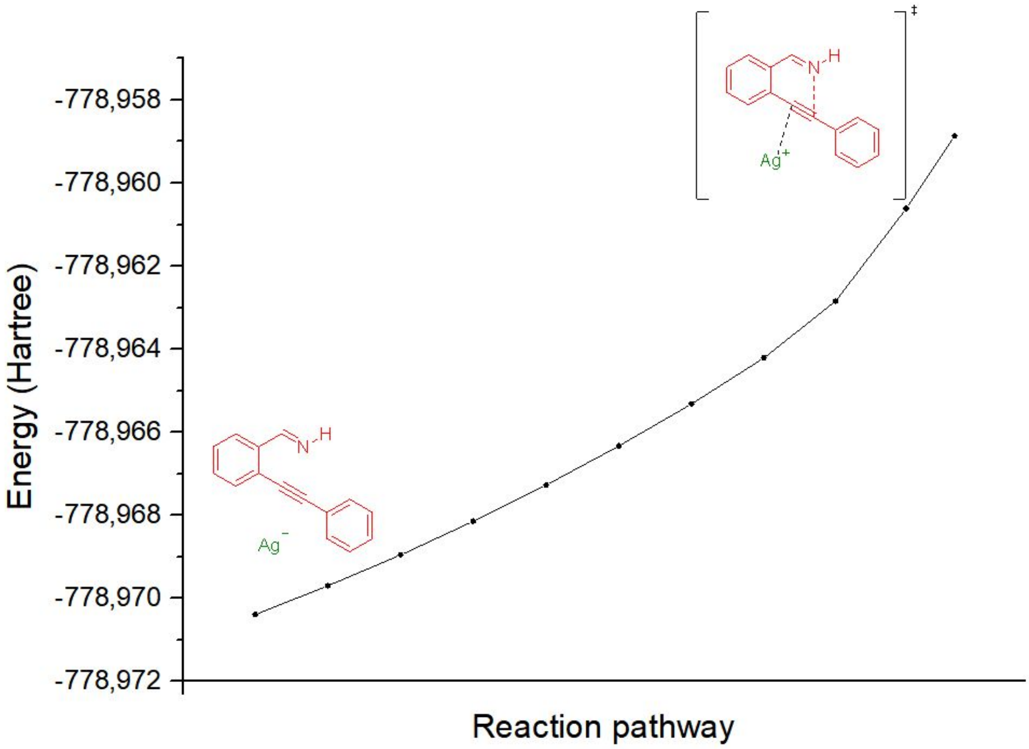

Figure S35. Intrinsic Reaction Coordinate – Step 4

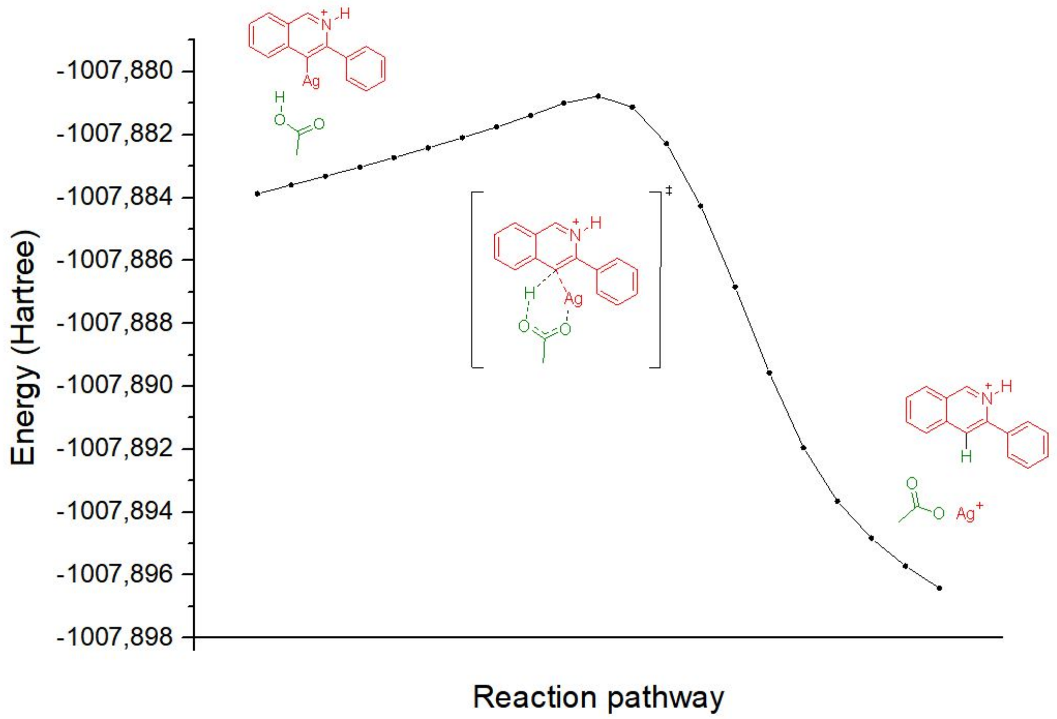

Figure S36. Intrinsic Reaction Coordinate – Alternative *5-exo-dig* cyclization

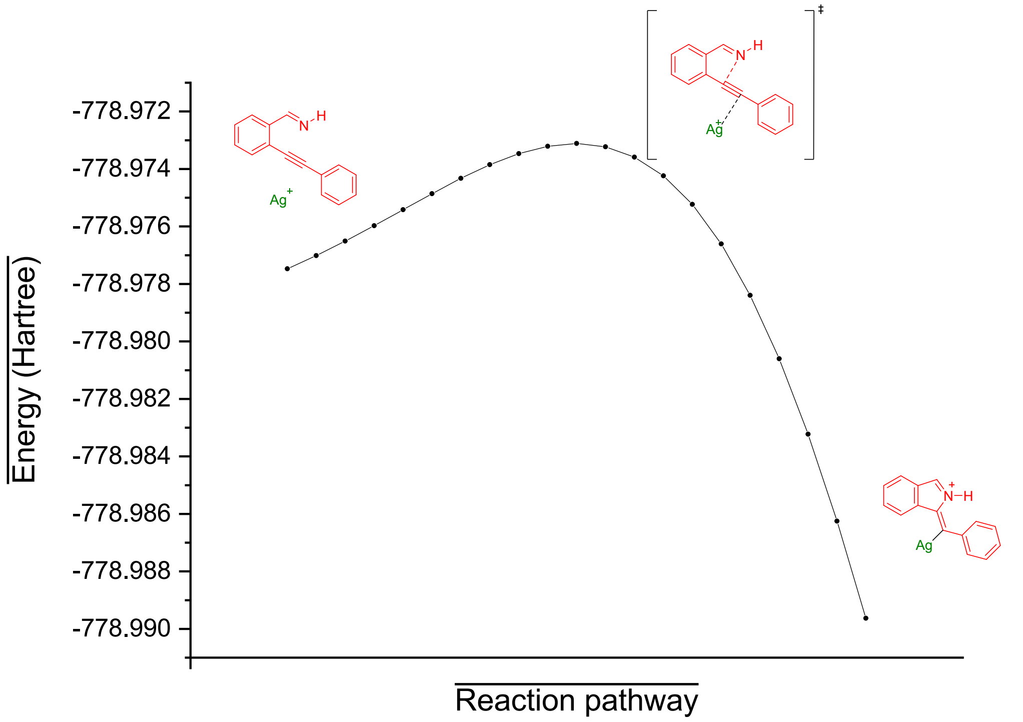

9.4 Electronic energies (E), enthalpies (H) and Gibbs free energies (G) of all optimized structures

Table S3. Electronic energies (E), enthalpies (H) and Gibbs free energies (G) variation. Values in Hartree.

Proposal		E	H	G
Step 1	Molecular complex (reagents)	-937.3700820	-937.0408790	-937.135381
	Transition state	-937.3685978	-937.0404078	-937.128646
	Molecular complex (products)	-937.3874583	-937.0565343	-937.141053
Step 2	Molecular complex (reagents)	-937.3769588	-937.0457768	-937.134082
	Transition state	-937.3550773	-937.0281643	-937.114329
	Molecular complex (products)	-937.3740699	-937.0456459	-937.136427
Step 3	Molecular complex (reagents)	-778.9801199	-778.7466589	-778.815974
	Transition state	-778.9736458	-778.7407018	-778.811032
	Molecular complex (products)	-779.0517704	-778.8148974	-778.881115
Step 4	Molecular complex (reagents)	-1.007.9143967	-1.007.6070347	-1.007.690568
	Transition state	-1.007.8807731	-1.007.5798411	-1.007.662511
	Molecular complex (products)	-1.007.9316840	-1.007.6248650	-1.007.711219
Step 5	Acetate	-228.3457221	-228.2911201	-228.328057
	Protonated product	-632.7078659	-632.4617019	-632.521486
	Total (reagents)	-861.0535880	-860.7528220	-860.849543
	Acetic acid	-228.8342612	-228.7663392	-228.804160
	Product	-632.2499791	-632.0174541	-632.076842
	Total (products)	-861.0842403	-860.7837933	-860.881002

Alternative *5-exo-dig* cyclization	Molecular complex (reagents)	-778.9791675	-778.7448655	-778.8171035
	Transition state	-778.9731154	-778.7416184	-778.8085024
	Molecular complex (products)	-779.0258380	-778.7901040	-778.8578410

9.5 Electronic energies (ΔE), enthalpies (ΔH) and Gibbs free energies (ΔG) variation along the reaction pathway

Table S1. Electronic energies (ΔE), enthalpies (ΔH) and Gibbs free energies (ΔG) variation. Values in kcal mol^{-1}.

Proposal		ΔE	ΔH	ΔG
Step 1	Molecular complex (reagents)	0.00	0.00	0.00
	Transition state	0.93	0.30	4.23
	Molecular complex (products)	-10.90	-9.82	-3.56
Step 2	Molecular complex (reagents)	0.00	0.00	0.00
	Transition state	13.73	11.05	12.39
	Molecular complex (products)	1.81	0.08	-1.47
Step 3	Molecular complex (reagents)	0.00	0.00	0.00
	Transition state	4.06	3.74	3.10
	Molecular complex (products)	-44.96	-42.82	-40.88
Step 4	Molecular complex (reagents)	0.00	0.00	0.00
	Transition state	21.10	17.06	17.61
	Molecular complex (products)	-10.85	-11.19	-12.96
Step 5	Total (reagents)	0.00	0.00	0.00
	Total (products)	-19.23	-19.43	-19.74
Alternative *5-exo-dig* cyclization	Molecular complex (reagents)	0.00	0.00	0.00
	Transition state	3.80	2.04	5.40
	Molecular complex (products)	-29.29	-28.39	-25.56

9.6 Coordinates of optimized stationary points

- Step 1 - Molecular complex (reagents)

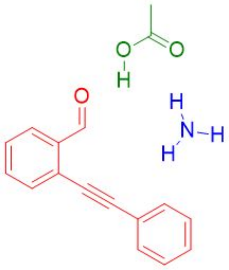

Symbolic Z-matrix:

Charge = 0 Multiplicity = 1

H	1.69981	2.98368	-0.53074
C	1.88476	0.57419	-0.34503
H	0.77345	0.66882	-0.46655
O	2.57017	1.5803	-0.31338
N	0.97151	0.67022	2.13743
H	1.7701	0.86477	2.75359
H	0.50641	1.56793	1.98687
O	-0.32512	3.15811	0.68951
C	0.09303	3.96409	-0.12035
H	-1.56476	5.31165	0.0788
C	-0.63309	5.22672	-0.49261
H	0.01278	6.09835	-0.30123
O	1.2321	3.82564	-0.78378
H	-0.85382	5.21724	-1.57181
H	0.32618	0.06113	2.67924
C	2.42047	-0.78081	-0.20008
C	1.55466	-1.909	-0.17973
C	3.80889	-0.96352	-0.15097
C	2.12683	-3.19835	-0.14318
C	4.35445	-2.24557	-0.10245
H	4.45371	-0.08639	-0.1568
C	3.51306	-3.35926	-0.11591
H	1.4733	-4.07097	-0.13304
H	5.44101	-2.37538	-0.07373
H	3.93581	-4.36795	-0.09347
C	0.13255	-1.76296	-0.16805
C	-1.07897	-1.62678	-0.13285
C	-2.491	-1.41714	-0.09395
C	-3.00242	-0.11236	0.08632
C	-3.3935	-2.49258	-0.23969
C	-4.37933	0.10294	0.11823
H	-2.31917	0.72617	0.20161
C	-4.76866	-2.26502	-0.20639
H	-3.00523	-3.5038	-0.3866
C	-5.26598	-0.96962	-0.02802
H	-4.77137	1.11637	0.258
H	-5.45835	-3.10502	-0.32099
H	-6.34461	-0.79544	-0.00321

- Step 1 - Transition state

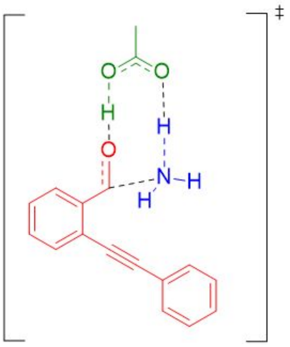

Symbolic Z-matrix:

Charge = 0 Multiplicity = 1

H	2.94518	1.7174	-0.64791
C	1.77031	-0.21107	-0.24063
H	0.89614	0.33548	-0.63369
O	2.9304	0.28559	-0.37433
N	1.29225	0.20694	1.61129
H	2.02745	-0.07997	2.24094
H	1.2813	1.2371	1.55297
O	1.7607	3.08146	0.95946
C	2.38196	3.50786	-0.01094
H	2.1699	5.59096	0.49213
C	2.52386	4.96314	-0.33736
H	3.57097	5.22526	-0.55038
O	2.98305	2.74259	-0.89071
H	1.94511	5.22436	-1.23512
H	0.40303	-0.14098	1.93287
C	1.65402	-1.70505	-0.15313
C	0.39442	-2.3424	-0.10945
C	2.8218	-2.47895	-0.13986
C	0.32643	-3.74721	-0.05963
C	2.7475	-3.87277	-0.08835
H	3.79052	-1.96761	-0.17908
C	1.49989	-4.50419	-0.04987
H	-0.6447	-4.23978	-0.03157
H	3.66072	-4.46623	-0.08201
H	1.44174	-5.59211	-0.0137
C	-0.80615	-1.58857	-0.10778
C	-1.83225	-0.94661	-0.10164
C	-3.02931	-0.18843	-0.09193
C	-2.97071	1.21833	-0.08422
C	-4.27676	-0.84075	-0.08912
C	-4.15349	1.96005	-0.07214
H	-2.00581	1.72405	-0.09022
C	-5.45204	-0.08701	-0.07731
H	-4.32041	-1.9289	-0.09795
C	-5.3928	1.31073	-0.06833
H	-4.10955	3.04884	-0.06636
H	-6.41827	-0.59025	-0.07591
H	-6.31235	1.89416	-0.05931

- Step 1 - Molecular complex (products)

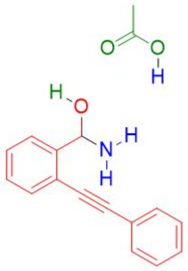

Symbolic Z-matrix:

Charge = 0 Multiplicity = 1

H	1.77881	2.82568	-0.49914
C	1.74256	0.63739	0.01837
H	0.75765	0.63722	-0.54555
O	2.52277	1.6435	-0.21858
N	1.14531	0.74922	1.50543
H	1.9123	0.76997	2.18479
H	0.60121	1.63113	1.51287
O	-0.29352	3.12651	0.75271
C	0.14043	3.90089	-0.10455
H	-1.51736	5.26425	0.0788
C	-0.58569	5.16352	-0.49261
H	0.06018	6.03515	-0.30123
O	1.2637	3.71504	-0.75218
H	-0.80642	5.15404	-1.57181
H	0.53158	-0.08107	1.68384
C	2.35727	-0.76501	-0.04208
C	1.53886	-1.9248	-0.11653
C	3.74569	-0.91612	-0.05617
C	2.14263	-3.19835	-0.17478
C	4.33865	-2.18237	-0.11825
H	4.35891	-0.00739	-0.0304
C	3.52886	-3.32766	-0.16331
H	1.5049	-4.08677	-0.22784
H	5.42521	-2.28058	-0.12113
H	3.98321	-4.32055	-0.20407
C	0.11675	-1.79456	-0.12065
C	-1.09477	-1.64258	-0.11705
C	-2.5068	-1.41714	-0.09395
C	-3.01822	-0.11236	0.08632
C	-3.4093	-2.49258	-0.23969
C	-4.39513	0.10294	0.11823
H	-2.31917	0.72617	0.20161
C	-4.78446	-2.26502	-0.20639
H	-3.02103	-3.5038	-0.3708
C	-5.28178	-0.96962	-0.02802
H	-4.77137	1.11637	0.258
H	-5.47415	-3.10502	-0.32099
H	-6.36041	-0.79544	-0.00321

- Step 2 – Molecular complex (reagents)

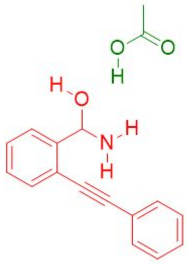

Symbolic Z-matrix:

Charge = 0 Multiplicity = 1

C	0.5276	2.96063	-0.61825
C	0.23761	1.73528	0.00557
C	1.25968	0.996	0.65542
C	2.55289	1.54065	0.68375
C	2.83407	2.75825	0.05275
C	1.83018	3.46651	-0.60971
H	-0.27786	3.51076	-1.1173
H	3.35155	1.00062	1.18917
H	3.85848	3.14959	0.06507
H	2.04585	4.41191	-1.10896
C	0.89722	-0.41545	1.2096
H	-0.06222	-0.28357	1.76043
O	0.77515	-1.26869	0.26051
C	-1.09562	1.20646	-0.03543
C	-2.20125	0.7016	-0.06938
C	-3.48381	0.07399	-0.10821
C	-4.63996	0.8043	-0.4559
C	-3.60261	-1.30044	0.19414
C	-5.88154	0.1712	-0.49709
H	-4.55437	1.86733	-0.69128
C	-4.84878	-1.92277	0.14869
H	-2.71518	-1.86911	0.46477
C	-5.99046	-1.19067	-0.19577
H	-6.77168	0.74515	-0.76657
H	-4.92994	-2.98718	0.38284
H	-6.96579	-1.68247	-0.23015
C	3.80916	-1.77344	-0.61384
O	4.10586	-1.45288	0.52925
C	4.89037	-2.11926	-1.61544
H	4.79128	-3.16912	-1.93163
H	4.80567	-1.49791	-2.51448
H	5.87873	-1.96774	-1.14485
O	2.57557	-1.84447	-1.08457
H	1.881	-1.54891	-0.44052
N	1.89015	-0.81471	2.21319
H	2.76016	-1.05747	1.74462
H	1.54491	-1.64405	2.7017
H	0.04972	-0.90344	-0.31966

- **Step 2 – Transition state**

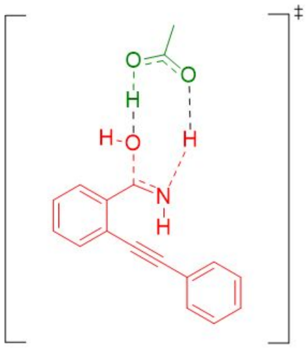

Symbolic Z-matrix:
Charge = 0 Multiplicity = 1

C	0.489	2.93305	-0.52903
C	0.20345	1.68592	0.06156
C	1.25134	0.95805	0.6921
C	2.54344	1.49778	0.70411
C	2.81241	2.72987	0.10604
C	1.78261	3.44933	-0.50697
H	-0.3189	3.48857	-1.00901
H	3.35333	0.94834	1.18497
H	3.82854	3.13027	0.12555
H	1.98739	4.41724	-0.97083
C	0.94298	-0.32875	1.37811
H	-0.07241	-0.39869	1.77464
O	0.60427	-1.46425	-0.04361
C	-1.12205	1.15805	0.00193
C	-2.23778	0.66784	-0.0586
C	-3.52888	0.0587	-0.11262
C	-4.67348	0.81147	-0.45115
C	-3.6705	-1.31648	0.17576
C	-5.92575	0.20013	-0.49528
H	-4.57028	1.87515	-0.67627
C	-4.92727	-1.91706	0.12791
H	-2.78521	-1.90322	0.43158
C	-6.05726	-1.16235	-0.20599
H	-6.80661	0.79168	-0.75706
H	-5.02619	-2.98199	0.3527
H	-7.04105	-1.63691	-0.24153
C	3.93173	-1.68181	-0.62567
O	4.13609	-1.4561	0.59012
C	5.14147	-1.85855	-1.53149
H	5.459	-0.86486	-1.89222
H	5.98322	-2.2969	-0.97631
H	4.89984	-2.47525	-2.40892
O	2.79196	-1.74519	-1.18803
H	1.56484	-1.59014	-0.51145
N	1.87904	-0.94631	2.10199
H	2.81182	-1.08629	1.65591
H	1.55901	-1.73408	2.6598
H	0.02904	-1.04552	-0.70757

- **Step 2 – Molecular complex (products)**

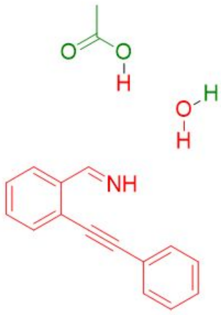

Symbolic Z-matrix:
Charge = 0 Multiplicity = 1

C	0.5276	2.93063	-0.61825
C	0.23761	1.72028	0.05057
C	1.30468	1.056	0.73042
C	2.59789	1.58565	0.68375
C	2.86407	2.77325	0.00775
C	1.81518	3.45151	-0.62471
H	-0.27786	3.45076	-1.1323
H	3.41155	1.07562	1.20417
H	3.87348	3.17959	0.00507
H	2.01585	4.39691	-1.13896
C	1.04722	-0.02545	1.6896
H	0.01278	-0.20857	2.03043
O	0.28015	-1.56869	-0.05449
C	-1.08062	1.19146	0.00958
C	-2.20125	0.7016	-0.05438
C	-3.48381	0.07399	-0.10821
C	-4.63996	0.8043	-0.4559
C	-3.60261	-1.30044	0.19414
C	-5.88154	0.1712	-0.49709
H	-4.55437	1.86733	-0.69128
C	-4.84878	-1.92277	0.14869
H	-2.70018	-1.86911	0.44977
C	-5.99046	-1.19067	-0.19577
H	-6.77168	0.74515	-0.76657
H	-4.92994	-2.98718	0.38284
H	-6.96579	-1.68247	-0.23015
C	3.82416	-1.77344	-0.64384
O	4.09086	-1.42288	0.55925
C	5.01037	-2.10426	-1.55544
H	4.92628	-3.15412	-1.88663
H	4.94067	-1.48291	-2.46948
H	5.98373	-1.95274	-1.08485
O	2.71057	-1.88947	-1.14457
H	1.041	-1.68391	-0.35052
N	1.96515	-0.84471	2.09319
H	2.91016	-0.95248	1.59462
H	1.69491	-1.52405	2.8217
H	-0.16028	-1.11344	-0.79966

- Step 3 - Molecular complex (reagents)

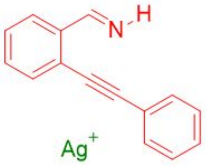

Symbolic Z-matrix:
Charge = 1 Multiplicity = 1

C	-4.68517	-1.0058	0.12424
C	-3.69578	-1.99388	0.09187
C	-2.33092	-1.69139	0.056
C	-1.96484	-0.31721	0.0399
C	-2.95415	0.67382	0.08054
C	-4.30946	0.33492	0.12006
H	-5.73917	-1.29128	0.15245
H	-3.99044	-3.05102	0.09651
H	-2.66213	1.72954	0.07646
H	-5.06627	1.12277	0.14511
C	-0.57265	-0.00284	-0.01548
Ag	0.08583	2.21758	-0.15644
C	0.72489	-0.05073	-0.01332
C	2.07052	-0.49888	0.0417
C	2.66761	-1.2478	-1.00942
C	2.84808	-0.09868	1.14479
C	4.03791	-1.51624	-0.96199
H	2.0528	-1.58666	-1.84525
C	4.2199	-0.36565	1.16922
H	2.37294	0.45783	1.95426
C	4.81193	-1.05507	0.10771
H	4.50846	-2.08165	-1.76919
H	4.82088	-0.02116	2.01172
H	5.89328	-1.24266	0.12482
C	-1.43291	-2.89158	0.02392
H	-2.05798	-3.81634	-0.0092
N	-0.14849	-2.92662	0.05237
H	0.09752	-3.96053	0.02642

- Step 3 – Transition state

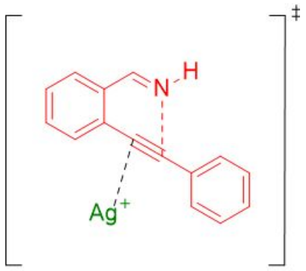

Symbolic Z-matrix:
Charge = 1 Multiplicity = 1

C	-4.50198	-1.21823	0.40508
C	-3.54895	-1.95389	-0.29786
C	-2.19511	-1.56235	-0.3026
C	-1.82217	-0.38649	0.39612
C	-2.7865	0.33171	1.13313
C	-4.11819	-0.08508	1.13568
H	-5.54991	-1.5338	0.39969
H	-3.86005	-2.85093	-0.84331
H	-2.49056	1.19621	1.73318
H	-4.86484	0.47089	1.71272
C	-0.44741	0.0464	0.35058
Ag	-0.20334	2.11214	-0.41823
C	0.71454	-0.39013	0.23407
C	2.12239	-0.53809	0.27695
C	2.87121	-0.72709	-0.90881
C	2.7774	-0.52539	1.53133
C	4.25438	-0.87569	-0.83468
H	2.36047	-0.76344	-1.87699
C	4.16226	-0.67735	1.58781
H	2.19583	-0.40511	2.4522
C	4.90049	-0.85204	0.41001
H	4.84063	-1.02104	-1.74753
H	4.67429	-0.67024	2.55566
H	5.988	-0.97579	0.46141
C	-1.22437	-2.44467	-0.99639
H	-1.68989	-3.32963	-1.47795
N	0.04765	-2.19605	-0.99731
H	0.67156	-2.86508	-1.45369

- Step 3 – Molecular complex (products)

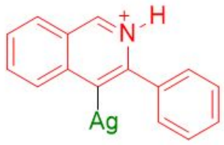

Symbolic Z-matrix:
Charge = 1 Multiplicity = 1

C	-4.65777	-1.0332	0.12424
C	-3.66838	-2.00758	0.09187
C	-2.30352	-1.65029	0.056
C	-1.92374	-0.27611	0.0536
C	-2.95415	0.68752	0.08054
C	-4.29576	0.32122	0.12006
H	-5.71177	-1.31868	0.15245
H	-3.93564	-3.06472	0.09651
H	-2.67583	1.74324	0.07646
H	-5.06627	1.09537	0.14511
C	-0.51785	0.16156	0.01192
Ag	0.07213	2.21758	-0.15644
C	0.53309	-0.68093	0.02778
C	2.00202	-0.71808	0.0417
C	2.68131	-1.3574	-1.00942
C	2.75218	-0.18088	1.11739
C	4.06531	-1.50254	-0.97569
H	2.1076	-1.76476	-1.84525
C	4.1377	-0.32455	1.14182
H	2.23594	0.32083	1.94056
C	4.79823	-1.00027	0.10771
H	4.57696	-2.01315	-1.79659
H	4.71128	0.07474	1.98432
H	5.87958	-1.13306	0.13852
C	-1.30961	-2.71348	0.03762
H	-1.67438	-3.76154	0.0182
N	-0.05259	-2.46082	0.03867
H	0.61812	-3.26183	0.02642

- **Step 4 – Molecular complex (reagents)**

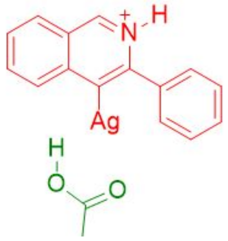

Symbolic Z-matrix:

Charge = 1 Multiplicity = 1

C	3.67708	2.73275	0.10793
C	2.46321	2.08512	0.2015
C	2.37213	0.66731	0.02891
C	3.5996	-0.05202	-0.22563
C	4.84482	0.6344	-0.30857
C	4.87933	2.00547	-0.14808
H	3.7188	3.82218	0.23363
H	1.54261	2.65051	0.40144
H	5.75863	0.05475	-0.50211
H	5.82982	2.54007	-0.21188
C	3.52219	-1.44482	-0.40947
H	4.39641	-2.07748	-0.62314
C	1.10997	-0.01406	0.13018
C	1.1344	-1.3911	-0.11283
C	-0.05631	-2.28205	-0.08218
C	-0.07437	-3.40811	0.78597
C	-1.1827	-2.03215	-0.92076
C	-1.19486	-4.24233	0.83654
H	0.77318	-3.61977	1.46551
C	-2.29997	-2.87028	-0.871
H	-1.17895	-1.19429	-1.6488
C	-2.31428	-3.9753	0.01076
H	-1.19245	-5.09858	1.53313
H	-3.16011	-2.6615	-1.53818
H	-3.19321	-4.62508	0.05044
C	-3.60572	2.05389	-0.21182
O	-2.72387	1.85262	0.65146
O	-3.40083	1.82193	-1.50671
C	-4.96731	2.58293	0.12398
H	-5.17701	3.48446	-0.48291
H	-5.72197	1.82861	-0.15912
H	-5.0375	2.80761	1.2035
Ag	-0.76659	0.89963	0.38533
H	-2.48105	1.47104	-1.64292
N	2.34117	-2.04389	-0.34635
H	2.31869	-3.05213	-0.53527

- **Step 4 – Transition state**

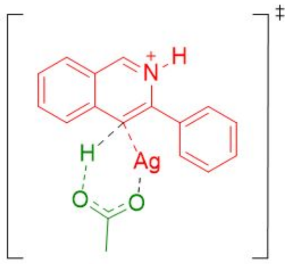

Symbolic Z-matrix:

Charge = 1 Multiplicity = 1

C	-4.22688	0.26017	-0.09559
C	-2.86345	0.46728	-0.09866
C	-1.95888	-0.60841	0.13288
C	-2.53059	-1.9071	0.37285
C	-3.94152	-2.1002	0.37261
C	-4.77344	-1.02981	0.14084
H	-4.90288	1.09939	-0.27739
H	-2.45249	1.46288	-0.28283
H	-4.34309	-3.09993	0.55463
H	-5.85717	-1.1657	0.13703
C	-1.64483	-2.96792	0.6031
H	-1.97868	-3.9864	0.81061
C	-0.53747	-0.40721	0.12903
C	0.258	-1.52849	0.34305
C	1.74346	-1.5366	0.32579
C	2.45456	-2.62132	-0.22155
C	2.46771	-0.45303	0.85719
C	3.85112	-2.61858	-0.24141
H	1.92111	-3.46597	-0.66588
C	3.86264	-0.45686	0.83886
H	1.93431	0.38221	1.31133
C	4.55992	-1.53721	0.28774
H	4.3856	-3.46412	-0.6813
H	4.40924	0.38722	1.26683
H	5.65267	-1.53603	0.27274
C	0.28961	2.94168	1.15749
O	0.58505	3.08354	-0.06291
O	-0.15538	1.7835	1.65036
C	0.39363	4.05164	2.1525
H	-0.59667	4.2302	2.60086
H	1.07124	3.7551	2.9685
H	0.76035	4.96629	1.67107
Ag	0.3075	1.18499	-1.50051
H	-0.24634	0.95823	0.94522
N	-0.33415	-2.7554	0.5818
H	0.27052	-3.55483	0.77988

- **Step 4 – Molecular complex (products)**

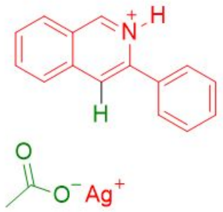

Symbolic Z-matrix:

Charge = 1 Multiplicity = 1

C	4.08244	1.88431	-0.69359
C	2.7579	1.50689	-0.81648
C	2.37823	0.16872	-0.53741
C	3.39322	-0.76418	-0.1333
C	4.74943	-0.34786	-0.02161
C	5.08388	0.95681	-0.29596
H	4.37073	2.91616	-0.90641
H	1.99351	2.22076	-1.12631

H	5.50577	-1.07286	0.28512
H	6.12117	1.28753	-0.20842
C	2.99949	-2.08525	0.12904
H	3.70854	-2.86458	0.4181
C	1.03359	-0.27457	-0.62514
C	0.69511	-1.58185	-0.32906
C	-0.67884	-2.12588	-0.35899
C	-1.0544	-3.17695	0.49912
C	-1.63879	-1.5865	-1.23836
C	-2.35424	-3.68182	0.4688
H	-0.34008	-3.59282	1.21583
C	-2.93654	-2.09835	-1.26405
H	-1.37061	-0.76992	-1.90963
C	-3.29767	-3.14677	-0.41297
H	-2.634	-4.49357	1.14373
H	-3.66812	-1.67858	-1.95884
H	-4.3148	-3.54674	-0.43589
C	-2.2603	2.32664	-0.86148
O	-2.42794	2.25836	0.40679
O	-1.27495	1.86567	-1.47958
C	-3.38719	3.00101	-1.63455
H	-2.98363	3.52239	-2.51635
H	-4.08489	2.21793	-1.99984
H	-3.96015	3.70082	-1.00898
Ag	-0.84918	1.17815	1.42372
H	0.25779	0.44467	-0.91228
N	1.72126	-2.44004	0.02253
H	1.49285	-3.42289	0.18603

- **Step 5 – Acetate**

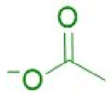

Symbolic Z-matrix:
Charge = -1 Multiplicity = 1

C	-1.80735	-6.30401	-0.92227
O	-1.82508	-7.24861	-0.0348
O	-1.10256	-5.22979	-0.75759
C	-2.62739	-6.45875	-2.18908
H	-1.97142	-6.37641	-3.05889
H	-3.14496	-7.41875	-2.20322
H	-3.35976	-5.64506	-2.24445

- **Step 5 – Protonated product**

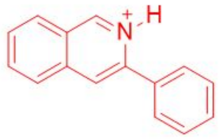

Symbolic Z-matrix:
Charge = 1 Multiplicity = 1

C	4.25351	0.54397	0.15177
C	2.88216	0.68469	0.05047
C	2.06187	-0.45605	-0.20639
C	2.70511	-1.73869	-0.30967
C	4.12294	-1.85629	-0.23986
C	4.88538	-0.7298	-0.01913
H	4.87062	1.41757	0.34718
H	2.40991	1.65443	0.15606
H	4.58513	-2.83335	-0.35145
H	5.96712	-0.79676	0.0421
C	1.88509	-2.86172	-0.51486
H	2.28139	-3.86068	-0.65929
C	0.63089	-0.31124	-0.39795
C	-0.11051	-1.49849	-0.43356
C	-1.5963	-1.57714	-0.40923
C	-2.25702	-2.69779	0.13698
C	-2.35717	-0.50708	-0.91625
C	-3.65938	-2.74928	0.15849
H	-1.7018	-3.51768	0.58645
C	-3.75716	-0.56141	-0.88671
H	-1.85764	0.36043	-1.3493
C	-4.41081	-1.68915	-0.3562
H	-4.15889	-3.62002	0.58127
H	-4.33852	0.26253	-1.28641
H	-5.49531	-1.73091	-0.3417
H	0.2625	0.673	-0.66602
N	0.55496	-2.73041	-0.52306
H	0.00109	-3.55968	-0.59741

- **Step 5 – Acetic acid**

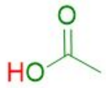

Symbolic Z-matrix:
Charge = 0 Multiplicity = 1

C	-1.80735	-6.30401	-0.92227
O	-1.82508	-7.24861	-0.0348
O	-1.10256	-5.22979	-0.75759
C	-2.62739	-6.45875	-2.18908
H	-1.97142	-6.37641	-3.05889
H	-3.14496	-7.41875	-2.20322
H	-3.35976	-5.64506	-2.24445
H	-0.16905	-5.4512	-0.79153

- **Step 5 – Product**

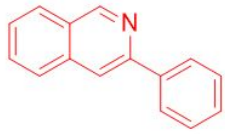

Symbolic Z-matrix:
Charge = 0 Multiplicity = 1

C	4.25351	0.54397	0.15177
C	2.88216	0.68469	0.05047
C	2.06187	-0.45605	-0.20639
C	2.70511	-1.73869	-0.30967
C	4.12294	-1.85629	-0.23986
C	4.88538	-0.7298	-0.01913
H	4.87062	1.41757	0.34718
H	2.40991	1.65443	0.15606
H	4.58513	-2.83335	-0.35145
H	5.96712	-0.79676	0.0421

C	1.88509	-2.86172	-0.51486
H	2.28139	-3.86068	-0.65929
C	0.63089	-0.31124	-0.39795
C	-0.11051	-1.49849	-0.43356
C	-1.5963	-1.57714	-0.40923
C	-2.25702	-2.69779	0.13698
C	-2.35717	-0.50708	-0.91625
C	-3.65938	-2.74928	0.15849
H	-1.7018	-3.51768	0.58645
C	-3.75716	-0.56141	-0.88671
H	-1.85764	0.36043	-1.3493
C	-4.41081	-1.68915	-0.3562
H	-4.15889	-3.62002	0.58127
H	-4.33852	0.26253	-1.28641
H	-5.49531	-1.73091	-0.3417
H	0.2625	0.673	-0.66602
N	0.55496	-2.73041	-0.52306

- Alternative *5-exo-dig* cyclization – Molecular complex (reagents)

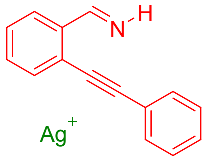

Symbolic Z-matrix:
Charge = 1 Multiplicity = 1

C	4.82257	0.41046	-0.08974
C	4.0892	1.57372	0.14543
C	2.68352	1.56809	0.17437
C	2.00641	0.32066	-0.04506
C	2.77155	-0.83434	-0.28717
C	4.15834	-0.79995	-0.30482
H	5.91452	0.43899	-0.09707
H	4.60346	2.5269	0.31557
H	2.25975	-1.78744	-0.46476
H	4.71788	-1.71789	-0.49083
C	0.59488	0.10746	-0.02878
Ag	-0.43736	-2.19803	-0.00299
C	-0.67128	0.09844	0.00172
C	-2.03544	0.52436	-0.05024
C	-3.08473	-0.16851	0.56774
C	-2.2832	1.75315	-0.69744
C	-4.37703	0.36345	0.5462
H	-2.88869	-1.1122	1.08576
C	-3.58069	2.26399	-0.72621
H	-1.45578	2.28779	-1.16018
C	-4.62612	1.57608	-0.1014
H	-5.1896	-0.17358	1.03269
H	-3.77728	3.21425	-1.22819
H	-5.64127	1.98808	-0.12645
C	1.95879	2.83287	0.42183
H	2.65625	3.69247	0.63899
N	0.67056	2.94149	0.4048
H	0.44223	3.99043	0.61049

- Alternative *5-exo-dig* cyclization – Transition state

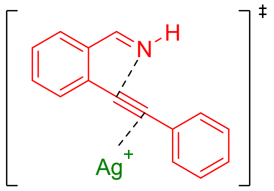

Symbolic Z-matrix:
Charge = 1 Multiplicity = 1

C	5.04068	-0.10503	0.18925
C	4.47129	1.13847	-0.10613
C	3.0804	1.27887	-0.12281
C	2.2495	0.16191	0.15283
C	2.83381	-1.08017	0.46453
C	4.2256	-1.20712	0.47609
H	6.13045	-0.21513	0.20146
H	5.11743	1.99557	-0.31847
H	2.21272	-1.94409	0.70813
H	4.68246	-2.17272	0.71609
C	0.83072	0.33892	0.1235
Ag	-1.09768	-1.96919	-0.26125
C	-0.39275	0.15246	0.12222
C	-1.73083	0.69253	0.16902
C	-2.41476	0.76981	1.40109
C	-2.34271	1.16668	-1.01133
C	-3.72057	1.26815	1.43382
H	-1.91886	0.47134	2.32959
C	-3.6501	1.65889	-0.9596
H	-1.79064	1.17463	-1.95551
C	-4.34347	1.69735	0.25592
H	-4.2542	1.33108	2.38803
H	-4.12975	2.02475	-1.87333
H	-5.36732	2.0816	0.28863
C	2.41942	2.58433	-0.39839
H	3.07494	3.43736	-0.63074
N	1.13244	2.55488	-0.31947
H	0.56932	3.38725	-0.47874

- Alternative *5-exo-dig* cyclization – Molecular complex (products)

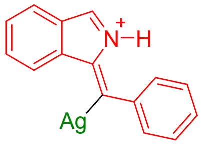

Symbolic Z-matrix:
Charge = 1 Multiplicity = 1

C	4.82257	0.38146	-0.08974
C	4.1617	1.58822	0.14543
C	2.77052	1.59709	0.17437
C	1.99191	0.43666	-0.01606
C	2.67005	-0.77634	-0.25817
C	4.07134	-0.78545	-0.29032
H	5.91452	0.35199	-0.12607
H	4.71947	2.5124	0.30107
H	2.11475	-1.70044	-0.42126
H	4.58738	-1.73239	-0.47633
C	0.53688	0.68746	0.05822
Ag	-0.43736	-2.18354	-0.00299

C	-0.61328	-0.03206	-0.01278
C	-1.97744	0.53886	-0.03574
C	-3.05573	-0.15401	0.56774
C	-2.2687	1.75315	-0.69744
C	-4.34803	0.36345	0.5462
H	-2.85969	-1.1122	1.05676
C	-3.56619	2.26399	-0.72621
H	-1.47028	2.28779	-1.21818
C	-4.61162	1.57608	-0.1014
H	-5.1606	-0.18808	1.03269
H	-3.76278	3.19975	-1.25719
H	-5.62677	1.97358	-0.12645
C	1.91529	2.74587	0.40733
H	2.27925	3.76497	0.63899
N	0.67056	2.41949	0.3178
H	-0.13777	3.09143	0.50899

10. REFERENCES

(1) Hao, T.; Huang, L.; Wei, Y.; Shi, M. Copper-Catalyzed Synthesis of Indolyl Benzo[b]Carbazoles and Their Photoluminescence Property. *Org. Lett.* **2021**, *23* (13), 5133–5137. https://doi.org/10.1021/acs.orglett.1c01659.

(2) Dell'Acqua, M.; Castano, B.; Cecchini, C.; Pedrazzini, T.; Pirovano, V.; Rossi, E.; Caselli, A.; Abbiati, G. Mild Regiospecific Synthesis of 1-Alkoxy-Isochromenes Catalyzed by Well-Defined [Silver(I)(Pyridine-Containing Ligand)] Complexes. *J. Org. Chem.* **2014**, *79* (8), 3494–3505. https://doi.org/10.1021/jo5002559.

(3) Too, P. C.; Chiba, S. A CuBr-Mediated Aerobic Reaction of 2-Alkynylbenzaldehydes and Primary Amines: Synthesis of 4-Bromoisoquinolones. *Chem. Commun.* **2012**, *48* (61), 7634. https://doi.org/10.1039/c2cc33426g.

(4) Kingston, C.; Palkowitz, M. D.; Takahira, Y.; Vantourout, J. C.; Peters, B. K.; Kawamata, Y.; Baran, P. S. A Survival Guide for the "Electro-Curious." *Acc. Chem. Res.* **2020**, *53* (1), 72–83. https://doi.org/10.1021/acs.accounts.9b00539.

(5) Ayoubi, M.; Nikbakht, A.; Amiri, K.; Abbasi Kejani, A.; Zahedian Tejeneki, H.; Balalaie, S. Efficient Synthesis of Isoquinoline Derivatives through Sequential Cyclization-Deoxygenation Reaction of 2-Alkynylbenzaldoximes. *SynOpen* **2021**, *6* (1), 11–15. https://doi.org/10.1055/s-0040-1719870.

(6) Shuai, S.; Mao, J.; Zhou, F.; Yan, Q.; Chen, L.; Li, J.; Walsh, P. J.; Liang, G. Base-Promoted Synthesis of Isoquinolines through a Tandem Reaction of 2-Methyl-Arylaldehydes and Nitriles. *J. Org. Chem.* **2024**, *89* (10), 6793–6797. https://doi.org/10.1021/acs.joc.4c00123.

(7) Zhao, Y.; Luo, M.; Li, Y.; Liu, X.; Tang, Z.; Deng, K.; Zhao, G. Efficient Synthesis of Isoquinolines by AgNO3-Catalyzed Sequential Imination-Annulation of 2-Alkynyl Aldehydes with Ammonium Bicarbonate. *Chinese J. Chem.* **2016**, *34* (9), 857–860. https://doi.org/10.1002/cjoc.201600277.

(8) Jeganathan, M.; Pitchumani, K. Synthesis of Substituted Isoquinolines via Iminoalkyne Cyclization Using Ag(i) Exchanged K10-Montmorillonite Clay as a Reusable Catalyst. *RSC Adv.* **2014**, *4* (73), 38491–38497. https://doi.org/10.1039/c4ra05026f.

(9) Dellacqua, M.; Abbiati, G.; Rossi, E. Palladium-Catalyzed, Microwave-Enhanced Three-Component Synthesis of Isoquinolines with Aqueous Ammonia. *Synlett* **2010**, No. 17, 2672–2676. https://doi.org/10.1055/s-0030-1258571.

(10) Zhang, R.; Pan, J.; Wu, Q. Copper Mediated One-Pot Multi-Component Synthesis of 3-Substitued Isoquinolines. *Chinese J. Org. Chem.* **2016**, *36* (12), 2906–2911. https://doi.org/10.6023/cjoc201606023.

(11) Hwang, S.; Lee, Y.; Lee, P. H.; Shin, S. AgOTf and TfOH Co-Catalyzed Isoquinoline Synthesis via Redox Reactions of O-Alkyl Oximes. *Tetrahedron Lett.* **2009**, *50* (20), 2305–2308. https://doi.org/10.1016/j.tetlet.2009.02.144.
